# Supplementary material for: Environmental contaminants drive insecticide resistance in Anopheles mosquitoes in Ghana
Source: Sci Rep. 2025 Sep 1;15:32201. doi: 10.1038/s41598-025-14239-x (PMC12402488; doi:10.1038/s41598-025-14239-x)

By Sample Quant Report

**Batch Path**	D:\MassHunter\Data\2024\Normal Analysis\September\No.14 (2024-09-12) MPS water and sediments\QuantResults\2024-10-01 water.batch.bin		
**Analysis Time**	10/1/2024 2:03:54 PM	**Analyst Name**	admin
**Report Time**	6/10/2025 10:50:02 AM	**Reporter Name**	admin
**Last Calib Update**	10/1/2024 2:03:53 PM	**Batch State**	Processed

**Analysis Info**			
**Instrument**	LCMS	**Operator**	
**Data File**	1360-PES2-24.d	**Sample Name**	1360-PES2-24
**Sample Type**	Sample	**Dilution**	0.003
**Acq. Method**	Pesticides_MRM_EN-15662_2024-06-27.m	**Acq. Date**	9/13/2024 9:13:19 AM
**Position**	P1-B8		-1

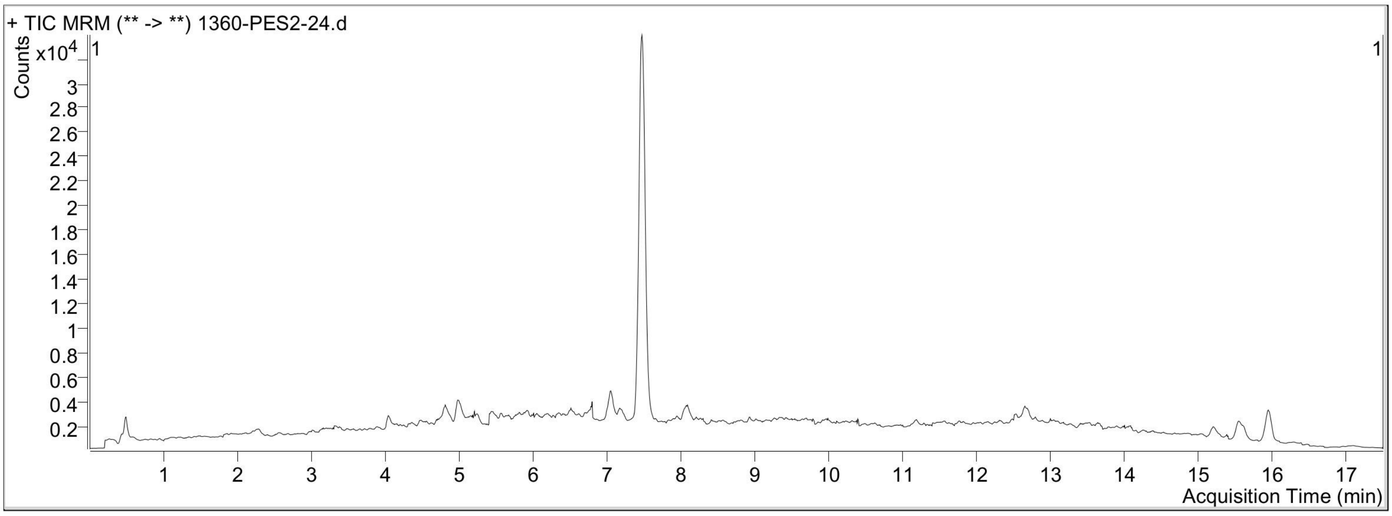

Quantitation Results

Compound	RT	Ref RT	Transition(T)	Transition(Q)	T-Resp	Q-Resp	QRatio	Ref QRatio	Final Conc.	Units
Methamidophos	0.49	0.76	142.0 -> 93.9	142.0 -> 124.9	0	807		31.6	0.00	ng/ml
Acephate	1.01	0.99	184.0 -> 94.6	184.0 -> 95.0	0	23		69.3	0.00	ng/ml
Carbendazim	2.27	2.31	192.1 -> 160.1	192.1 -> 132.1	0	475		16.8	0.00	ng/ml
Methomyl	3.31	3.34	163.1 -> 88.0	163.1 -> 106.0	0	130		64.3	0.00	ng/ml
Monocrotophos	4.22	3.85	224.1 -> 127.0	224.1 -> 58.0	0			51.6	0.00	ng/ml
Thiamethoxam	4.26	4.19	292.0 -> 211.1	292.0 -> 181.1	0	38		47.7	0.00	ng/ml
Clothianidin	4.60	4.59	250.0 -> 169.0	250.0 -> 131.9	0			77.4	0.00	ng/ml
Imidacloprid	4.71	4.75	256.0 -> 175.0	256.0 -> 208.9	0	642		86.8	0.00	ng/ml
Dimethoate	4.81	4.85	230.0 -> 198.8	230.0 -> 125.0	0	310		99.7	0.00	ng/ml
Acetamiprid	4.98	5.02	223.1 -> 126.0	223.1 -> 56.0	3857	1904	49.4	45.9	0.02	ng/ml
Sulfoxaflor	5.93	5.72	278.0 -> 174.0	278.0 -> 154.0	495	44	8.9	44.5	0.01	ng/ml
Amicarbazone	5.98	5.86	242.2 -> 143.1	242.2 -> 54.9	0			4.0	0.00	ng/ml
Ametryn	5.91	5.95	228.1 -> 186.1	228.1 -> 91.1	0	334		22.9	0.00	ng/ml
Bensulfuron-methyl	6.13	6.17	411.1 -> 182.1	411.1 -> 149.1	0	953		36.8	0.00	ng/ml
Nicosulfuron	6.12	6.17	411.1 -> 182.0	411.1 -> 181.9	0	315		100.0	0.00	ng/ml
Pyrimethanil	6.40	6.44	200.1 -> 82.0	200.1 -> 106.9	0	156		90.0	0.00	ng/ml
Terbutryn	7.18	7.23	242.1 -> 186.1	242.1 -> 68.1	0	13668		31.4	0.00	ng/ml
Atrazine	7.47	7.53	216.1 -> 174.1	216.1 -> 68.0	118873	70773	59.5	61.0	0.13	ng/ml
Spiroxamine	8.04	8.10	298.3 -> 144.1	298.3 -> 100.1	0	84		56.0	0.00	ng/ml
Metalaxyl	8.08	8.14	280.2 -> 220.1	280.2 -> 160.1	0	3304		85.4	0.00	ng/ml
Triadimenol	9.76	9.69	296.1 -> 70.0	296.1 -> 99.1	0	45		4.6	0.00	ng/ml
Tebuconazole	9.63	9.81	308.1 -> 70.0	308.1 -> 124.9	0	22		1.1	0.00	ng/ml
Prochloraz	9.71	9.82	376.0 -> 308.0	376.0 -> 265.9	0	51		12.8	0.00	ng/ml
Dimethomorph	9.93	9.85	388.1 -> 165.1	388.1 -> 301.1	0			96.7	0.00	ng/ml
Promecarb	10.30	10.24	208.1 -> 109.1	208.1 -> 151.1	0	35		95.6	0.00	ng/ml
Triadimefon	10.70	10.85	294.1 -> 197.2	294.1 -> 225.1	0			1.0	0.00	ng/ml
Boscalid	10.97	10.93	343.0 -> 307.1	343.0 -> 271.2	0	16		50.8	0.00	ng/ml
Metolachlor	11.48	11.53	284.1 -> 252.1	284.1 -> 176.1	0	155		49.4	0.00	ng/ml
Emamectin benzoate	11.64	11.62	886.5 -> 158.0	886.5 -> 302.4	0	110		1.3	0.00	ng/ml
Azinphos-Ethyl	11.90	12.10	346.1 -> 97.0	346.1 -> 137.0	0	16		69.7	0.00	ng/ml

Quantitation Results

Tebufenozide	12.31	12.20	353.2 -> 133.1	353.2 -> 297.2	0			38.8	0.00	ng/ml
Chlorfenvinphos	12.36	12.41	359.0 -> 99.0	359.0 -> 170.0	0			56.0	0.00	ng/ml
Aclonifen	12.47	12.47	265.0 -> 248.0	265.0 -> 182.1	0	7		69.4	0.00	ng/ml
Pirimifos-methyl	12.54	12.58	306.2 -> 164.1	306.2 -> 108.1	0	928		56.3	0.00	ng/ml
Kresoxim methyl	12.68	12.71	314.1 -> 222.1	314.1 -> 267.0	0	60		92.4	0.00	ng/ml
Difenoconazole	12.79	12.82	406.1 -> 251.0	406.1 -> 337.0	0	40		12.9	0.00	ng/ml
Fluopyram	12.93	13.06	397.0 -> 173.0	397.0 -> 145.0	0			68.3	0.00	ng/ml
Trifloxystrobin	14.44	14.46	409.1 -> 186.0	409.1 -> 145.0	0	334		49.9	0.00	ng/ml
Indoxacarb	14.40	14.48	528.1 -> 150.0	528.1 -> 203.0	0	23		90.3	0.00	ng/ml
Propaquizafop	14.71	14.72	444.1 -> 100.1	444.1 -> 299.2	0	10		14.0	0.00	ng/ml
Allethrin	15.18	15.24	303.2 -> 135.0	303.2 -> 123.0	0	307		39.0	0.00	ng/ml
Pyriproxyfen	15.21	15.25	322.2 -> 96.0	322.2 -> 185.0	2827	607	21.5	17.7	0.00	ng/ml
Fluazinam	15.31	15.29	465.0 -> 373.0	465.0 -> 338.0	0	12		13.5	0.00	ng/ml
Chlorpyrifos	15.54	15.58	349.9 -> 97.0	349.9 -> 198.0	0	2321		76.8	0.00	ng/ml

Compound Graphics

Methamidophos

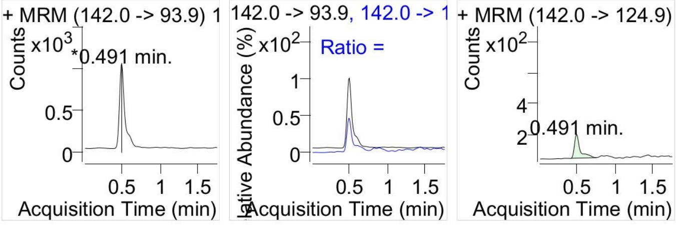

Acephate

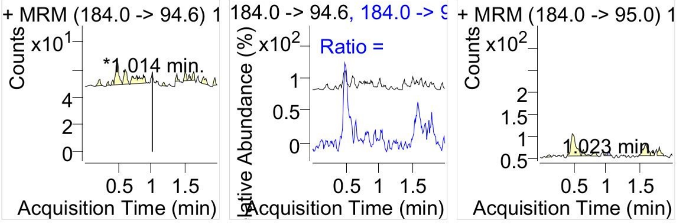

Carbendazim

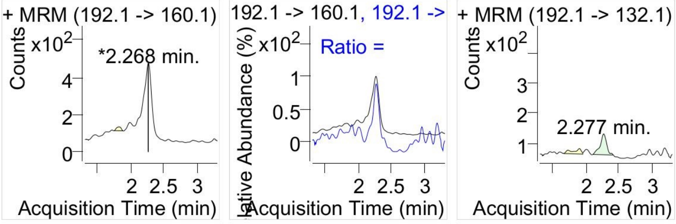

Methomyl

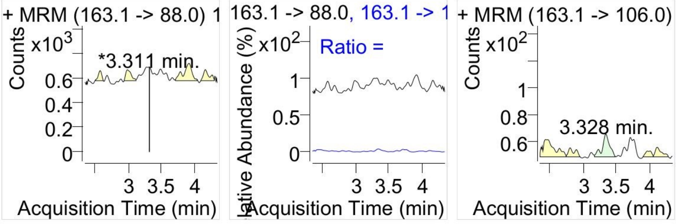

Monocrotophos

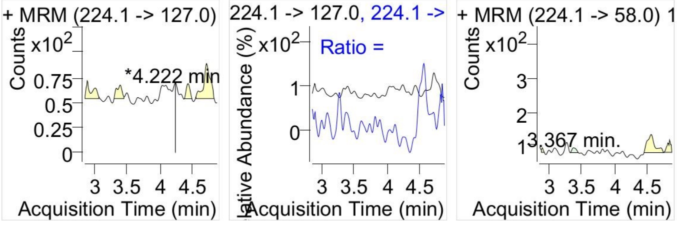

Thiamethoxam

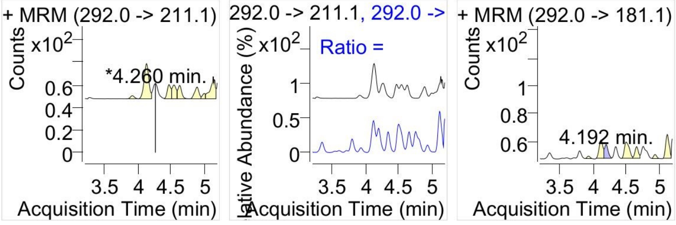

Clothianidin

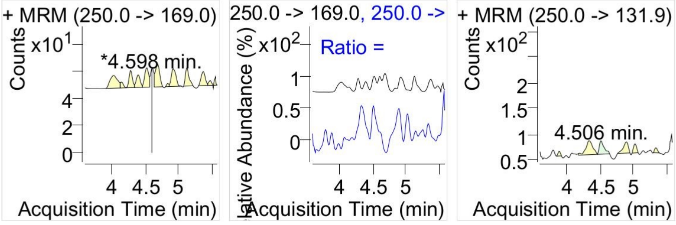

Imidacloprid

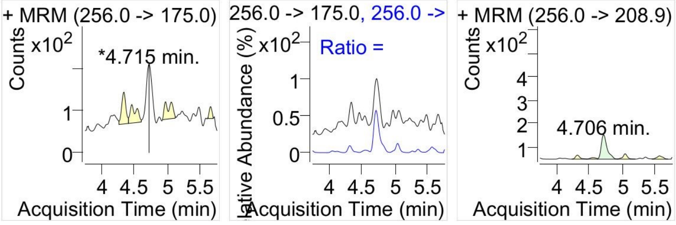

Dimethoate

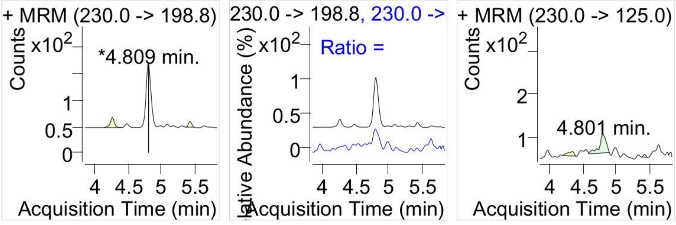

Acetamiprid

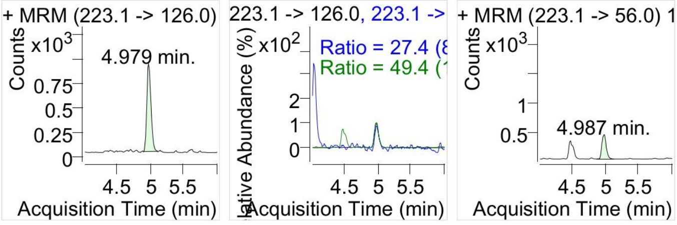

Sulfoxaflor

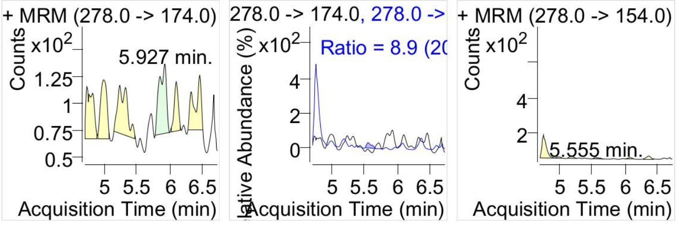

Amicarbazone

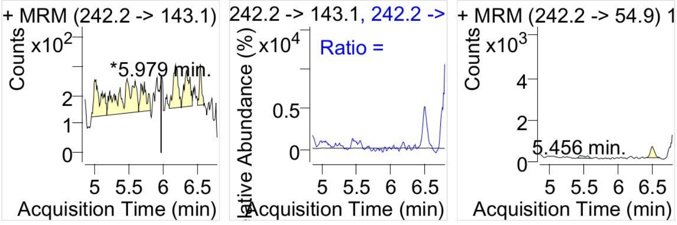

Ametryn

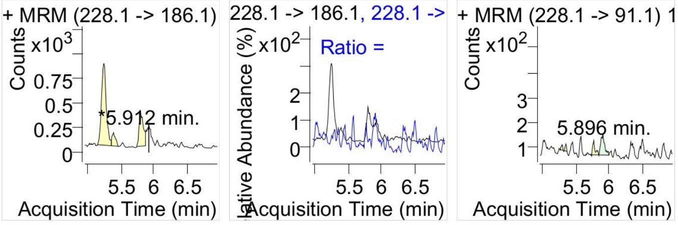

Bensulfuron-methyl

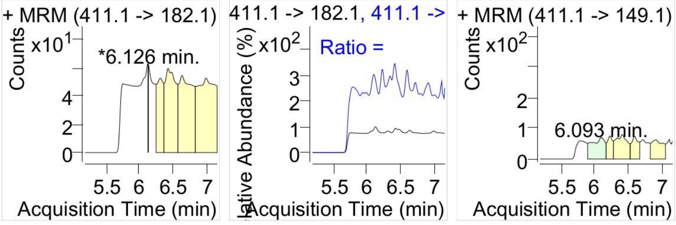

Nicosulfuron

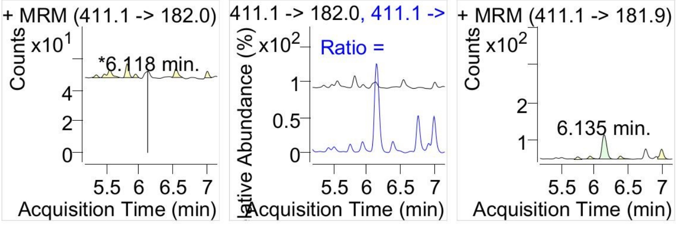

Pyrimethanil

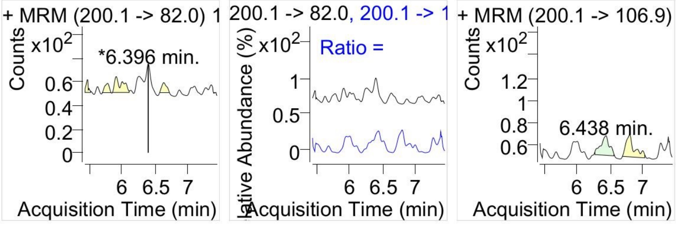

Terbutryn

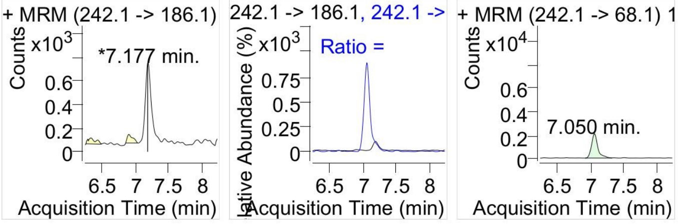

Atrazine

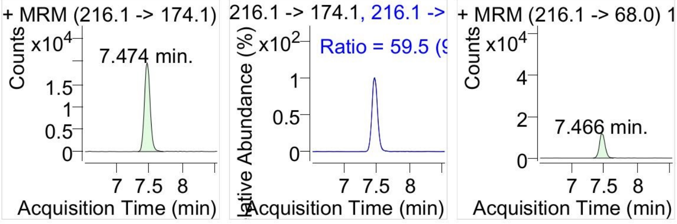

Spiroxamine

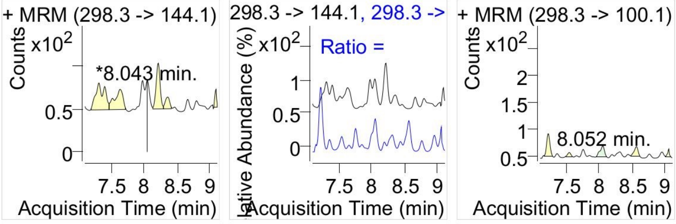

Metalaxyl

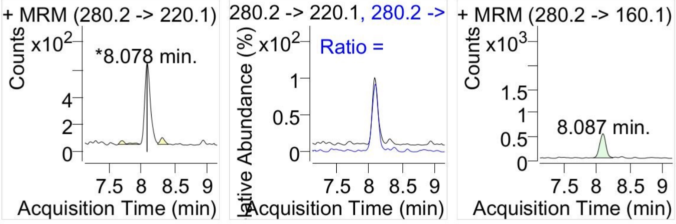

Triadimenol

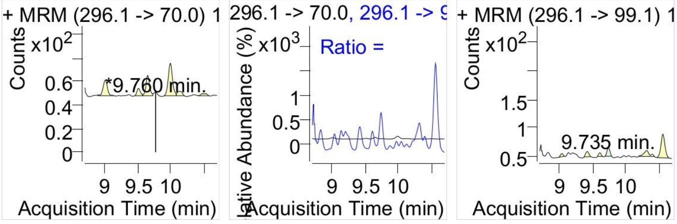

Tebuconazole

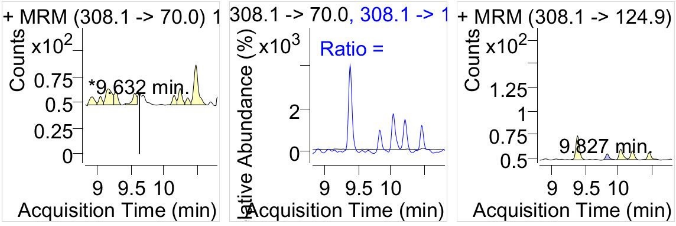

Prochloraz

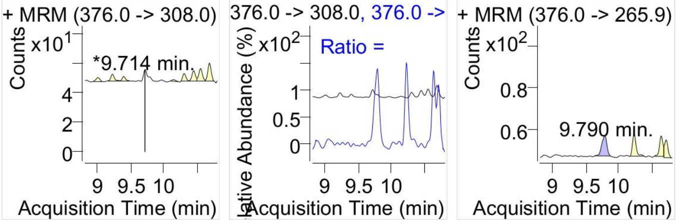

Dimethomorph

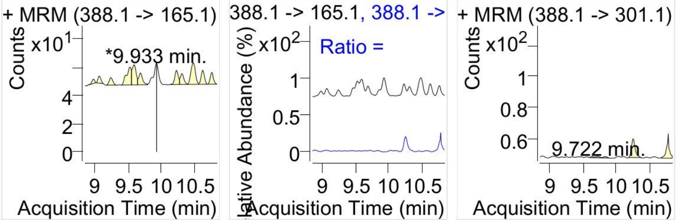

 Generated at 10:50 AM on 6/10/2025

By Sample Quant Report

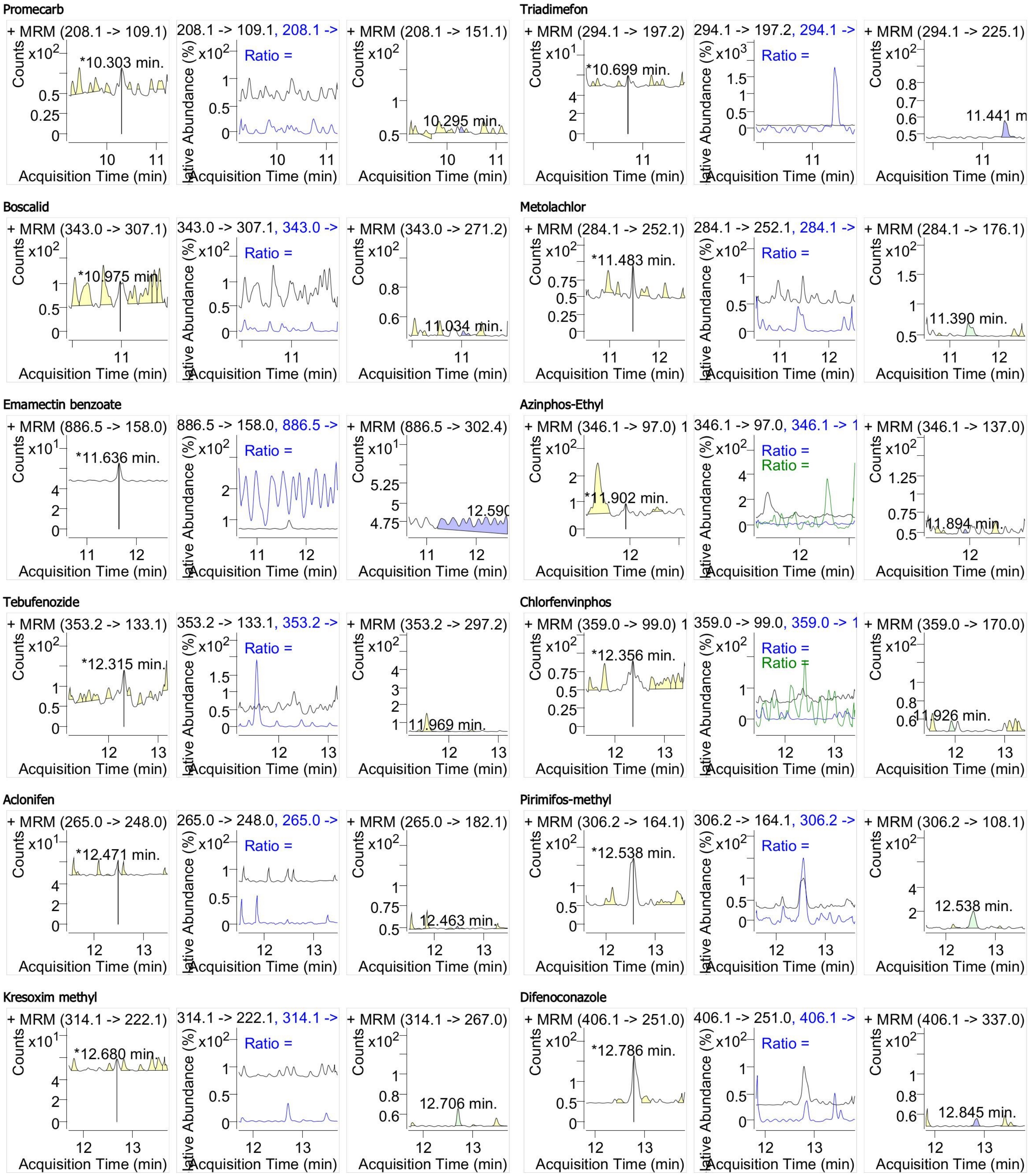

By Sample Quant Report

Fluopyram

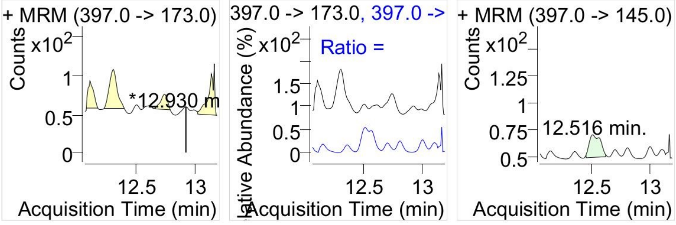

Trifloxystrobin

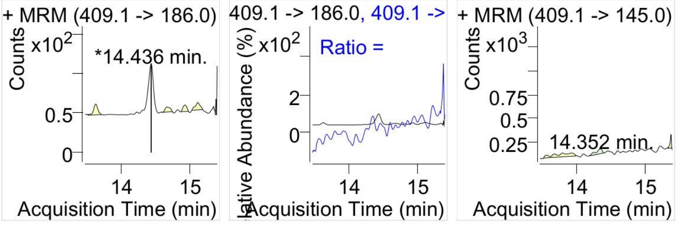

Indoxacarb

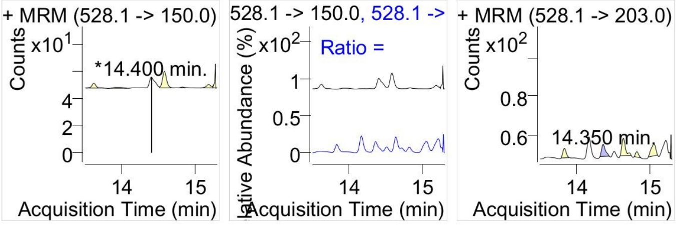

Propaquizafop

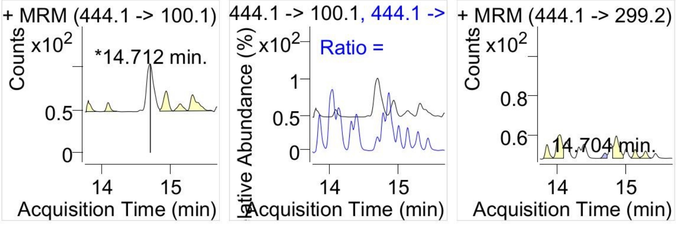

Allethrin

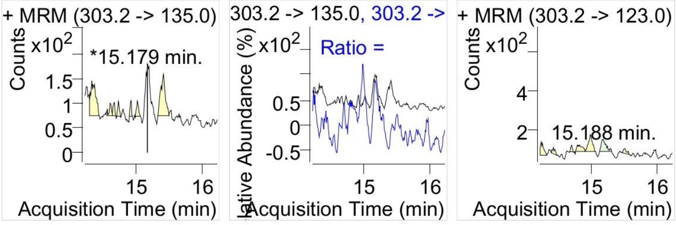

Pyriproxyfen

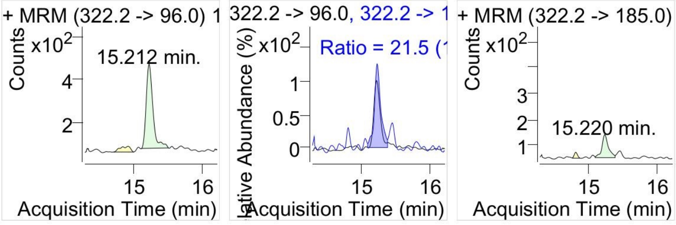

Fluazinam

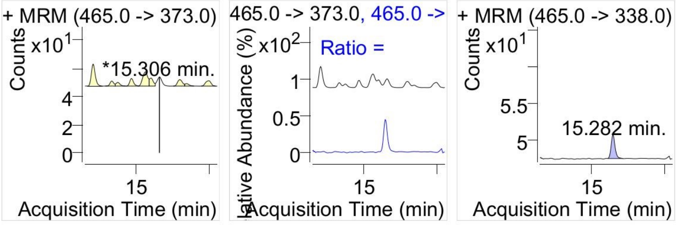

Chlorpyrifos

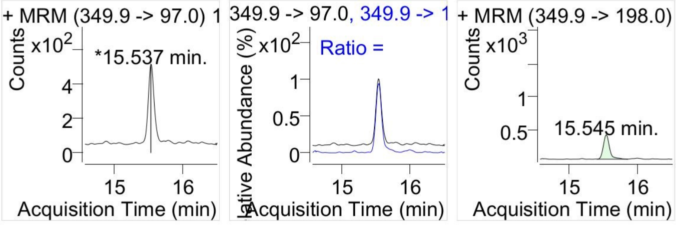

By Sample Quant Report

Analysis Info

**Instrument**	LCMS	**Operator**	
**Data File**	1361-PES2-24.d	**Sample Name**	1361-PES2-24
**Sample Type**	Sample	**Dilution**	0.003
**Acq. Method**	Pesticides_MRM_EN-15662_2024-06-27.m	**Acq. Date**	9/13/2024 9:36:09 AM
**Position**	P1-B9		-1

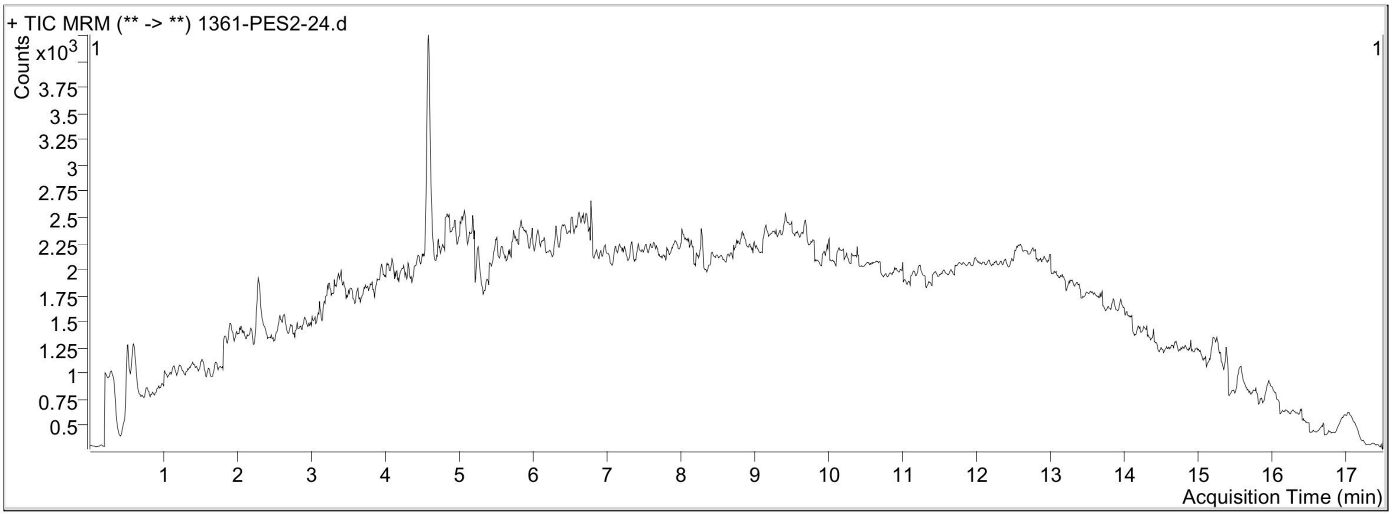

Quantitation Results

Compound	RT	Ref RT	Transition(T)	Transition(Q)	T-Resp	Q-Resp	QRatio	Ref QRatio	Final Conc.	Units
Methamidophos	0.59	0.76	142.0 -> 93.9	142.0 -> 124.9	0	35		31.6	0.00	ng/ml
Acephate	0.95	0.99	184.0 -> 94.6	184.0 -> 95.0	0	42		69.3	0.00	ng/ml
Carbendazim	2.29	2.31	192.1 -> 160.1	192.1 -> 132.1	0	388		16.8	0.00	ng/ml
Methomyl	3.41	3.34	163.1 -> 88.0	163.1 -> 106.0	0	31		64.3	0.00	ng/ml
Monocrotophos	3.84	3.85	224.1 -> 127.0	224.1 -> 58.0	0			51.6	0.00	ng/ml
Thiamethoxam	4.05	4.19	292.0 -> 211.1	292.0 -> 181.1	0	36		47.7	0.00	ng/ml
Clothianidin	4.58	4.59	250.0 -> 169.0	250.0 -> 131.9	3956	3641	92.0	77.4	0.05	ng/ml
Imidacloprid	4.77	4.75	256.0 -> 175.0	256.0 -> 208.9	0	4		86.8	0.00	ng/ml
Dimethoate	4.84	4.85	230.0 -> 198.8	230.0 -> 125.0	0	86		99.7	0.00	ng/ml
Acetamiprid	5.01	5.02	223.1 -> 126.0	223.1 -> 56.0	0	76		45.9	0.00	ng/ml
Sulfoxaflor	5.73	5.72	278.0 -> 174.0	278.0 -> 154.0	0			44.5	0.00	ng/ml
Amicarbazone	5.82	5.86	242.2 -> 143.1	242.2 -> 54.9	0			4.0	0.00	ng/ml
Ametryn	5.84	5.95	228.1 -> 186.1	228.1 -> 91.1	0	69		22.9	0.00	ng/ml
Bensulfuron-methyl	6.08	6.17	411.1 -> 182.1	411.1 -> 149.1	0			36.8	0.00	ng/ml
Nicosulfuron	5.90	6.17	411.1 -> 182.0	411.1 -> 181.9	0	29		100.0	0.00	ng/ml
Pyrimethanil	6.32	6.44	200.1 -> 82.0	200.1 -> 106.9	0	71		90.0	0.00	ng/ml
Terbutryn	7.42	7.23	242.1 -> 186.1	242.1 -> 68.1	0	125		31.4	0.00	ng/ml
Atrazine	7.53	7.53	216.1 -> 174.1	216.1 -> 68.0	0			61.0	0.00	ng/ml
Spiroxamine	8.13	8.10	298.3 -> 144.1	298.3 -> 100.1	0	46		56.0	0.00	ng/ml
Metalaxyl	8.13	8.14	280.2 -> 220.1	280.2 -> 160.1	0	57		85.4	0.00	ng/ml
Triadimenol	9.72	9.69	296.1 -> 70.0	296.1 -> 99.1	0	30		4.6	0.00	ng/ml
Tebuconazole	9.83	9.81	308.1 -> 70.0	308.1 -> 124.9	0	4		1.1	0.00	ng/ml
Prochloraz	9.79	9.82	376.0 -> 308.0	376.0 -> 265.9	0			12.8	0.00	ng/ml
Dimethomorph	9.93	9.85	388.1 -> 165.1	388.1 -> 301.1	0	5		96.7	0.00	ng/ml
Promecarb	10.30	10.24	208.1 -> 109.1	208.1 -> 151.1	0			95.6	0.00	ng/ml
Triadimefon	10.77	10.85	294.1 -> 197.2	294.1 -> 225.1	0	34		1.0	0.00	ng/ml
Boscalid	11.03	10.93	343.0 -> 307.1	343.0 -> 271.2	0			50.8	0.00	ng/ml
Metolachlor	11.43	11.53	284.1 -> 252.1	284.1 -> 176.1	0	38		49.4	0.00	ng/ml
Emamectin benzoate	11.64	11.62	886.5 -> 158.0	886.5 -> 302.4	0	4		1.3	0.00	ng/ml
Azinphos-Ethyl	12.37	12.10	346.1 -> 97.0	346.1 -> 137.0	0	27		69.7	0.00	ng/ml
Tebufenozide	12.33	12.20	353.2 -> 133.1	353.2 -> 297.2	0	57		38.8	0.00	ng/ml
Chlorfenvinphos	12.47	12.41	359.0 -> 99.0	359.0 -> 170.0	0			56.0	0.00	ng/ml
Aclonifen	12.44	12.47	265.0 -> 248.0	265.0 -> 182.1	0	4		69.4	0.00	ng/ml
Pirimifos-methyl	12.55	12.58	306.2 -> 164.1	306.2 -> 108.1	0	81		56.3	0.00	ng/ml
Kresoxim methyl	12.76	12.71	314.1 -> 222.1	314.1 -> 267.0	0	33		92.4	0.00	ng/ml
Difenoconazole	12.81	12.82	406.1 -> 251.0	406.1 -> 337.0	0	6		12.9	0.00	ng/ml

By Sample Quant Report

Quantitation Results

Fluopyram	13.04	13.06	397.0 -> 173.0	397.0 -> 145.0	0	34		68.3	0.00	ng/ml
Trifloxystrobin	14.43	14.46	409.1 -> 186.0	409.1 -> 145.0	0	158		49.9	0.00	ng/ml
Indoxacarb	14.50	14.48	528.1 -> 150.0	528.1 -> 203.0	0	15		90.3	0.00	ng/ml
Propaquizafop	14.70	14.72	444.1 -> 100.1	444.1 -> 299.2	0	13		14.0	0.00	ng/ml
Allethrin	15.30	15.24	303.2 -> 135.0	303.2 -> 123.0	0	18		39.0	0.00	ng/ml
Pyriproxyfen	15.21	15.25	322.2 -> 96.0	322.2 -> 185.0	1228	253	20.6	17.7	0.00	ng/ml
Fluazinam	15.25	15.29	465.0 -> 373.0	465.0 -> 338.0	0	0		13.5	0.00	ng/ml
Chlorpyrifos	15.57	15.58	349.9 -> 97.0	349.9 -> 198.0	0	391		76.8	0.00	ng/ml

Compound Graphics

Methamidophos

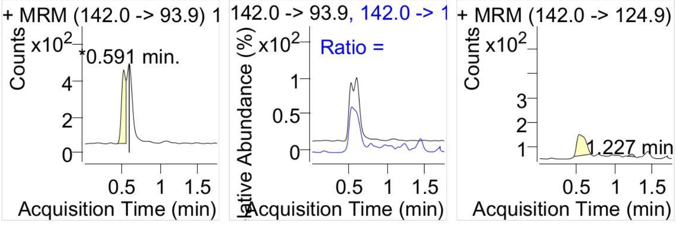

Acephate

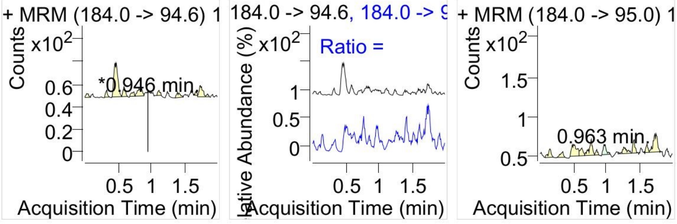

Carbendazim

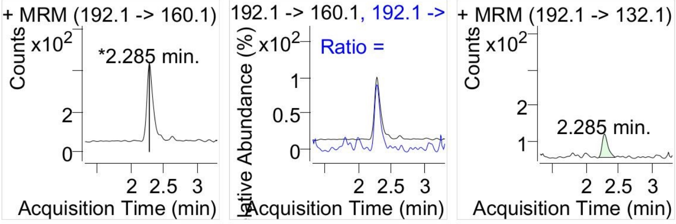

Methomyl

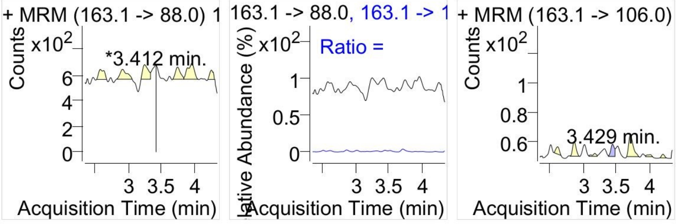

Monocrotophos

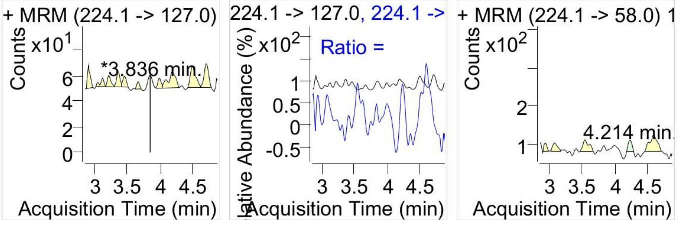

Thiamethoxam

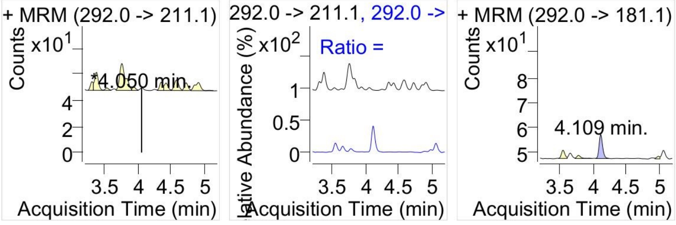

Clothianidin

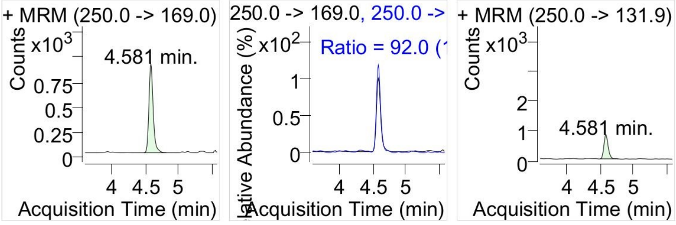

Imidacloprid

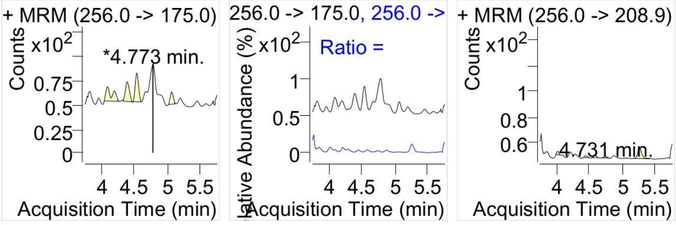

Dimethoate

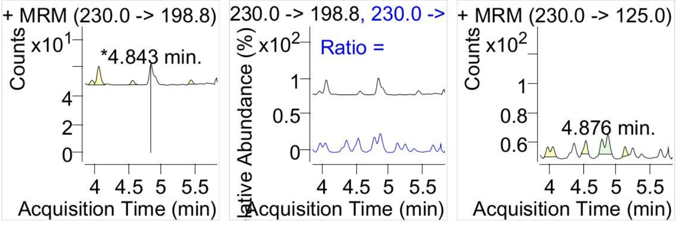

Acetamiprid

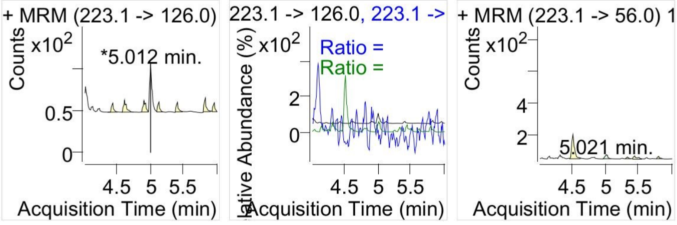

Sulfoxaflor

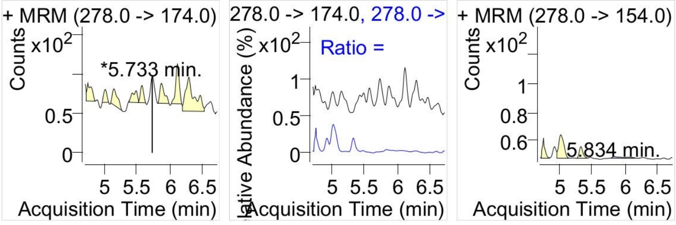

Amicarbazone

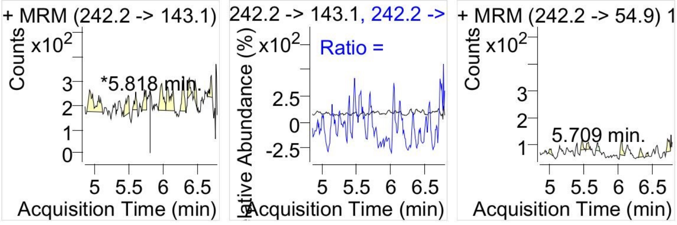

Ametryn

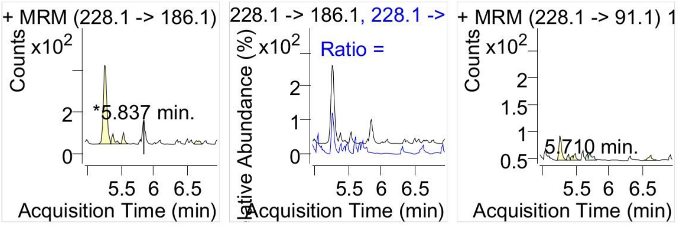

Bensulfuron-methyl

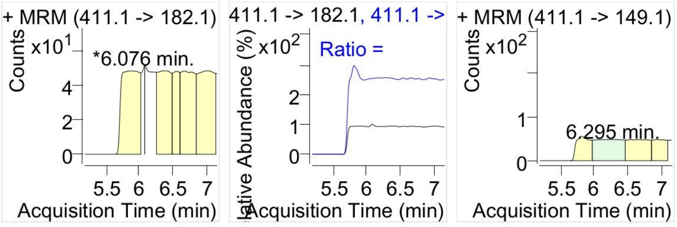

Nicosulfuron

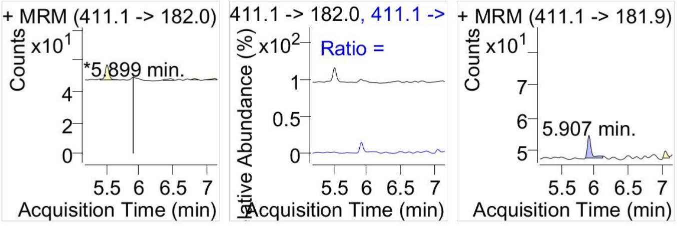

Pyrimethanil

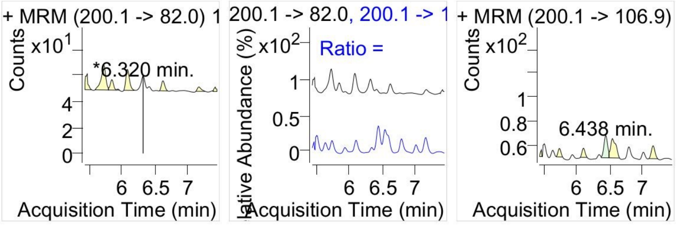

Terbutryn

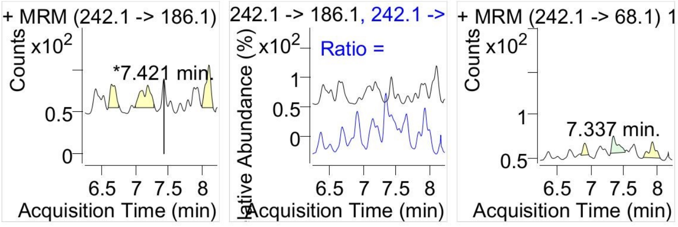

Atrazine

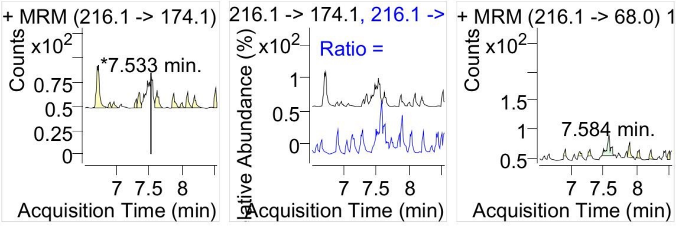

Spiroxamine

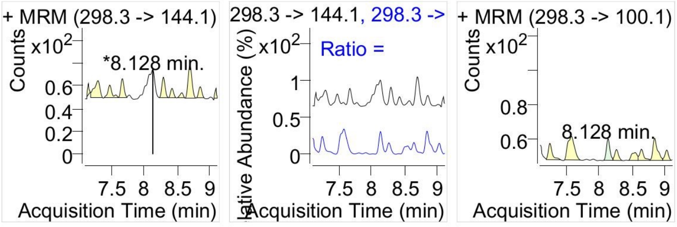

Metalaxyl

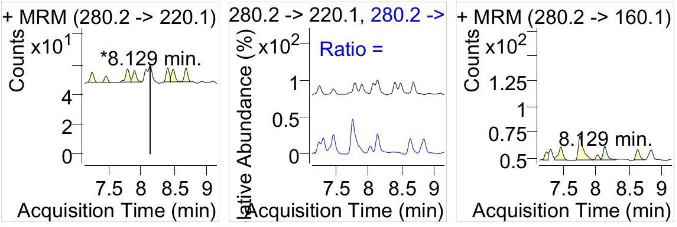

Triadimenol

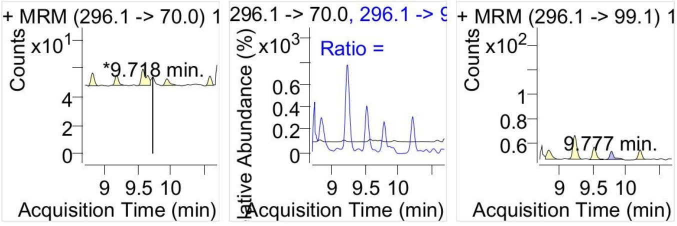

Tebuconazole

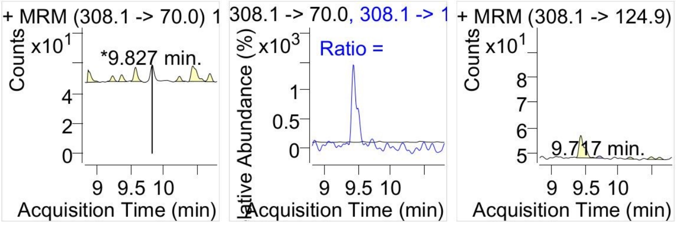

Prochloraz

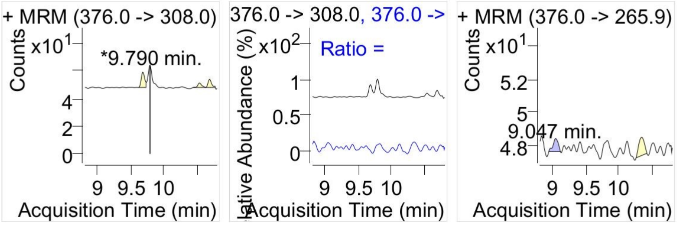

Dimethomorph

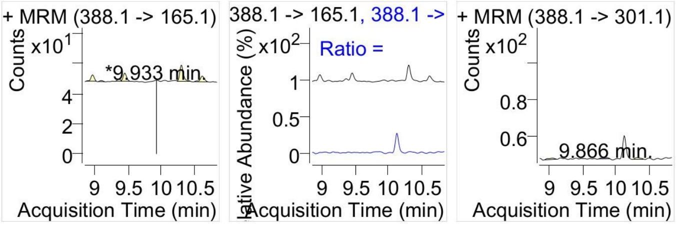

Promecarb

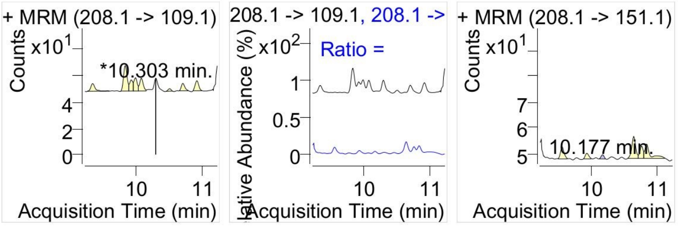

Triadimefon

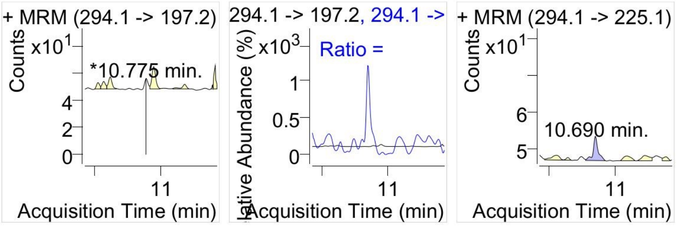

Boscalid

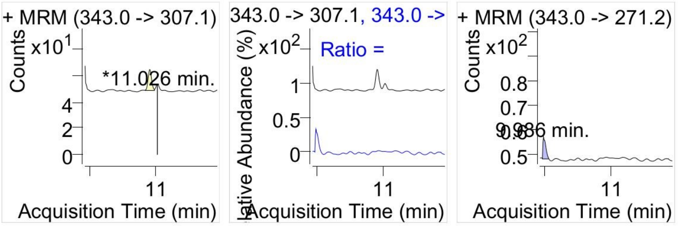

Metolachlor

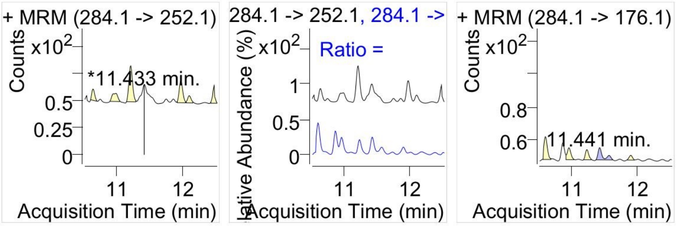

Emamectin benzoate

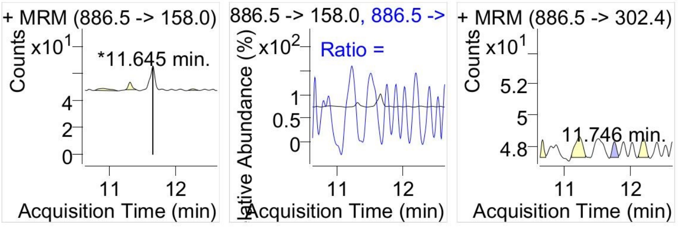

Azinphos-Ethyl

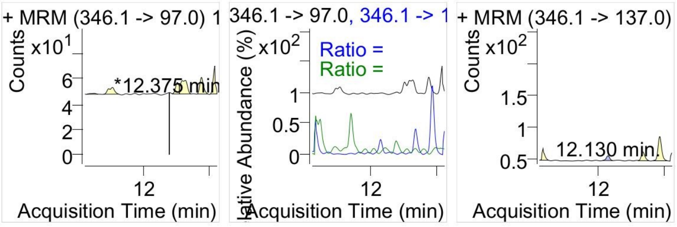

Tebufenozide

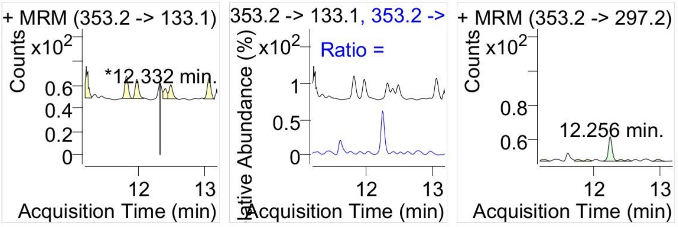

Chlorfenvinphos

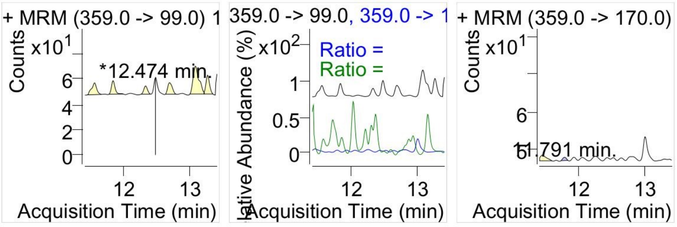

Aclonifen

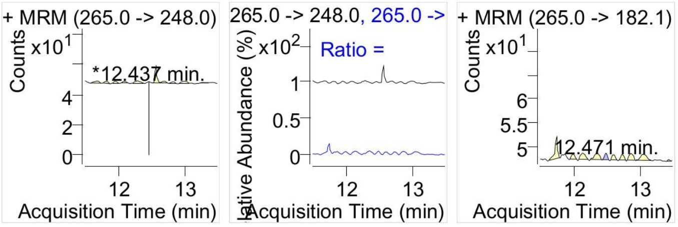

Pirimifos-methyl

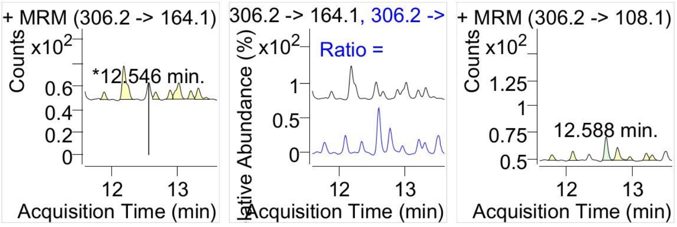

Kresoxim methyl

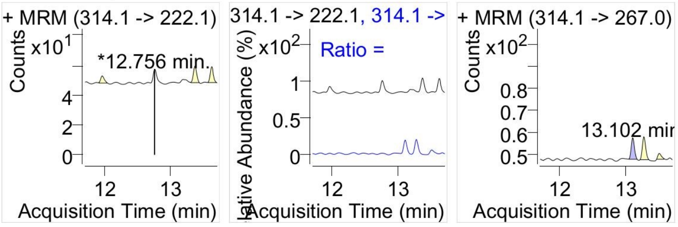

Difenoconazole

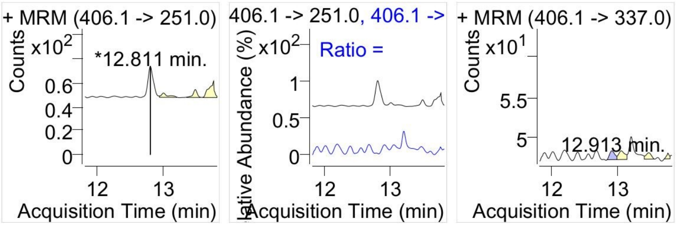

Fluopyram

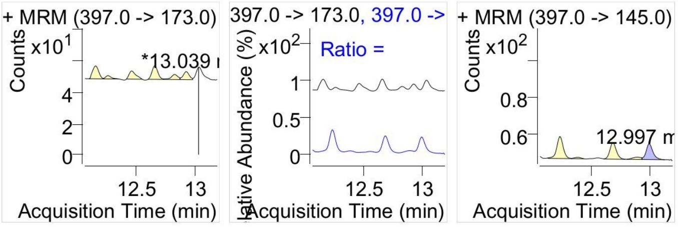

Trifloxystrobin

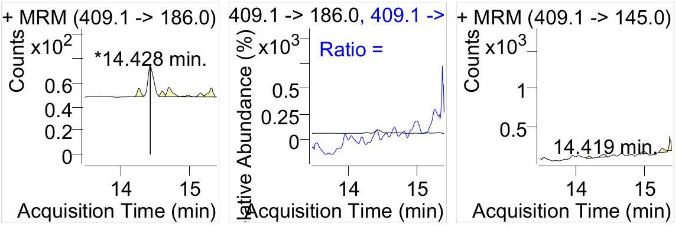

Indoxacarb

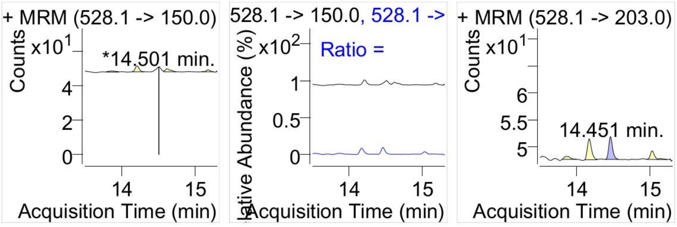

Propaquizafop

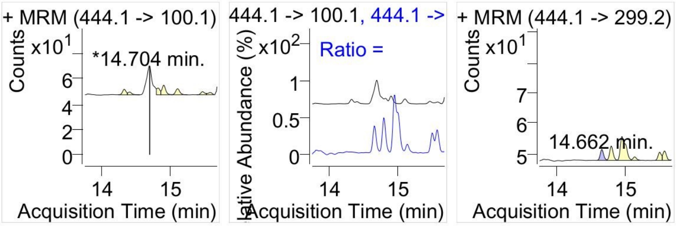

Allethrin

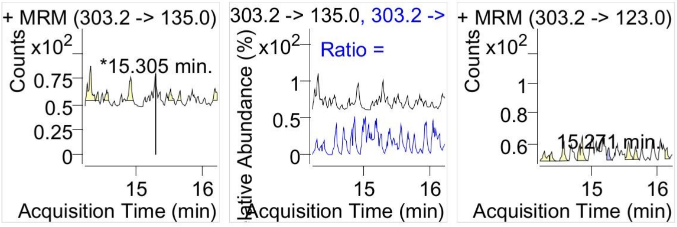

Pyriproxyfen

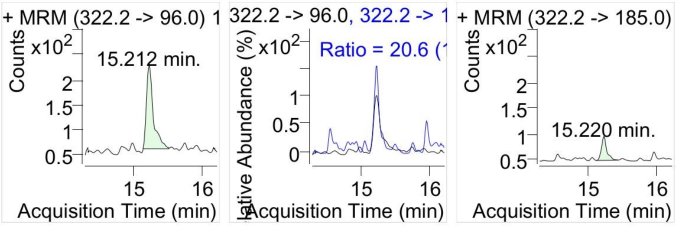

Fluazinam

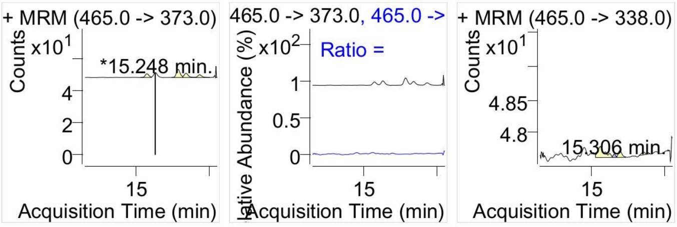

Chlorpyrifos

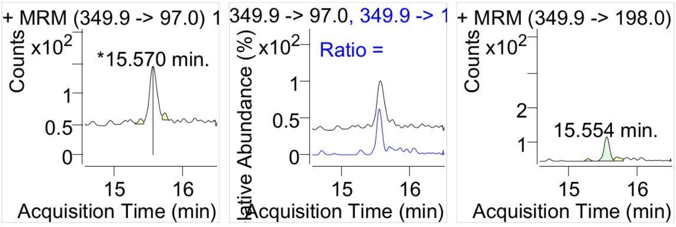

By Sample Quant Report

Analysis Info

**Instrument**	LCMS	**Operator**	
**Data File**	1362-PES2-24.d	**Sample Name**	1362-PES2-24
**Sample Type**	Sample	**Dilution**	0.003
**Acq. Method**	Pesticides_MRM_EN-15662_2024-06-27.m	**Acq. Date**	9/13/2024 9:58:59 AM
**Position**	P1-C1		-1

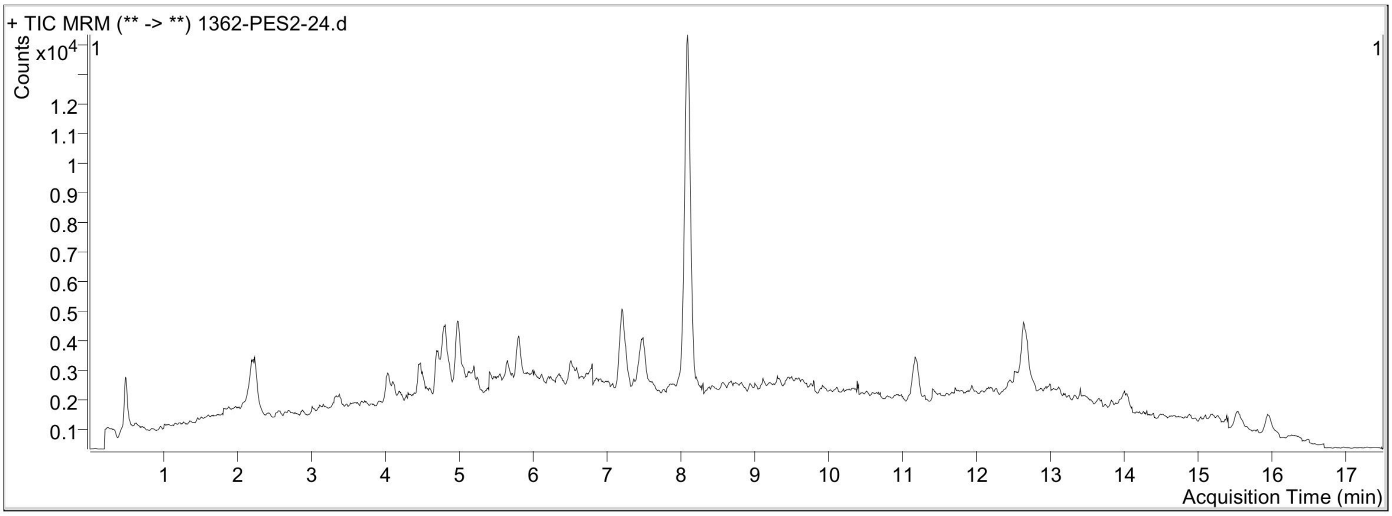

Quantitation Results

Compound	RT	Ref RT	Transition(T)	Transition(Q)	T-Resp	Q-Resp	QRatio	Ref QRatio	Final Conc.	Units
Methamidophos	0.49	0.76	142.0 -> 93.9	142.0 -> 124.9	0	1152		31.6	0.00	ng/ml
Acephate	0.93	0.99	184.0 -> 94.6	184.0 -> 95.0	0	41		69.3	0.00	ng/ml
Carbendazim	2.21	2.31	192.1 -> 160.1	192.1 -> 132.1	0	2604		16.8	0.00	ng/ml
Methomyl	3.37	3.34	163.1 -> 88.0	163.1 -> 106.0	0	82		64.3	0.00	ng/ml
Monocrotophos	3.98	3.85	224.1 -> 127.0	224.1 -> 58.0	0	96		51.6	0.00	ng/ml
Thiamethoxam	4.10	4.19	292.0 -> 211.1	292.0 -> 181.1	0	500		47.7	0.00	ng/ml
Clothianidin	4.50	4.59	250.0 -> 169.0	250.0 -> 131.9	0	258		77.4	0.00	ng/ml
Imidacloprid	4.70	4.75	256.0 -> 175.0	256.0 -> 208.9	4158	2459	59.2	86.8	0.06	ng/ml
Dimethoate	4.78	4.85	230.0 -> 198.8	230.0 -> 125.0	2460	2180	88.6	99.7	0.03	ng/ml
Acetamiprid	4.98	5.02	223.1 -> 126.0	223.1 -> 56.0	4559	2560	56.1	45.9	0.02	ng/ml
Sulfoxaflor	4.77	5.72	278.0 -> 174.0	278.0 -> 154.0	0	54		44.5	0.00	ng/ml
Amicarbazone	5.93	5.86	242.2 -> 143.1	242.2 -> 54.9	0			4.0	0.00	ng/ml
Ametryn	5.95	5.95	228.1 -> 186.1	228.1 -> 91.1	0	69		22.9	0.00	ng/ml
Bensulfuron-methyl	6.57	6.17	411.1 -> 182.1	411.1 -> 149.1	0	865		36.8	0.00	ng/ml
Nicosulfuron	6.07	6.17	411.1 -> 182.0	411.1 -> 181.9	0	9		100.0	0.00	ng/ml
Pyrimethanil	6.62	6.44	200.1 -> 82.0	200.1 -> 106.9	0	129		90.0	0.00	ng/ml
Terbutryn	7.20	7.23	242.1 -> 186.1	242.1 -> 68.1	0	3624		31.4	0.00	ng/ml
Atrazine	7.48	7.53	216.1 -> 174.1	216.1 -> 68.0	6936	3257	47.0	61.0	0.00	ng/ml
Spiroxamine	8.07	8.10	298.3 -> 144.1	298.3 -> 100.1	0	97		56.0	0.00	ng/ml
Metalaxyl	8.09	8.14	280.2 -> 220.1	280.2 -> 160.1	36968	28713	77.7	85.4	0.12	ng/ml
Triadimenol	9.62	9.69	296.1 -> 70.0	296.1 -> 99.1	0	86		4.6	0.00	ng/ml
Tebuconazole	9.79	9.81	308.1 -> 70.0	308.1 -> 124.9	0			1.1	0.00	ng/ml
Prochloraz	9.83	9.82	376.0 -> 308.0	376.0 -> 265.9	0	23		12.8	0.00	ng/ml
Dimethomorph	9.81	9.85	388.1 -> 165.1	388.1 -> 301.1	0	22		96.7	0.00	ng/ml
Promecarb	10.40	10.24	208.1 -> 109.1	208.1 -> 151.1	0	39		95.6	0.00	ng/ml
Triadimefon	10.83	10.85	294.1 -> 197.2	294.1 -> 225.1	0			1.0	0.00	ng/ml
Boscalid	10.97	10.93	343.0 -> 307.1	343.0 -> 271.2	0	41		50.8	0.00	ng/ml
Metolachlor	11.55	11.53	284.1 -> 252.1	284.1 -> 176.1	0	47		49.4	0.00	ng/ml
Emamectin benzoate	11.59	11.62	886.5 -> 158.0	886.5 -> 302.4	0			1.3	0.00	ng/ml
Azinphos-Ethyl	12.20	12.10	346.1 -> 97.0	346.1 -> 137.0	0	73		69.7	0.00	ng/ml
Tebufenozide	12.26	12.20	353.2 -> 133.1	353.2 -> 297.2	0	125		38.8	0.00	ng/ml
Chlorfenvinphos	12.41	12.41	359.0 -> 99.0	359.0 -> 170.0	0			56.0	0.00	ng/ml
Aclonifen	12.61	12.47	265.0 -> 248.0	265.0 -> 182.1	0	25		69.4	0.00	ng/ml
Pirimifos-methyl	12.53	12.58	306.2 -> 164.1	306.2 -> 108.1	0	536		56.3	0.00	ng/ml
Kresoxim methyl	12.61	12.71	314.1 -> 222.1	314.1 -> 267.0	0			92.4	0.00	ng/ml
Difenoconazole	12.76	12.82	406.1 -> 251.0	406.1 -> 337.0	0			12.9	0.00	ng/ml

By Sample Quant Report

Quantitation Results

Fluopyram	12.99	13.06	397.0 -> 173.0	397.0 -> 145.0	0	50		68.3	0.00	ng/ml
Trifloxystrobin	14.48	14.46	409.1 -> 186.0	409.1 -> 145.0	0			49.9	0.00	ng/ml
Indoxacarb	14.51	14.48	528.1 -> 150.0	528.1 -> 203.0	0	10		90.3	0.00	ng/ml
Propaquizafop	14.65	14.72	444.1 -> 100.1	444.1 -> 299.2	0	32		14.0	0.00	ng/ml
Allethrin	15.18	15.24	303.2 -> 135.0	303.2 -> 123.0	0	34		39.0	0.00	ng/ml
Pyriproxyfen	15.18	15.25	322.2 -> 96.0	322.2 -> 185.0	0	145		17.7	0.00	ng/ml
Fluazinam	15.31	15.29	465.0 -> 373.0	465.0 -> 338.0	0	2		13.5	0.00	ng/ml
Chlorpyrifos	15.53	15.58	349.9 -> 97.0	349.9 -> 198.0	0	1714		76.8	0.00	ng/ml

Compound Graphics

Methamidophos

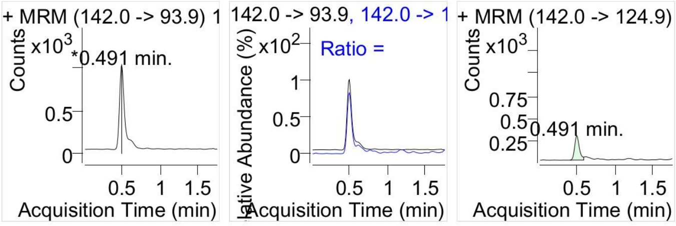

Acephate

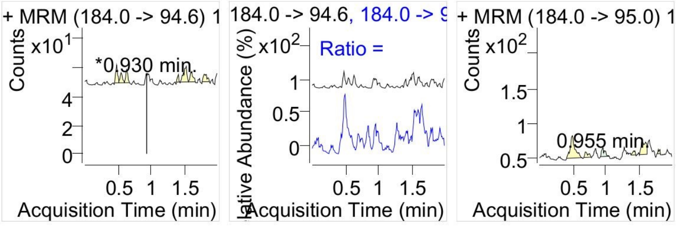

Carbendazim

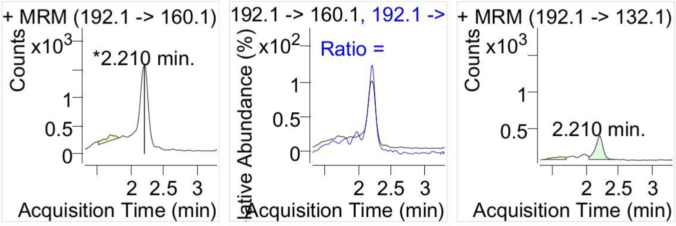

Methomyl

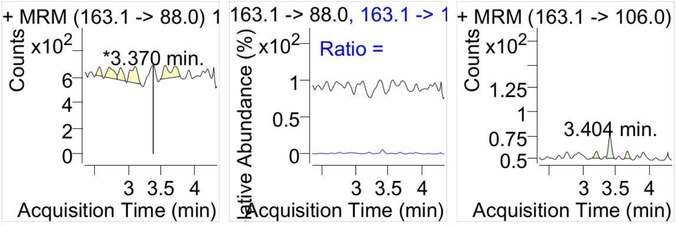

Monocrotophos

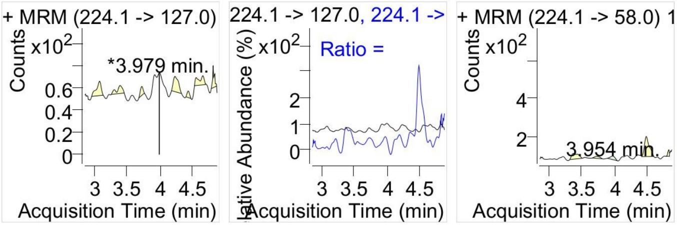

Thiamethoxam

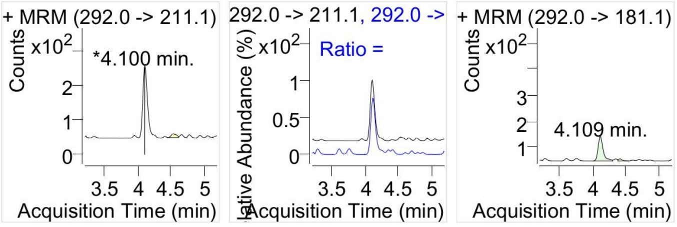

Clothianidin

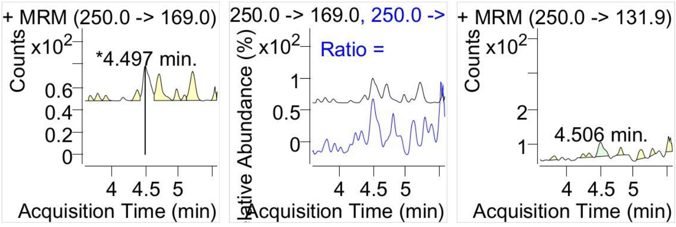

Imidacloprid

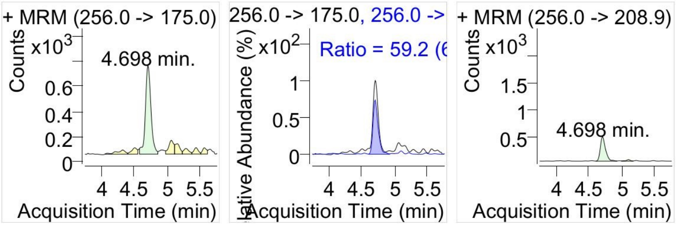

Dimethoate

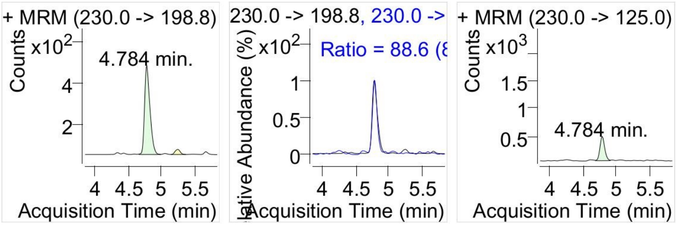

Acetamiprid

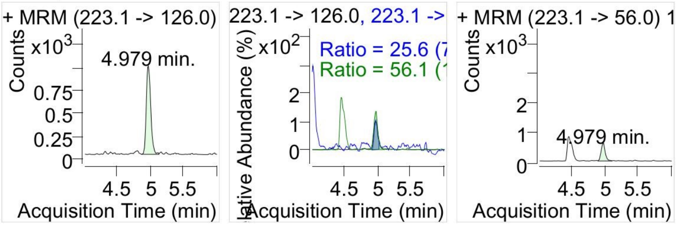

Sulfoxaflor

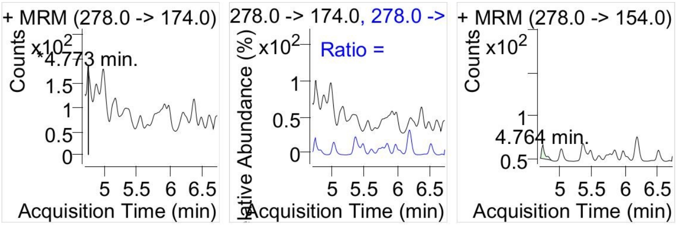

Amicarbazone

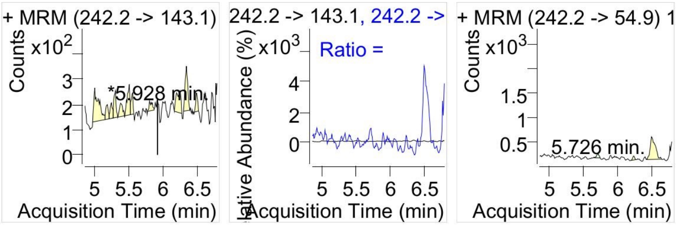

 Generated at 10:50 AM on 6/10/2025

By Sample Quant Report

Ametryn

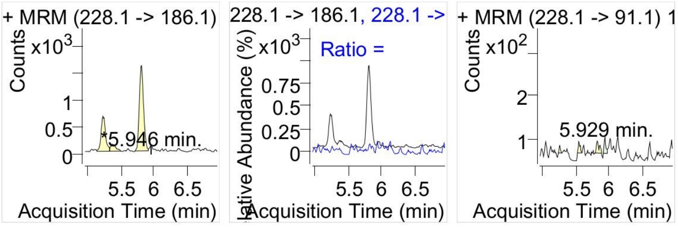

Bensulfuron-methyl

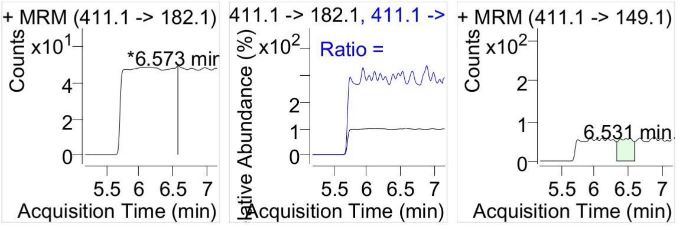

Nicosulfuron

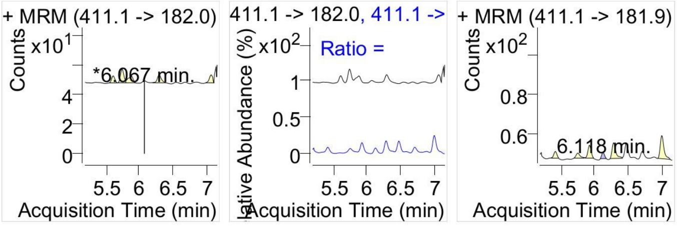

Pyrimethanil

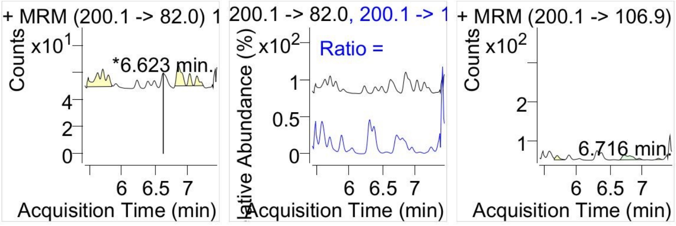

Terbutryn

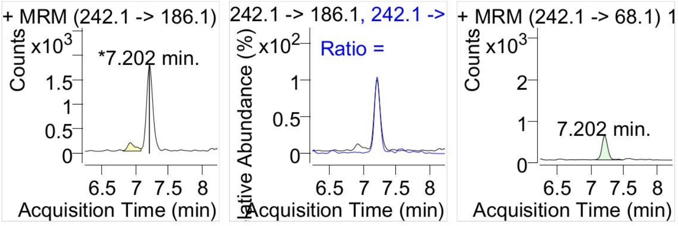

Atrazine

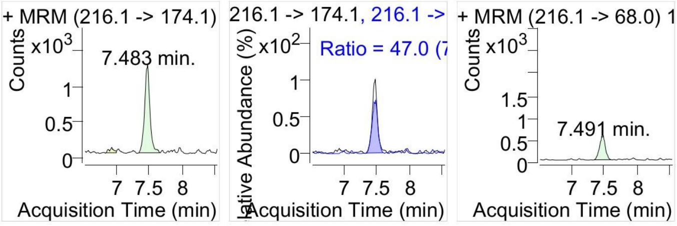

Spiroxamine

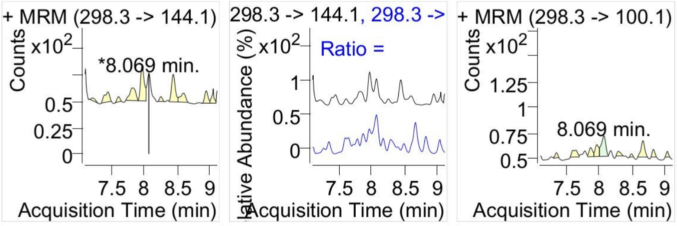

Metalaxyl

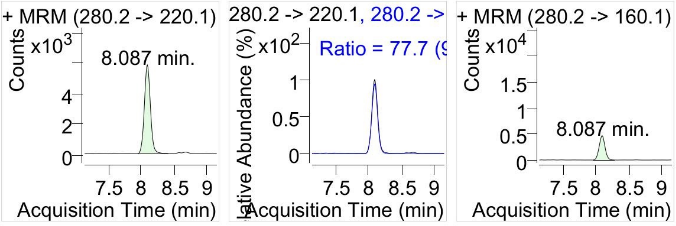

Triadimenol

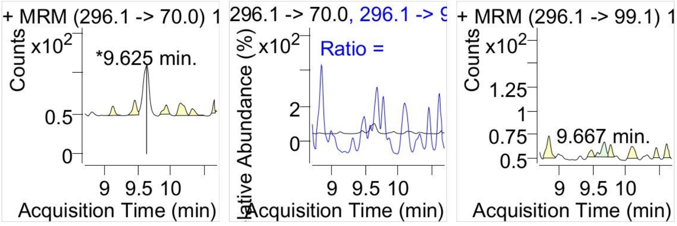

Tebuconazole

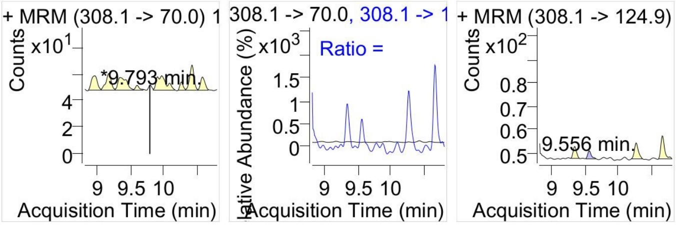

Prochloraz

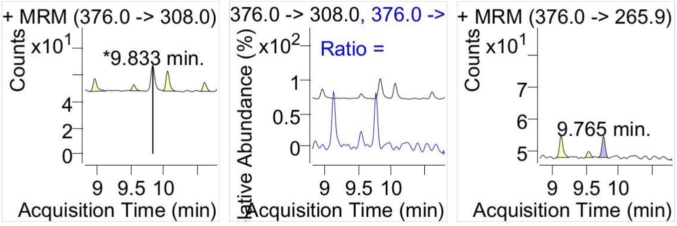

Dimethomorph

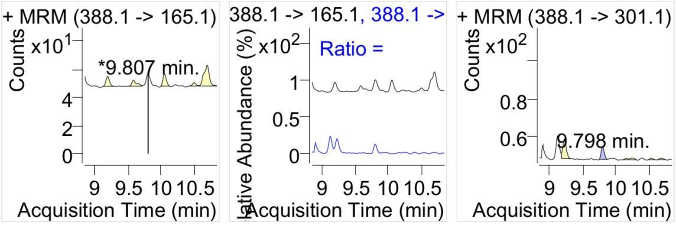

Promecarb

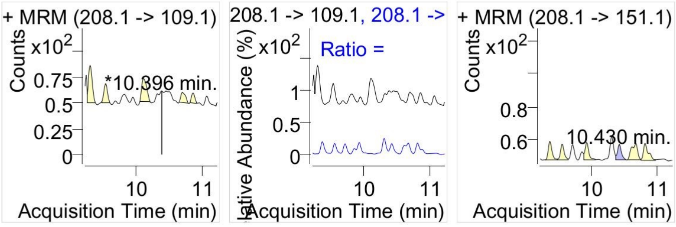

Triadimefon

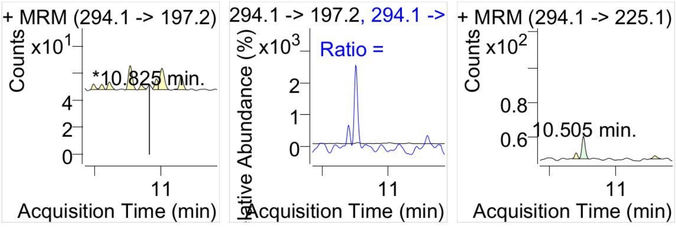

Boscalid

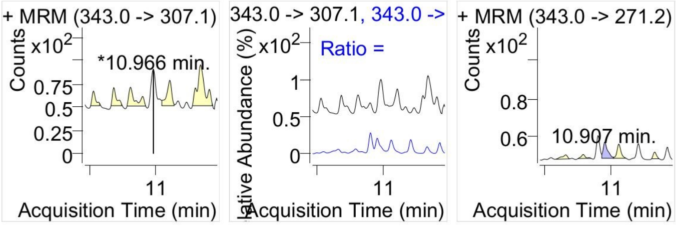

Metolachlor

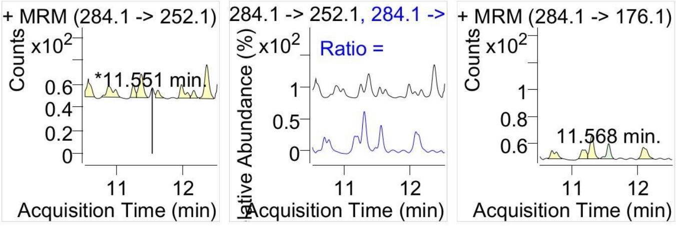

Emamectin benzoate

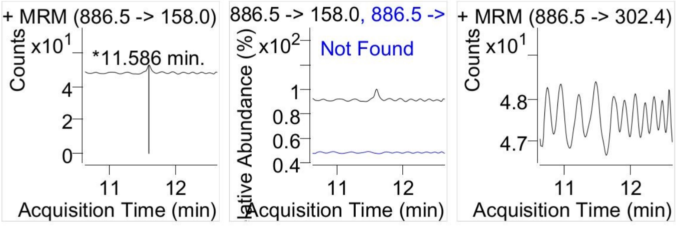

Azinphos-Ethyl

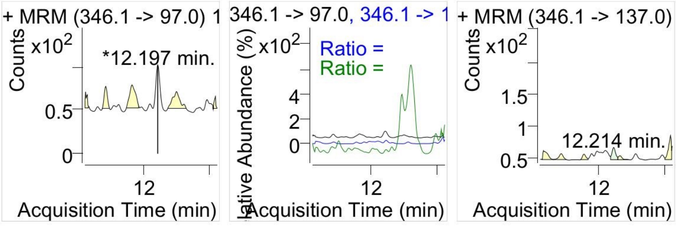

Tebufenozide

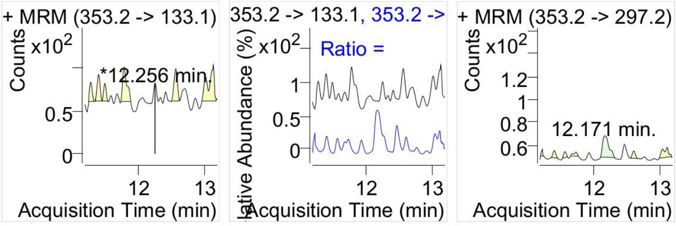

Chlorfenvinphos

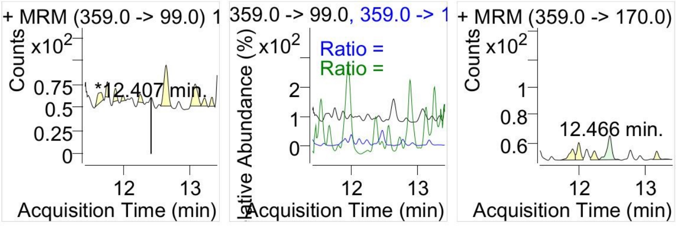

Aclonifen

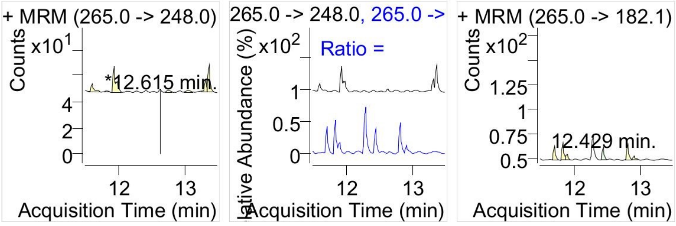

Pirimifos-methyl

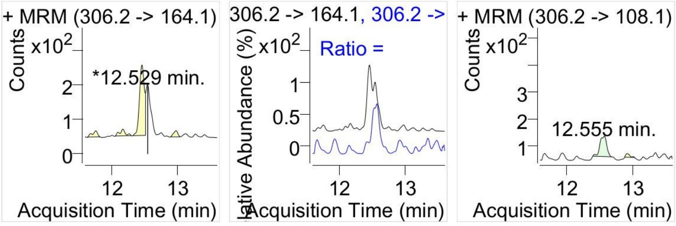

Kresoxim methyl

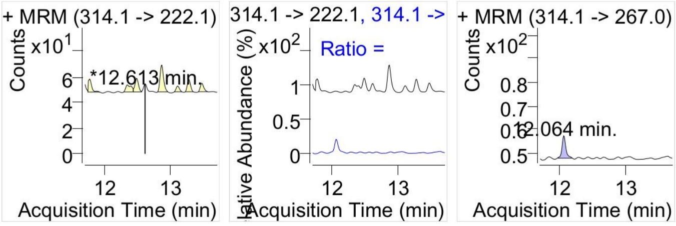

Difenoconazole

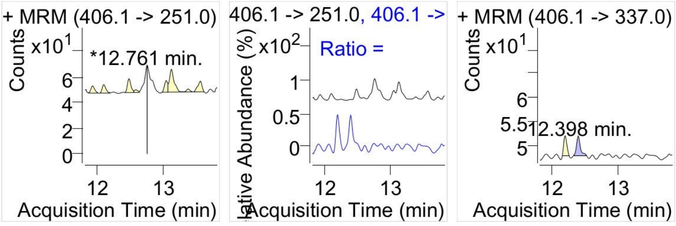

By Sample Quant Report

Fluopyram

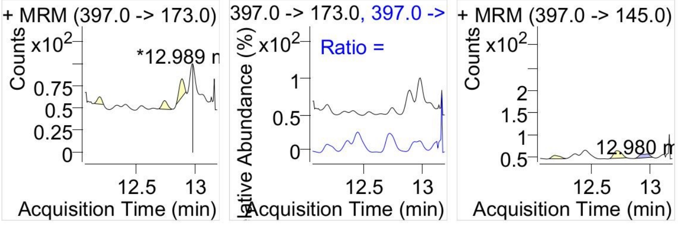

Trifloxystrobin

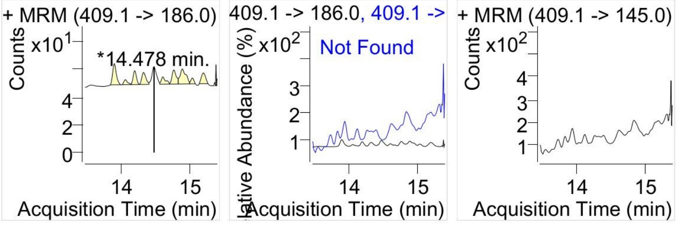

Indoxacarb

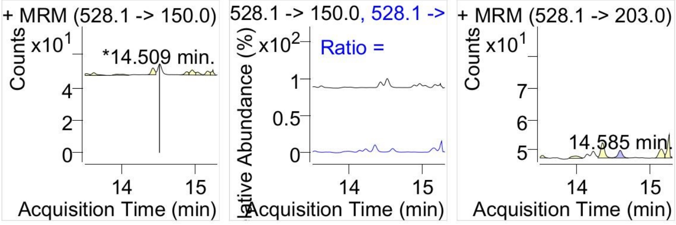

Propaquizafop

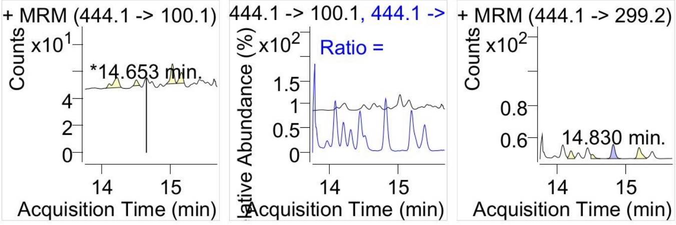

Allethrin

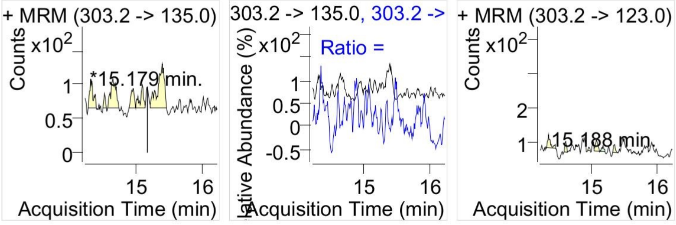

Pyriproxyfen

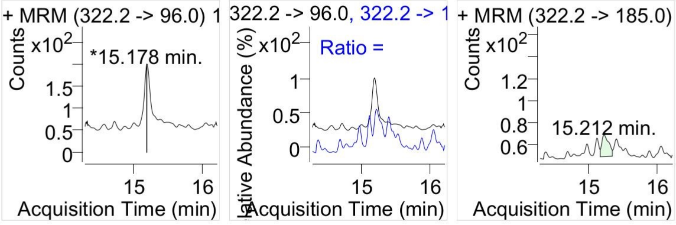

Fluazinam

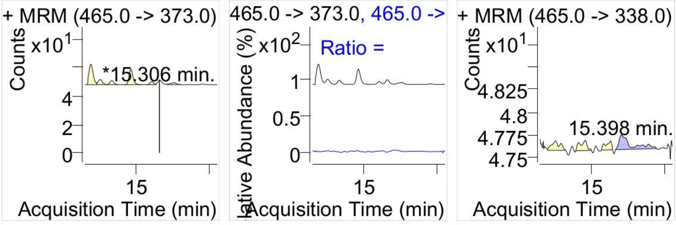

Chlorpyrifos

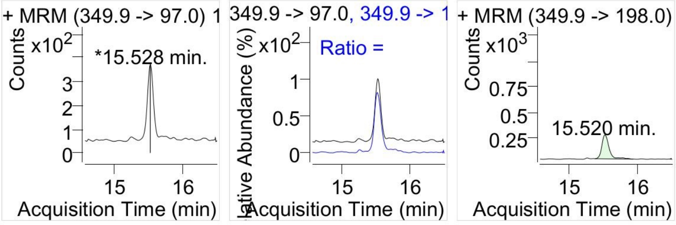

By Sample Quant Report

Analysis Info

**Instrument**	LCMS	**Operator**	
**Data File**	1363-PES2-24.d	**Sample Name**	1363-PES2-24
**Sample Type**	Sample	**Dilution**	0.003
**Acq. Method**	Pesticides_MRM_EN-15662_2024-06-27.m	**Acq. Date**	9/13/2024 10:21:50 AM
**Position**	P1-C2		-1

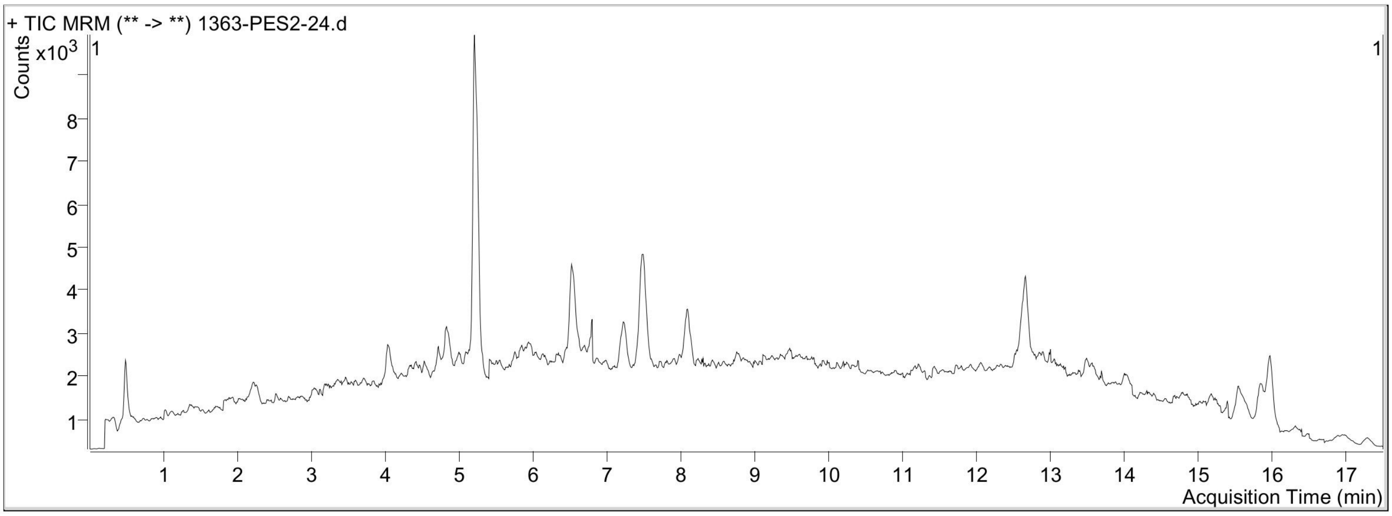

Quantitation Results

Compound	RT	Ref RT	Transition(T)	Transition(Q)	T-Resp	Q-Resp	QRatio	Ref QRatio	Final Conc.	Units
Methamidophos	0.49	0.76	142.0 -> 93.9	142.0 -> 124.9	0	626		31.6	0.00	ng/ml
Acephate	0.96	0.99	184.0 -> 94.6	184.0 -> 95.0	0	12		69.3	0.00	ng/ml
Carbendazim	2.23	2.31	192.1 -> 160.1	192.1 -> 132.1	0	389		16.8	0.00	ng/ml
Methomyl	3.46	3.34	163.1 -> 88.0	163.1 -> 106.0	0	48		64.3	0.00	ng/ml
Monocrotophos	3.73	3.85	224.1 -> 127.0	224.1 -> 58.0	0	20		51.6	0.00	ng/ml
Thiamethoxam	4.10	4.19	292.0 -> 211.1	292.0 -> 181.1	0	85		47.7	0.00	ng/ml
Clothianidin	4.72	4.59	250.0 -> 169.0	250.0 -> 131.9	0			77.4	0.00	ng/ml
Imidacloprid	4.71	4.75	256.0 -> 175.0	256.0 -> 208.9	0	576		86.8	0.00	ng/ml
Dimethoate	4.74	4.85	230.0 -> 198.8	230.0 -> 125.0	0	107		99.7	0.00	ng/ml
Acetamiprid	5.00	5.02	223.1 -> 126.0	223.1 -> 56.0	0	143		45.9	0.00	ng/ml
Sulfoxaflor	5.64	5.72	278.0 -> 174.0	278.0 -> 154.0	0			44.5	0.00	ng/ml
Amicarbazone	5.92	5.86	242.2 -> 143.1	242.2 -> 54.9	0			4.0	0.00	ng/ml
Ametryn	5.21	5.95	228.1 -> 186.1	228.1 -> 91.1	0	599		22.9	0.00	ng/ml
Bensulfuron-methyl	6.32	6.17	411.1 -> 182.1	411.1 -> 149.1	0	564		36.8	0.00	ng/ml
Nicosulfuron	6.19	6.17	411.1 -> 182.0	411.1 -> 181.9	0	47		100.0	0.00	ng/ml
Pyrimethanil	6.41	6.44	200.1 -> 82.0	200.1 -> 106.9	0	68		90.0	0.00	ng/ml
Terbutryn	7.23	7.23	242.1 -> 186.1	242.1 -> 68.1	0	1417		31.4	0.00	ng/ml
Atrazine	7.49	7.53	216.1 -> 174.1	216.1 -> 68.0	9612	5870	61.1	61.0	0.01	ng/ml
Spiroxamine	7.98	8.10	298.3 -> 144.1	298.3 -> 100.1	0	36		56.0	0.00	ng/ml
Metalaxyl	8.09	8.14	280.2 -> 220.1	280.2 -> 160.1	0	2586		85.4	0.00	ng/ml
Triadimenol	9.70	9.69	296.1 -> 70.0	296.1 -> 99.1	0			4.6	0.00	ng/ml
Tebuconazole	9.87	9.81	308.1 -> 70.0	308.1 -> 124.9	0			1.1	0.00	ng/ml
Prochloraz	9.82	9.82	376.0 -> 308.0	376.0 -> 265.9	0			12.8	0.00	ng/ml
Dimethomorph	9.99	9.85	388.1 -> 165.1	388.1 -> 301.1	0	24		96.7	0.00	ng/ml
Promecarb	10.13	10.24	208.1 -> 109.1	208.1 -> 151.1	0			95.6	0.00	ng/ml
Triadimefon	10.87	10.85	294.1 -> 197.2	294.1 -> 225.1	0			1.0	0.00	ng/ml
Boscalid	10.79	10.93	343.0 -> 307.1	343.0 -> 271.2	0	37		50.8	0.00	ng/ml
Metolachlor	11.58	11.53	284.1 -> 252.1	284.1 -> 176.1	0			49.4	0.00	ng/ml
Emamectin benzoate	11.64	11.62	886.5 -> 158.0	886.5 -> 302.4	0			1.3	0.00	ng/ml
Azinphos-Ethyl	12.06	12.10	346.1 -> 97.0	346.1 -> 137.0	0			69.7	0.00	ng/ml
Tebufenozide	12.34	12.20	353.2 -> 133.1	353.2 -> 297.2	0	18		38.8	0.00	ng/ml
Chlorfenvinphos	12.44	12.41	359.0 -> 99.0	359.0 -> 170.0	0			56.0	0.00	ng/ml
Aclonifen	12.50	12.47	265.0 -> 248.0	265.0 -> 182.1	0			69.4	0.00	ng/ml
Pirimifos-methyl	12.55	12.58	306.2 -> 164.1	306.2 -> 108.1	0	304		56.3	0.00	ng/ml
Kresoxim methyl	12.94	12.71	314.1 -> 222.1	314.1 -> 267.0	0	7		92.4	0.00	ng/ml
Difenoconazole	12.82	12.82	406.1 -> 251.0	406.1 -> 337.0	0	82		12.9	0.00	ng/ml

Quantitation Results

Fluopyram	13.17	13.06	397.0 -> 173.0	397.0 -> 145.0	0			68.3	0.00	ng/ml
Trifloxystrobin	14.44	14.46	409.1 -> 186.0	409.1 -> 145.0	0	432		49.9	0.00	ng/ml
Indoxacarb	14.38	14.48	528.1 -> 150.0	528.1 -> 203.0	0	2		90.3	0.00	ng/ml
Propaquizafop	14.71	14.72	444.1 -> 100.1	444.1 -> 299.2	0	15		14.0	0.00	ng/ml
Allethrin	15.17	15.24	303.2 -> 135.0	303.2 -> 123.0	0	145		39.0	0.00	ng/ml
Pyriproxyfen	15.20	15.25	322.2 -> 96.0	322.2 -> 185.0	0	112		17.7	0.00	ng/ml
Fluazinam	15.27	15.29	465.0 -> 373.0	465.0 -> 338.0	0			13.5	0.00	ng/ml
Chlorpyrifos	15.55	15.58	349.9 -> 97.0	349.9 -> 198.0	0	1259		76.8	0.00	ng/ml

By Sample Quant Report

Compound Graphics

Methamidophos

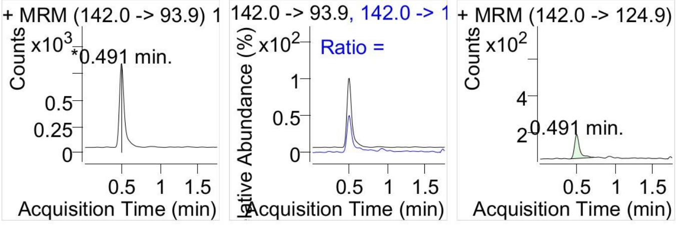

Acephate

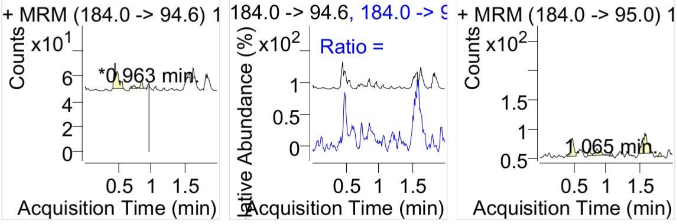

Carbendazim

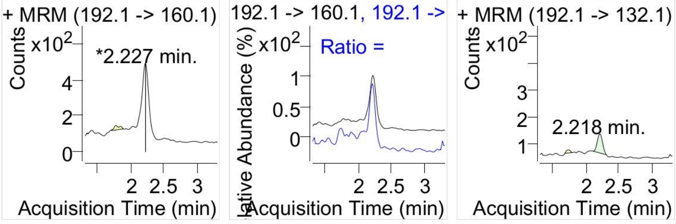

Methomyl

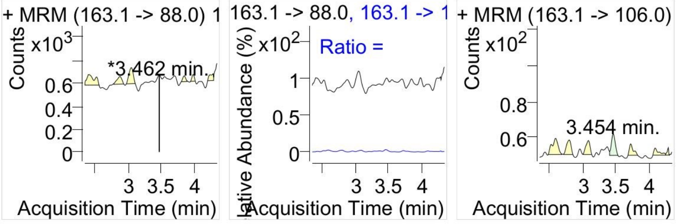

Monocrotophos

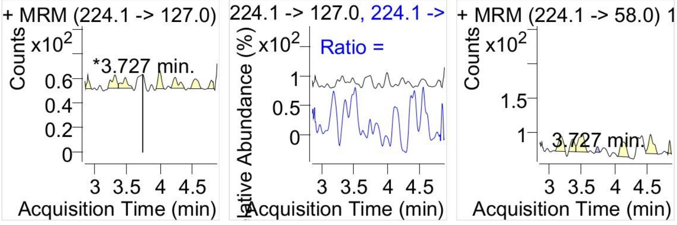

Thiamethoxam

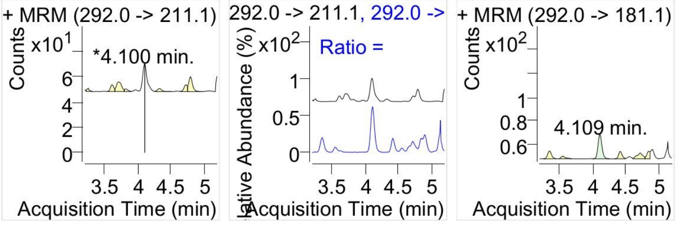

Clothianidin

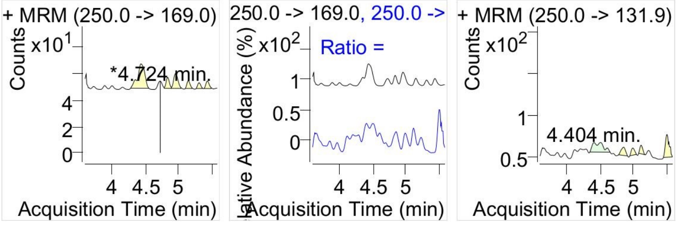

Imidacloprid

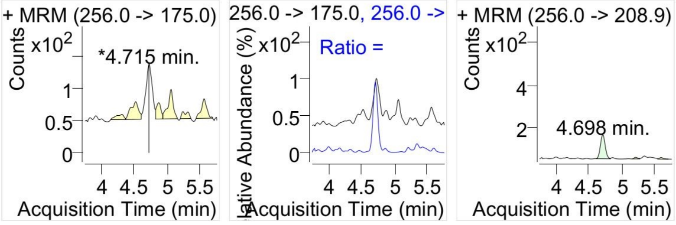

Dimethoate

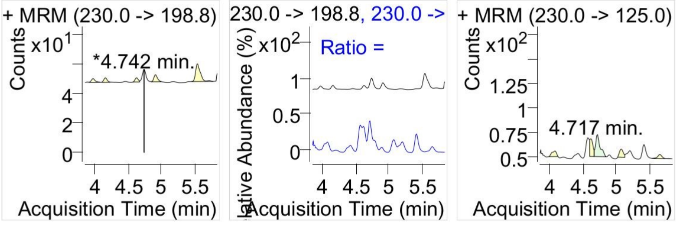

Acetamiprid

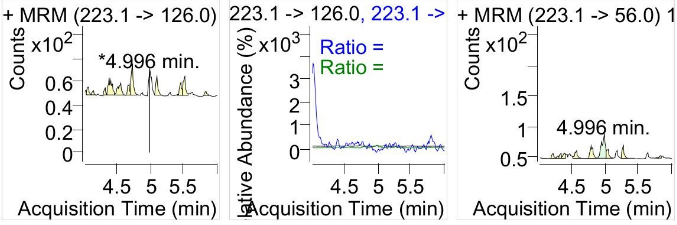

Sulfoxaflor

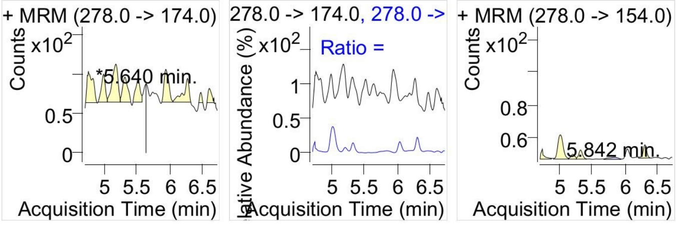

Amicarbazone

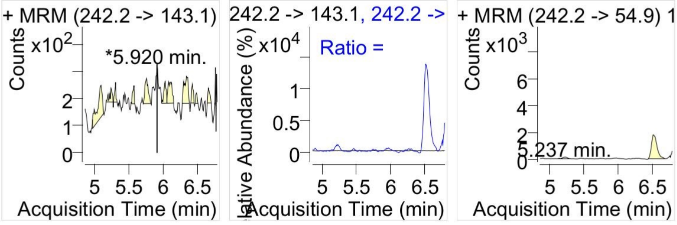

Ametryn

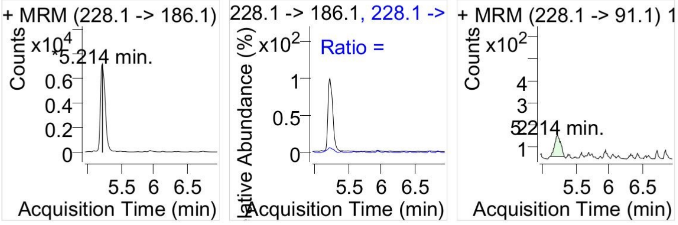

Bensulfuron-methyl

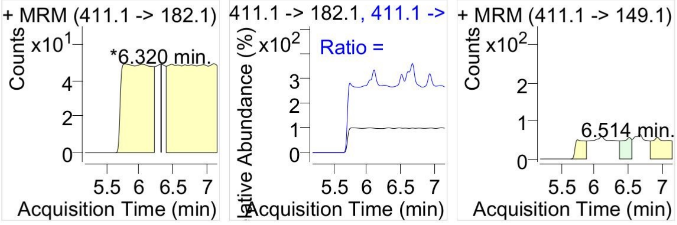

Nicosulfuron

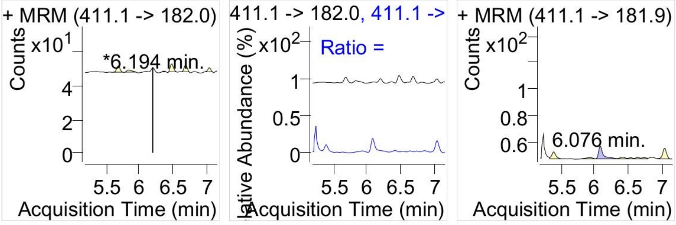

Pyrimethanil

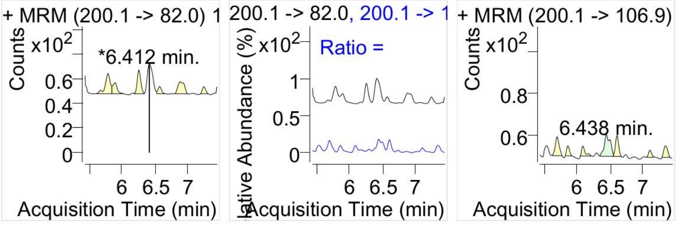

Terbutryn

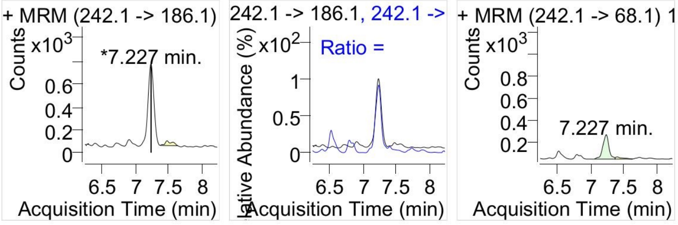

Atrazine

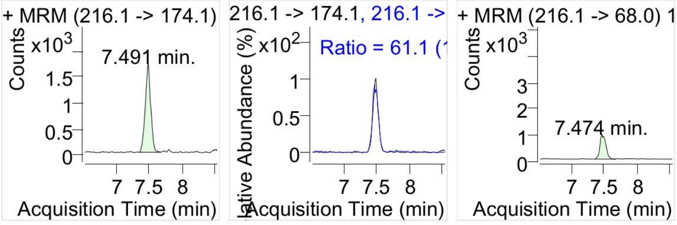

Spiroxamine

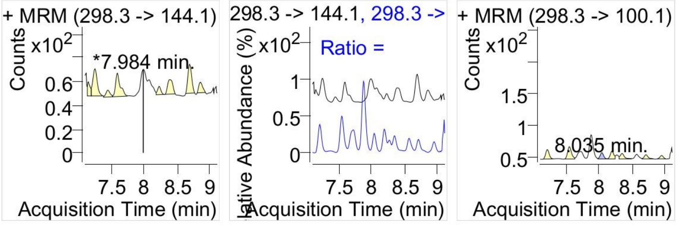

Metalaxyl

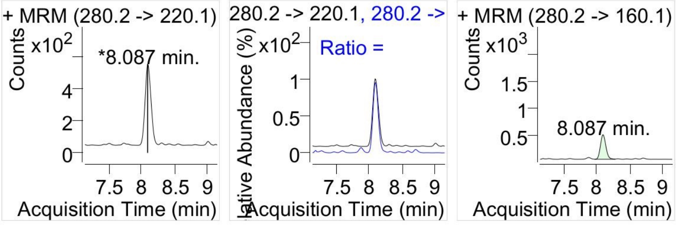

Triadimenol

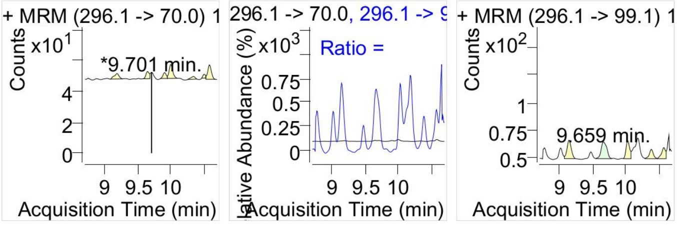

Tebuconazole

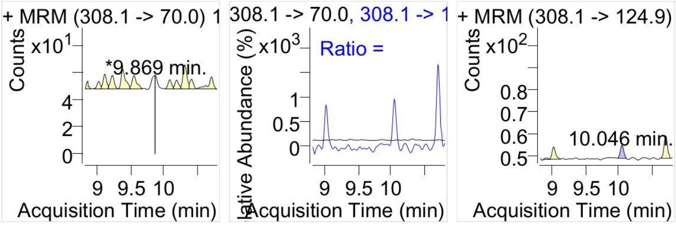

Prochloraz

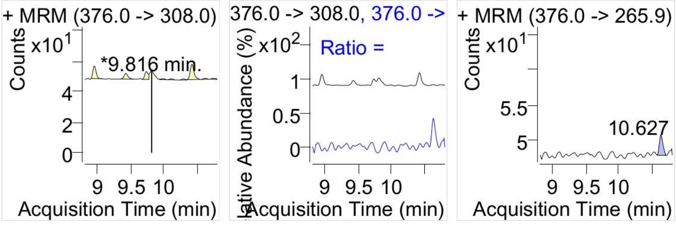

Dimethomorph

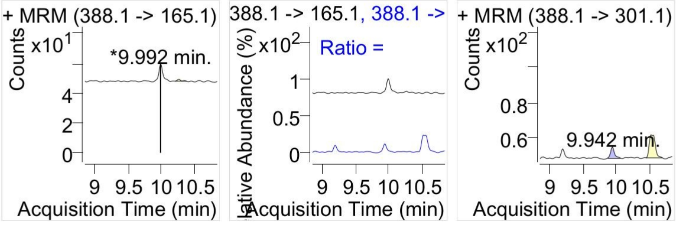

By Sample Quant Report

Promecarb

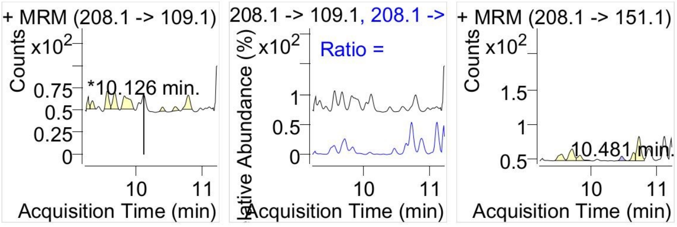

Triadimefon

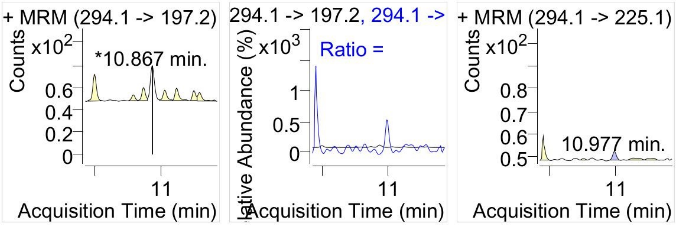

Boscalid

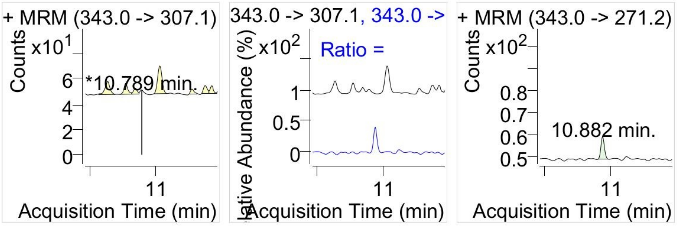

Metolachlor

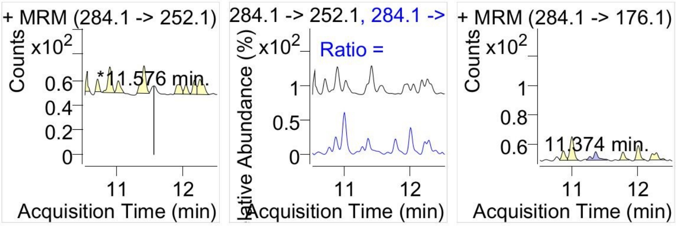

Emamectin benzoate

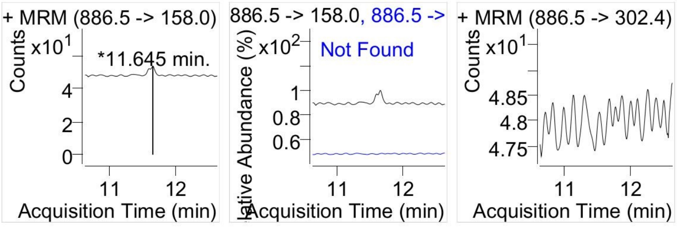

Azinphos-Ethyl

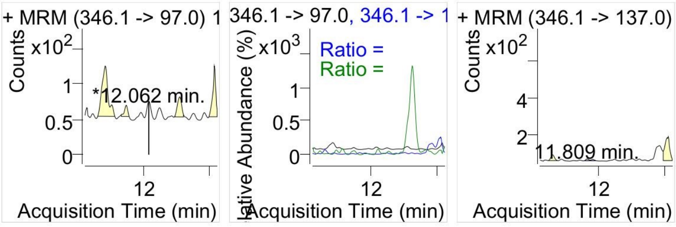

Tebufenozide

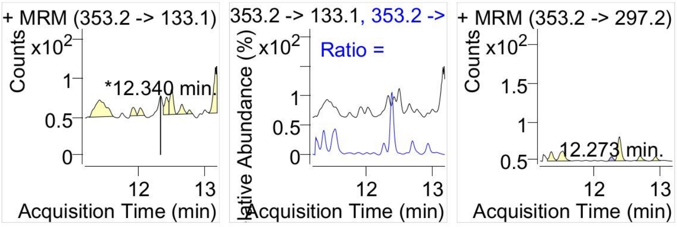

Chlorfenvinphos

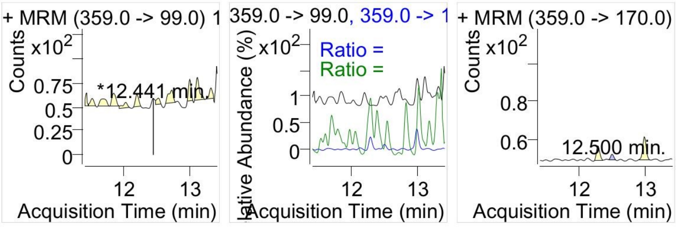

Aclonifen

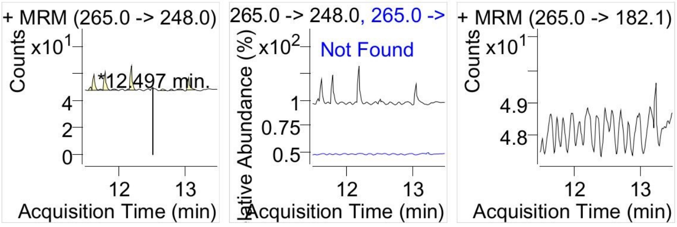

Pirimifos-methyl

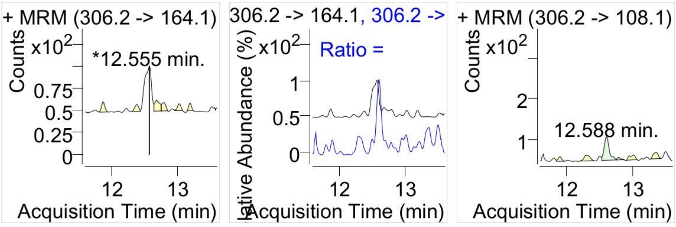

Kresoxim methyl

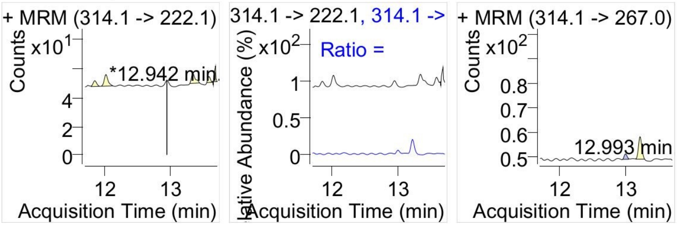

Difenoconazole

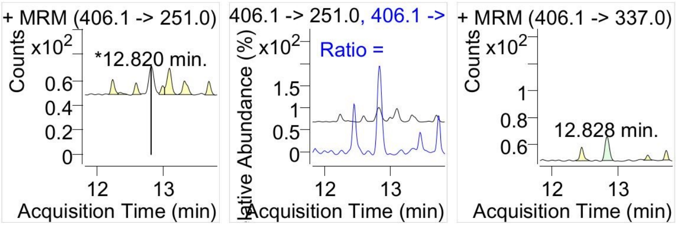

By Sample Quant Report

Fluopyram

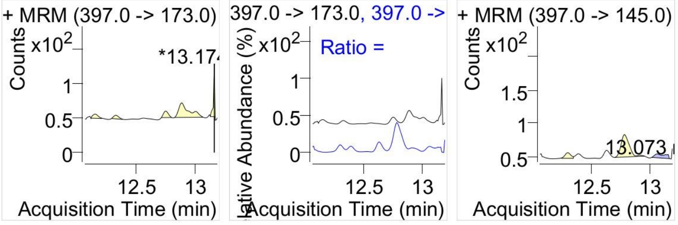

Trifloxystrobin

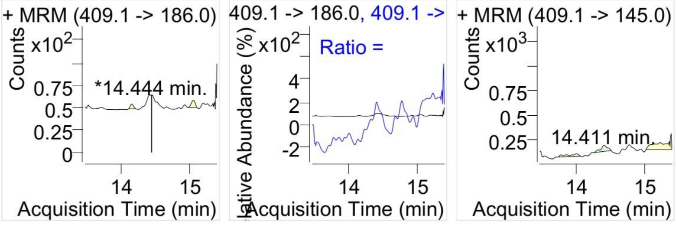

Indoxacarb

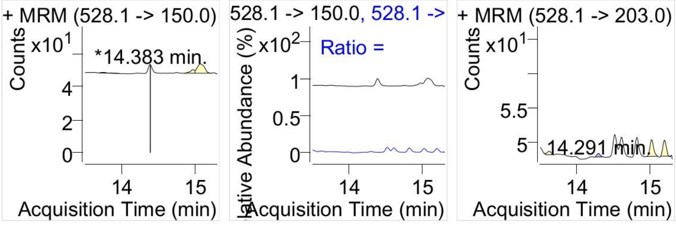

Propaquizafop

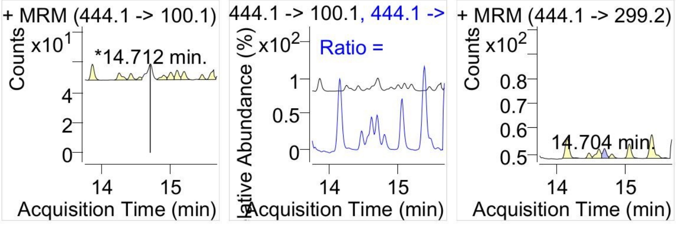

Allethrin

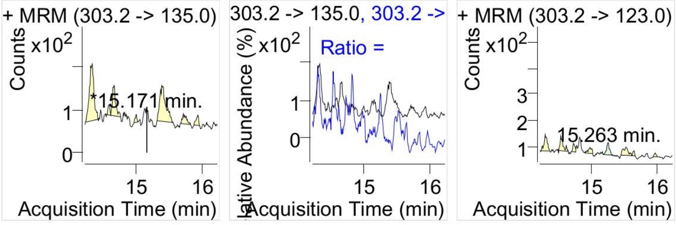

Pyriproxyfen

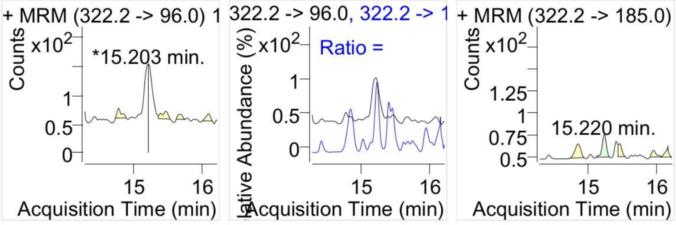

Fluazinam

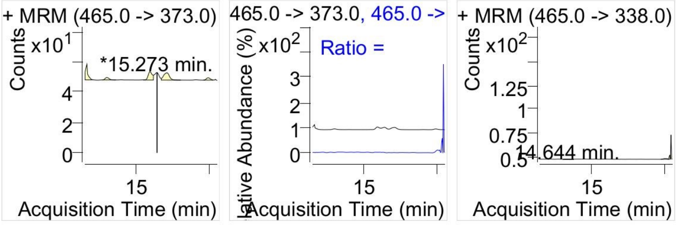

Chlorpyrifos

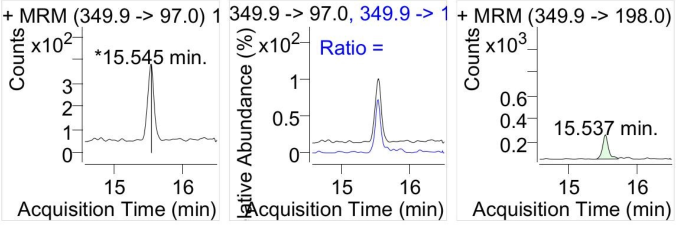

By Sample Quant Report

Analysis Info

**Instrument**	LCMS	**Operator**	
**Data File**	1364-PES2-24.d	**Sample Name**	1364-PES2-24
**Sample Type**	Sample	**Dilution**	0.003
**Acq. Method**	Pesticides_MRM_EN-15662_2024-06-27.m	**Acq. Date**	9/13/2024 10:44:41 AM
**Position**	P1-C3		-1

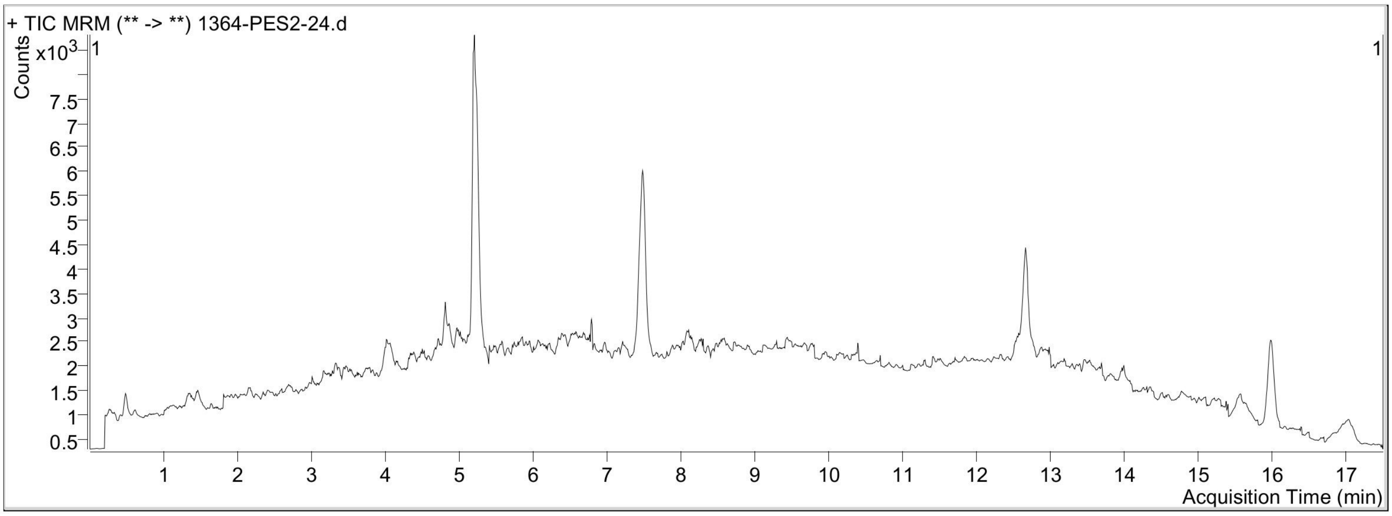

Quantitation Results

Compound	RT	Ref RT	Transition(T)	Transition(Q)	T-Resp	Q-Resp	QRatio	Ref QRatio	Final Conc.	Units
Methamidophos	0.48	0.76	142.0 -> 93.9	142.0 -> 124.9	0	357		31.6	0.00	ng/ml
Acephate	0.92	0.99	184.0 -> 94.6	184.0 -> 95.0	0	27		69.3	0.00	ng/ml
Carbendazim	2.18	2.31	192.1 -> 160.1	192.1 -> 132.1	0	67		16.8	0.00	ng/ml
Methomyl	3.31	3.34	163.1 -> 88.0	163.1 -> 106.0	0	51		64.3	0.00	ng/ml
Monocrotophos	4.29	3.85	224.1 -> 127.0	224.1 -> 58.0	0	36		51.6	0.00	ng/ml
Thiamethoxam	4.26	4.19	292.0 -> 211.1	292.0 -> 181.1	0	15		47.7	0.00	ng/ml
Clothianidin	4.48	4.59	250.0 -> 169.0	250.0 -> 131.9	0	67		77.4	0.00	ng/ml
Imidacloprid	4.71	4.75	256.0 -> 175.0	256.0 -> 208.9	0	102		86.8	0.00	ng/ml
Dimethoate	4.85	4.85	230.0 -> 198.8	230.0 -> 125.0	0			99.7	0.00	ng/ml
Acetamiprid	4.97	5.02	223.1 -> 126.0	223.1 -> 56.0	0	192		45.9	0.00	ng/ml
Sulfoxaflor	5.80	5.72	278.0 -> 174.0	278.0 -> 154.0	0	15		44.5	0.00	ng/ml
Amicarbazone	5.84	5.86	242.2 -> 143.1	242.2 -> 54.9	0	273		4.0	0.00	ng/ml
Ametryn	5.20	5.95	228.1 -> 186.1	228.1 -> 91.1	0	281		22.9	0.00	ng/ml
Bensulfuron-methyl	6.11	6.17	411.1 -> 182.1	411.1 -> 149.1	0	750		36.8	0.00	ng/ml
Nicosulfuron	6.13	6.17	411.1 -> 182.0	411.1 -> 181.9	0	6		100.0	0.00	ng/ml
Pyrimethanil	6.42	6.44	200.1 -> 82.0	200.1 -> 106.9	0	63		90.0	0.00	ng/ml
Terbutryn	7.26	7.23	242.1 -> 186.1	242.1 -> 68.1	0	442		31.4	0.00	ng/ml
Atrazine	7.48	7.53	216.1 -> 174.1	216.1 -> 68.0	13498	9358	69.3	61.0	0.01	ng/ml
Spiroxamine	8.09	8.10	298.3 -> 144.1	298.3 -> 100.1	0	69		56.0	0.00	ng/ml
Metalaxyl	8.10	8.14	280.2 -> 220.1	280.2 -> 160.1	0	403		85.4	0.00	ng/ml
Triadimenol	9.66	9.69	296.1 -> 70.0	296.1 -> 99.1	0	7		4.6	0.00	ng/ml
Tebuconazole	9.85	9.81	308.1 -> 70.0	308.1 -> 124.9	0	15		1.1	0.00	ng/ml
Prochloraz	9.82	9.82	376.0 -> 308.0	376.0 -> 265.9	0	2		12.8	0.00	ng/ml
Dimethomorph	9.91	9.85	388.1 -> 165.1	388.1 -> 301.1	0			96.7	0.00	ng/ml
Promecarb	10.22	10.24	208.1 -> 109.1	208.1 -> 151.1	0			95.6	0.00	ng/ml
Triadimefon	10.83	10.85	294.1 -> 197.2	294.1 -> 225.1	0	13		1.0	0.00	ng/ml
Boscalid	10.95	10.93	343.0 -> 307.1	343.0 -> 271.2	0			50.8	0.00	ng/ml
Metolachlor	11.52	11.53	284.1 -> 252.1	284.1 -> 176.1	0	13		49.4	0.00	ng/ml
Emamectin benzoate		11.62	886.5 -> 158.0	886.5 -> 302.4				1.3	ND	ng/ml
Azinphos-Ethyl	12.35	12.10	346.1 -> 97.0	346.1 -> 137.0	0	125		69.7	0.00	ng/ml
Tebufenozide	12.37	12.20	353.2 -> 133.1	353.2 -> 297.2	0	17		38.8	0.00	ng/ml
Chlorfenvinphos	12.42	12.41	359.0 -> 99.0	359.0 -> 170.0	0	6		56.0	0.00	ng/ml
Aclonifen	12.15	12.47	265.0 -> 248.0	265.0 -> 182.1	0	3		69.4	0.00	ng/ml
Pirimifos-methyl	12.56	12.58	306.2 -> 164.1	306.2 -> 108.1	0	646		56.3	0.00	ng/ml
Kresoxim methyl	12.61	12.71	314.1 -> 222.1	314.1 -> 267.0	0	2		92.4	0.00	ng/ml
Difenoconazole	12.88	12.82	406.1 -> 251.0	406.1 -> 337.0	0	32		12.9	0.00	ng/ml

By Sample Quant Report

Quantitation Results

Fluopyram	13.18	13.06	397.0 -> 173.0	397.0 -> 145.0	0			68.3	0.00	ng/ml
Trifloxystrobin	14.46	14.46	409.1 -> 186.0	409.1 -> 145.0	0			49.9	0.00	ng/ml
Indoxacarb	14.46	14.48	528.1 -> 150.0	528.1 -> 203.0	0	27		90.3	0.00	ng/ml
Propaquizafop	14.76	14.72	444.1 -> 100.1	444.1 -> 299.2	0			14.0	0.00	ng/ml
Allethrin	15.42	15.24	303.2 -> 135.0	303.2 -> 123.0	0	94		39.0	0.00	ng/ml
Pyriproxyfen	15.24	15.25	322.2 -> 96.0	322.2 -> 185.0	0	67		17.7	0.00	ng/ml
Fluazinam	15.31	15.29	465.0 -> 373.0	465.0 -> 338.0	0			13.5	0.00	ng/ml
Chlorpyrifos	15.55	15.58	349.9 -> 97.0	349.9 -> 198.0	0	1416		76.8	0.00	ng/ml

Compound Graphics

Methamidophos

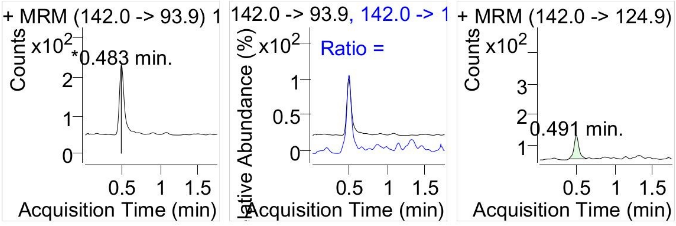

Acephate

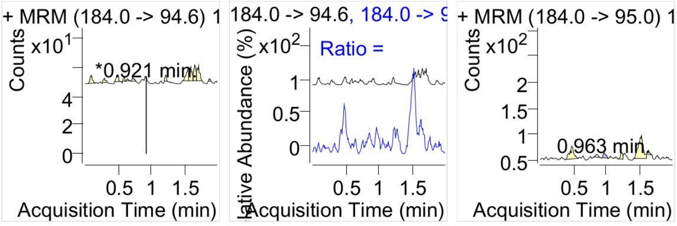

Carbendazim

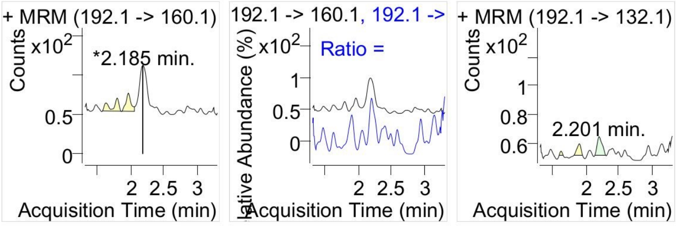

Methomyl

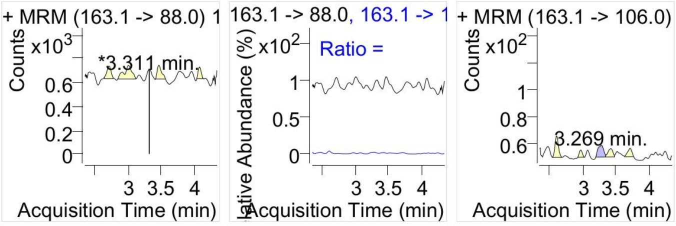

Monocrotophos

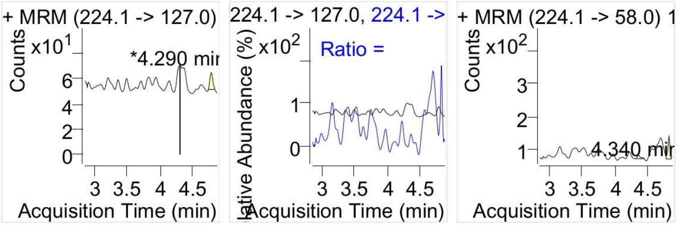

Thiamethoxam

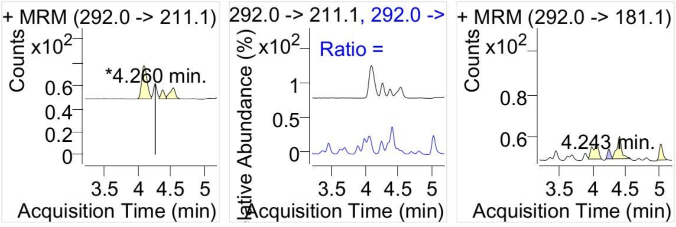

Clothianidin

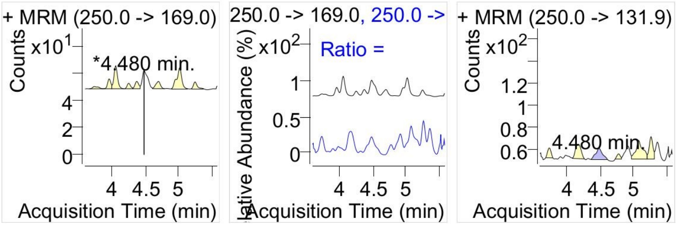

Imidacloprid

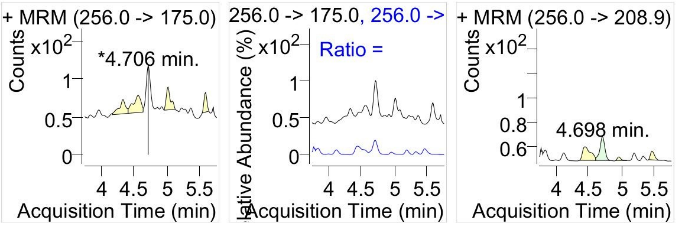

Dimethoate

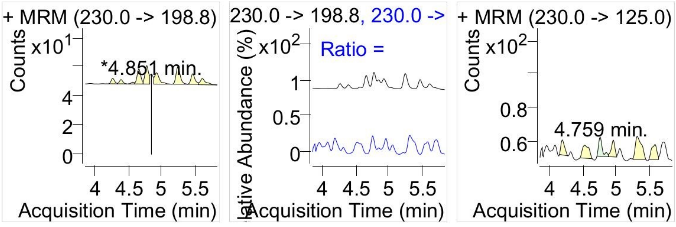

Acetamiprid

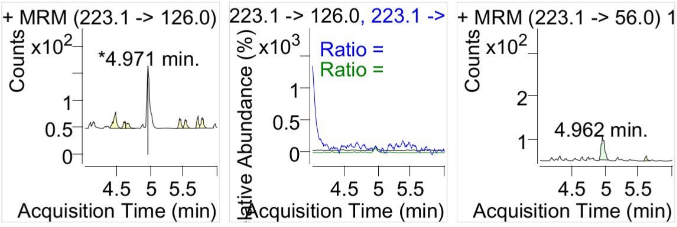

Sulfoxaflor

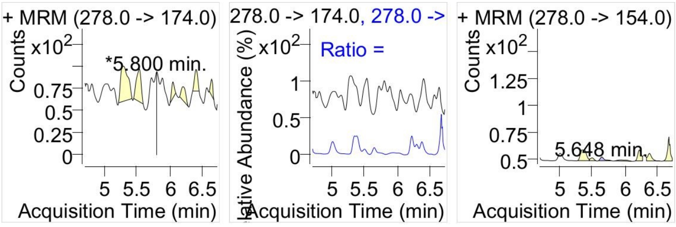

Amicarbazone

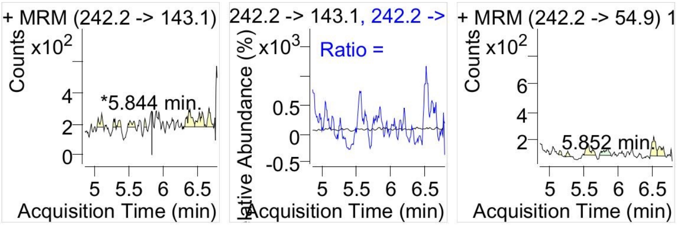

Ametryn

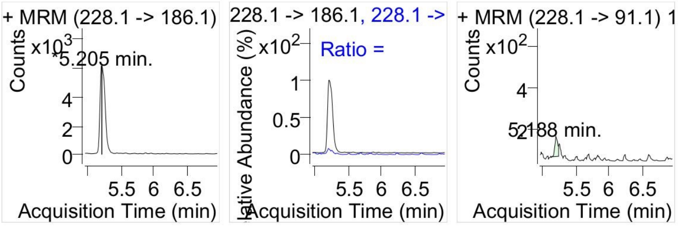

Bensulfuron-methyl

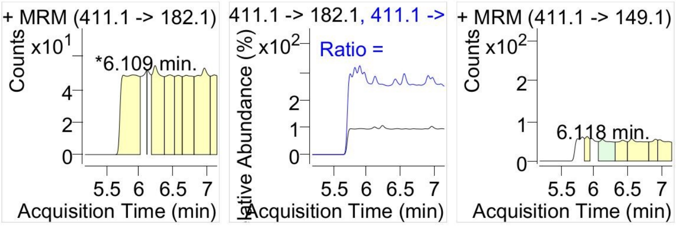

Nicosulfuron

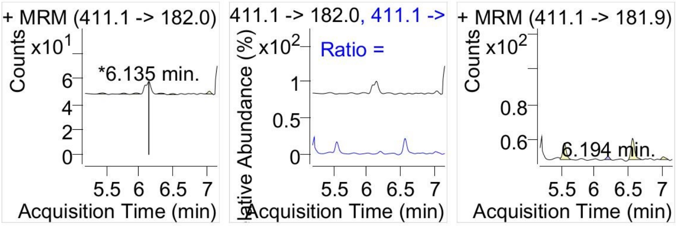

Pyrimethanil

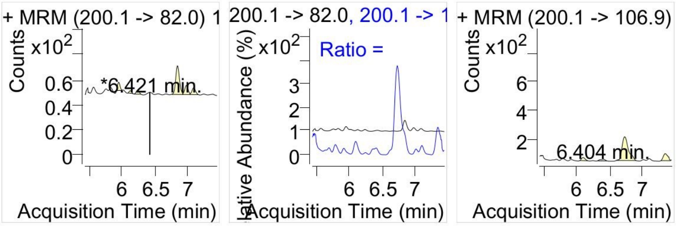

Terbutryn

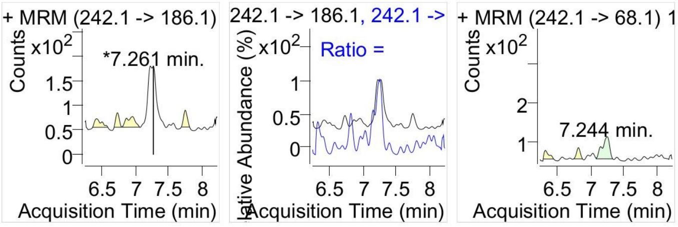

Atrazine

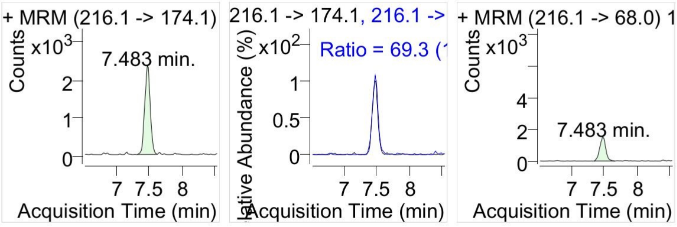

Spiroxamine

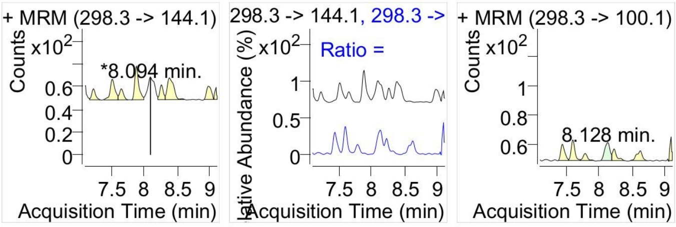

Metalaxyl

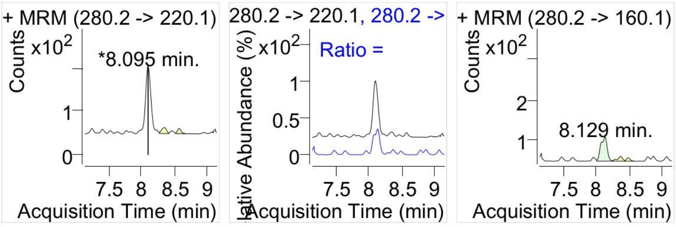

Triadimenol

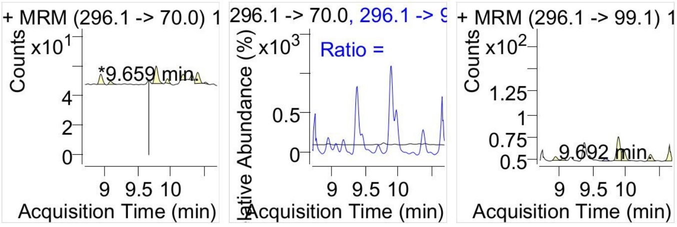

Tebuconazole

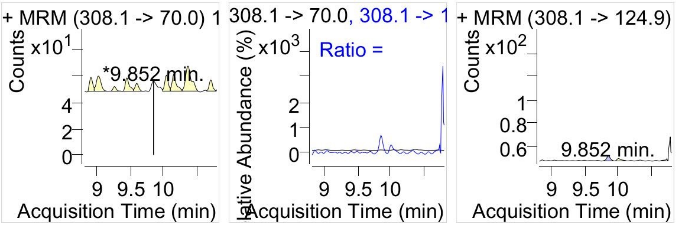

Prochloraz

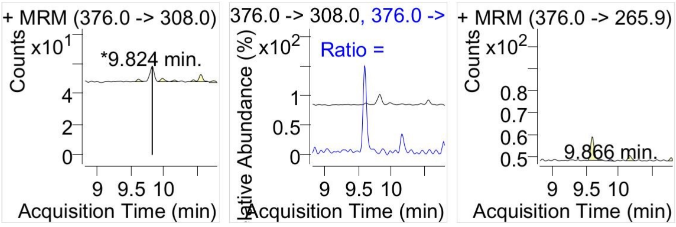

Dimethomorph

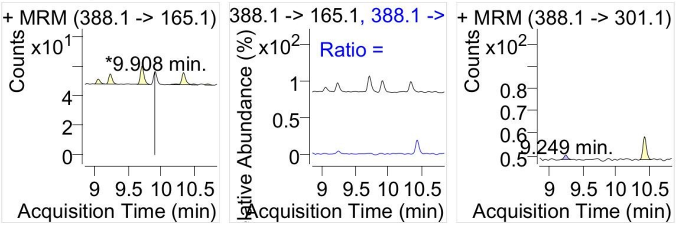

By Sample Quant Report

Promecarb

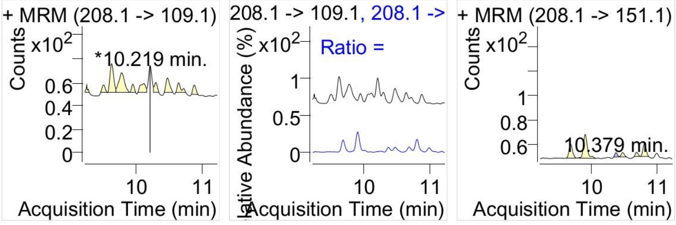

Triadimefon

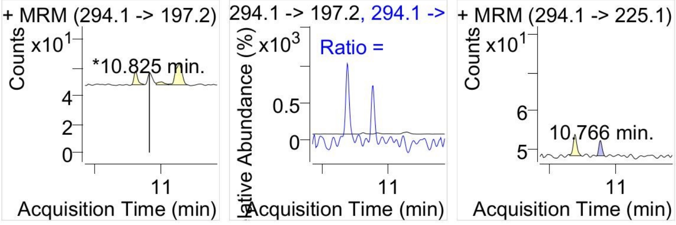

Boscalid

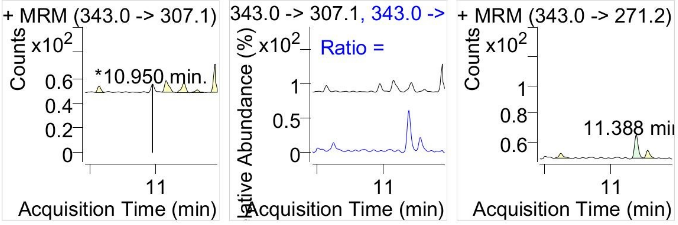

Metolachlor

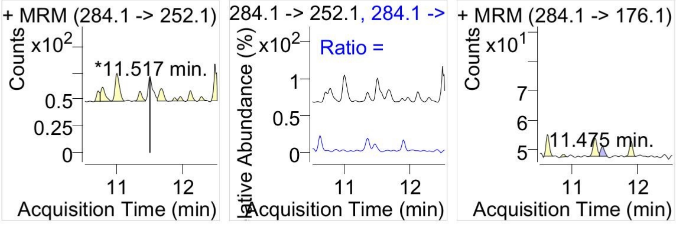

Emamectin benzoate

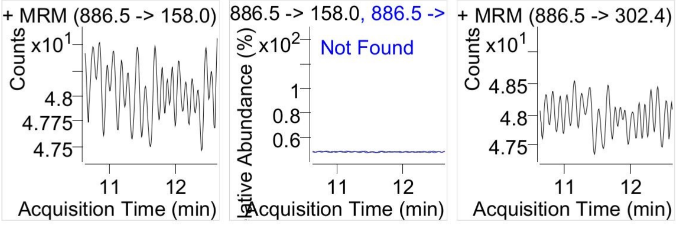

Azinphos-Ethyl

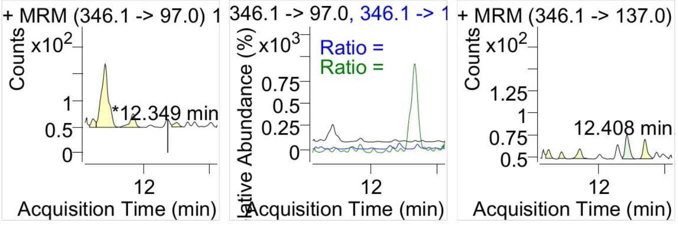

Tebufenozide

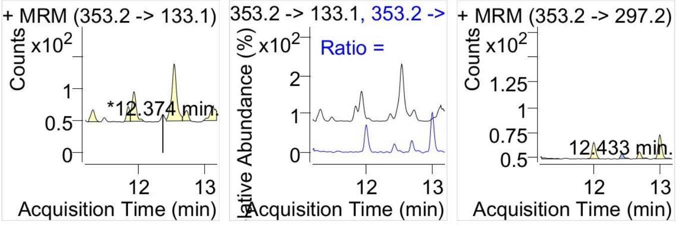

Chlorfenvinphos

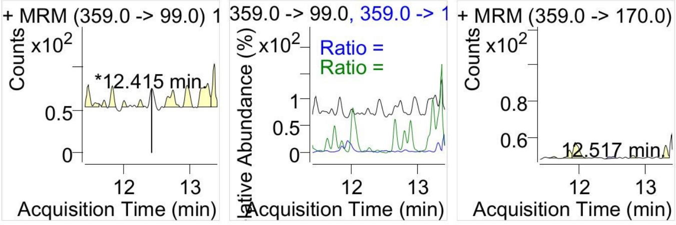

Aclonifen

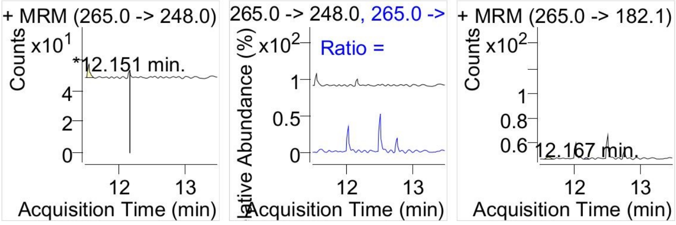

Pirimifos-methyl

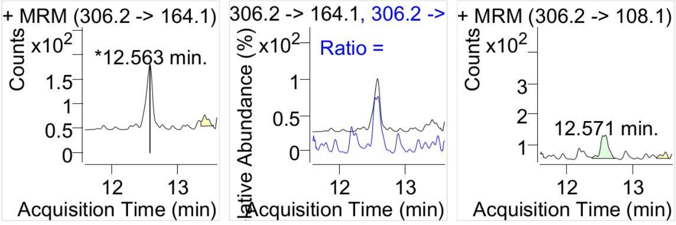

Kresoxim methyl

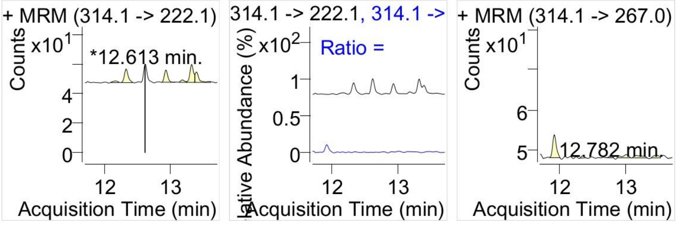

Difenoconazole

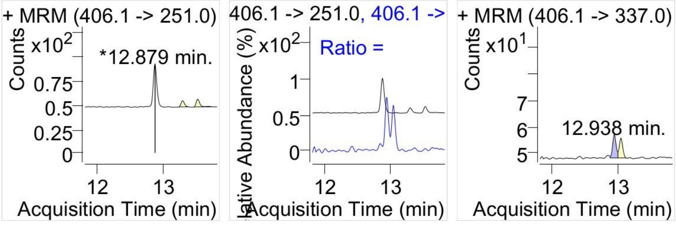

Fluopyram

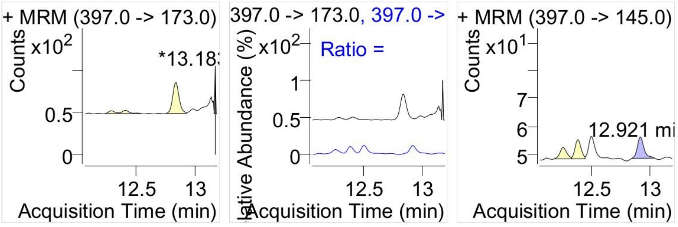

Trifloxystrobin

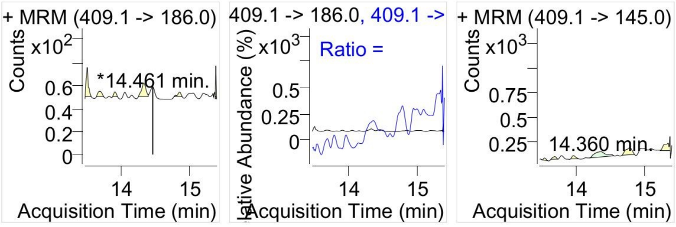

Indoxacarb

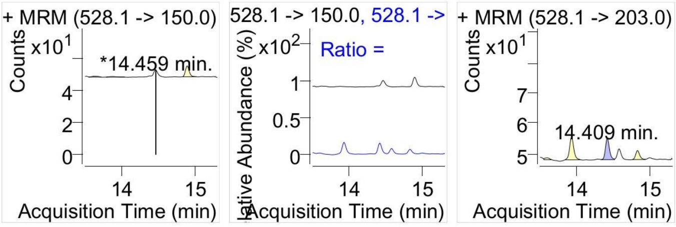

Propaquizafop

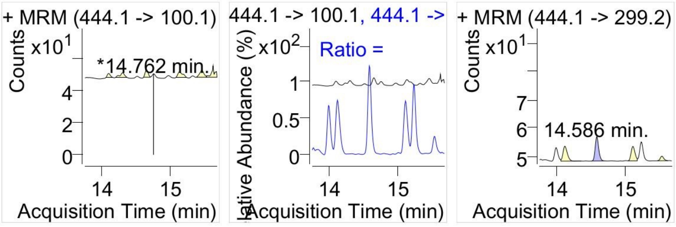

Allethrin

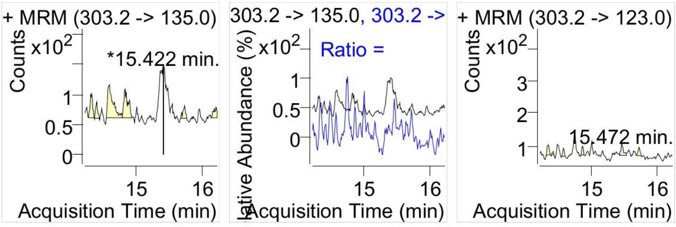

Pyriproxyfen

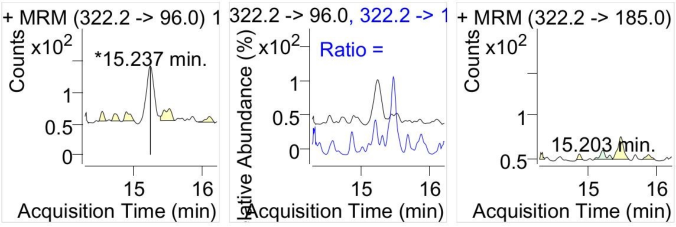

Fluazinam

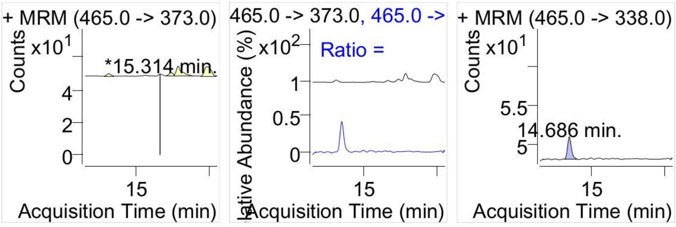

Chlorpyrifos

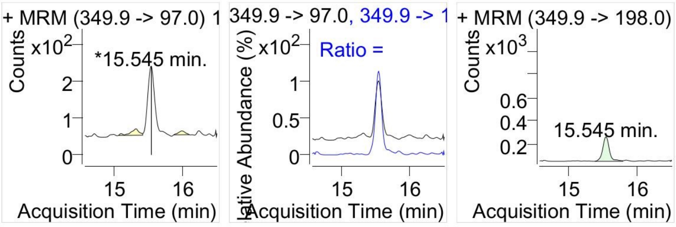

By Sample Quant Report

Analysis Info

**Instrument**	LCMS	**Operator**	
**Data File**	1365-PES2-24.d	**Sample Name**	1365-PES2-24
**Sample Type**	Sample	**Dilution**	0.003
**Acq. Method**	Pesticides_MRM_EN-15662_2024-06-27.m	**Acq. Date**	9/13/2024 11:07:32 AM
**Position**	P1-C4		-1

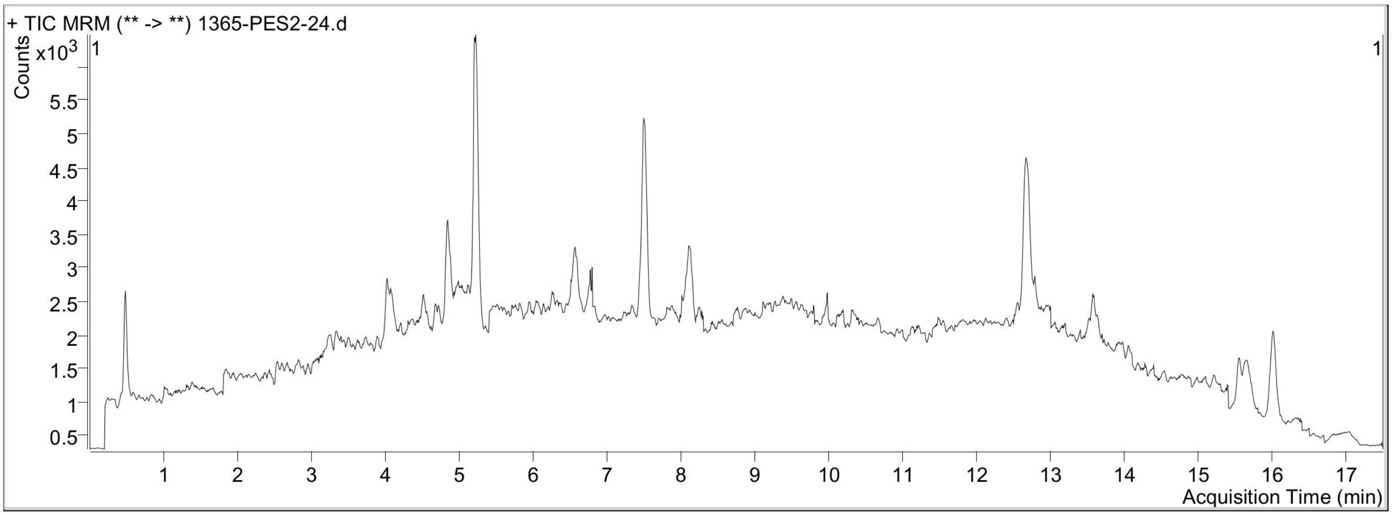

Quantitation Results

Compound	RT	Ref RT	Transition(T)	Transition(Q)	T-Resp	Q-Resp	QRatio	Ref QRatio	Final Conc.	Units
Methamidophos	0.48	0.76	142.0 -> 93.9	142.0 -> 124.9	0	691		31.6	0.00	ng/ml
Acephate	1.08	0.99	184.0 -> 94.6	184.0 -> 95.0	0	16		69.3	0.00	ng/ml
Carbendazim	2.39	2.31	192.1 -> 160.1	192.1 -> 132.1	0			16.8	0.00	ng/ml
Methomyl	3.34	3.34	163.1 -> 88.0	163.1 -> 106.0	0	49		64.3	0.00	ng/ml
Monocrotophos	3.87	3.85	224.1 -> 127.0	224.1 -> 58.0	0	207		51.6	0.00	ng/ml
Thiamethoxam	4.09	4.19	292.0 -> 211.1	292.0 -> 181.1	0	252		47.7	0.00	ng/ml
Clothianidin	4.51	4.59	250.0 -> 169.0	250.0 -> 131.9	0	701		77.4	0.00	ng/ml
Imidacloprid	4.68	4.75	256.0 -> 175.0	256.0 -> 208.9	0	451		86.8	0.00	ng/ml
Dimethoate	4.82	4.85	230.0 -> 198.8	230.0 -> 125.0	0	65		99.7	0.00	ng/ml
Acetamiprid	4.97	5.02	223.1 -> 126.0	223.1 -> 56.0	0	259		45.9	0.00	ng/ml
Sulfoxaflor	5.65	5.72	278.0 -> 174.0	278.0 -> 154.0	0	53		44.5	0.00	ng/ml
Amicarbazone	5.76	5.86	242.2 -> 143.1	242.2 -> 54.9	0	530		4.0	0.00	ng/ml
Ametryn	5.22	5.95	228.1 -> 186.1	228.1 -> 91.1	0	505		22.9	0.00	ng/ml
Bensulfuron-methyl	6.24	6.17	411.1 -> 182.1	411.1 -> 149.1	0	397		36.8	0.00	ng/ml
Nicosulfuron	5.76	6.17	411.1 -> 182.0	411.1 -> 181.9	0	15		100.0	0.00	ng/ml
Pyrimethanil	6.49	6.44	200.1 -> 82.0	200.1 -> 106.9	0	24		90.0	0.00	ng/ml
Terbutryn	7.25	7.23	242.1 -> 186.1	242.1 -> 68.1	0	325		31.4	0.00	ng/ml
Atrazine	7.50	7.53	216.1 -> 174.1	216.1 -> 68.0	9492	5582	58.8	61.0	0.01	ng/ml
Spiroxamine	8.01	8.10	298.3 -> 144.1	298.3 -> 100.1	0	66		56.0	0.00	ng/ml
Metalaxyl	8.11	8.14	280.2 -> 220.1	280.2 -> 160.1	0	2707		85.4	0.00	ng/ml
Triadimenol	9.45	9.69	296.1 -> 70.0	296.1 -> 99.1	0	77		4.6	0.00	ng/ml
Tebuconazole	9.79	9.81	308.1 -> 70.0	308.1 -> 124.9	0	48		1.1	0.00	ng/ml
Prochloraz	9.76	9.82	376.0 -> 308.0	376.0 -> 265.9	0			12.8	0.00	ng/ml
Dimethomorph	9.88	9.85	388.1 -> 165.1	388.1 -> 301.1	0	30		96.7	0.00	ng/ml
Promecarb	10.33	10.24	208.1 -> 109.1	208.1 -> 151.1	0	72		95.6	0.00	ng/ml
Triadimefon	10.83	10.85	294.1 -> 197.2	294.1 -> 225.1	0	36		1.0	0.00	ng/ml
Boscalid	11.20	10.93	343.0 -> 307.1	343.0 -> 271.2	0	31		50.8	0.00	ng/ml
Metolachlor	11.54	11.53	284.1 -> 252.1	284.1 -> 176.1	0	27		49.4	0.00	ng/ml
Emamectin benzoate		11.62	886.5 -> 158.0	886.5 -> 302.4				1.3	ND	ng/ml
Azinphos-Ethyl	11.94	12.10	346.1 -> 97.0	346.1 -> 137.0	0	26		69.7	0.00	ng/ml
Tebufenozide	12.07	12.20	353.2 -> 133.1	353.2 -> 297.2	0			38.8	0.00	ng/ml
Chlorfenvinphos	12.39	12.41	359.0 -> 99.0	359.0 -> 170.0	0			56.0	0.00	ng/ml
Aclonifen	12.66	12.47	265.0 -> 248.0	265.0 -> 182.1	0	39		69.4	0.00	ng/ml
Pirimifos-methyl	12.55	12.58	306.2 -> 164.1	306.2 -> 108.1	0	63		56.3	0.00	ng/ml
Kresoxim methyl	12.40	12.71	314.1 -> 222.1	314.1 -> 267.0	0	31		92.4	0.00	ng/ml
Difenoconazole	12.84	12.82	406.1 -> 251.0	406.1 -> 337.0	0			12.9	0.00	ng/ml

By Sample Quant Report

Quantitation Results

Fluopyram	13.10	13.06	397.0 -> 173.0	397.0 -> 145.0	11	41	365.6	68.3	0.00	ng/ml
Trifloxystrobin	14.45	14.46	409.1 -> 186.0	409.1 -> 145.0	0			49.9	0.00	ng/ml
Indoxacarb	14.63	14.48	528.1 -> 150.0	528.1 -> 203.0	0	35		90.3	0.00	ng/ml
Propaquizafop	14.85	14.72	444.1 -> 100.1	444.1 -> 299.2	0	6		14.0	0.00	ng/ml
Allethrin	15.22	15.24	303.2 -> 135.0	303.2 -> 123.0	0	104		39.0	0.00	ng/ml
Pyriproxyfen	15.21	15.25	322.2 -> 96.0	322.2 -> 185.0	0	102		17.7	0.00	ng/ml
Fluazinam	15.49	15.29	465.0 -> 373.0	465.0 -> 338.0	0	7		13.5	0.00	ng/ml
Chlorpyrifos	15.55	15.58	349.9 -> 97.0	349.9 -> 198.0	0	1677		76.8	0.00	ng/ml

By Sample Quant Report

Compound Graphics

Methamidophos

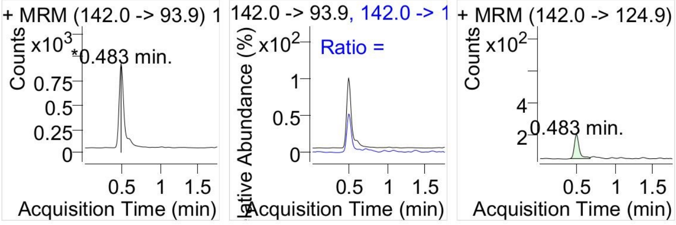

Acephate

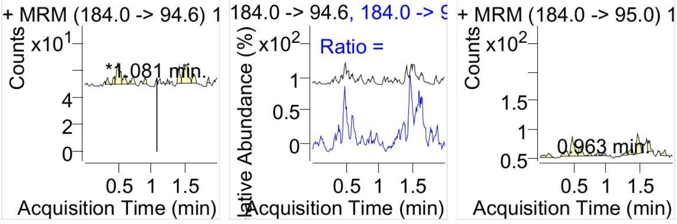

Carbendazim

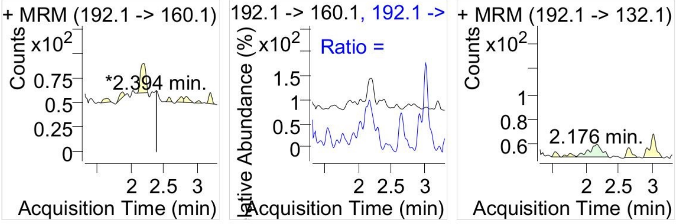

Methomyl

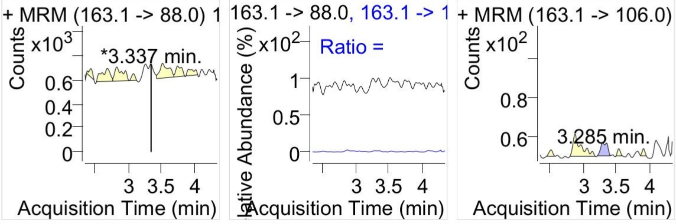

Monocrotophos

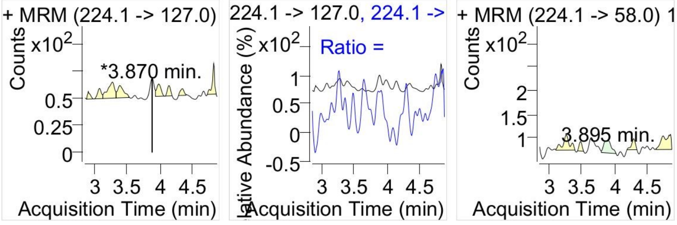

Thiamethoxam

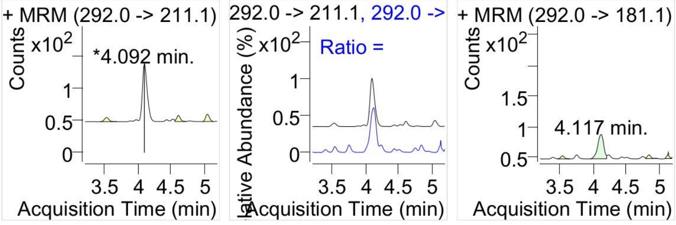

Clothianidin

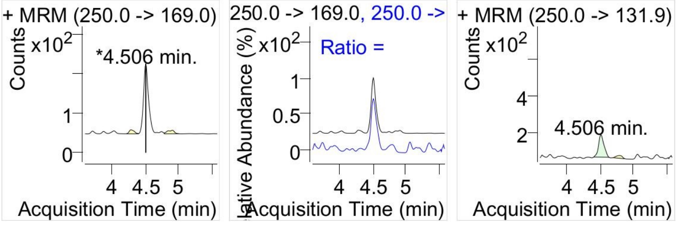

Imidacloprid

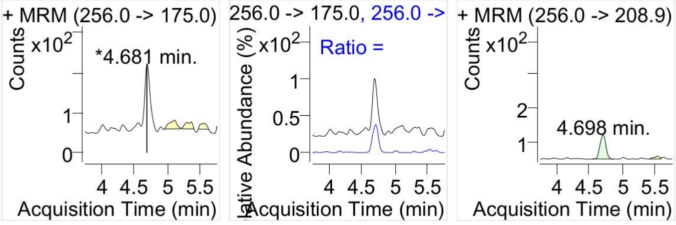

Dimethoate

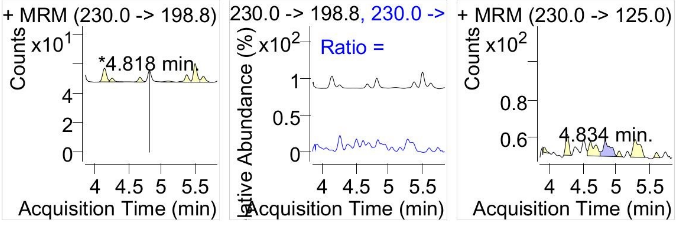

Acetamiprid

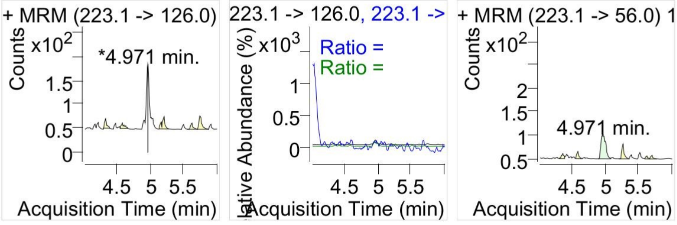

Sulfoxaflor

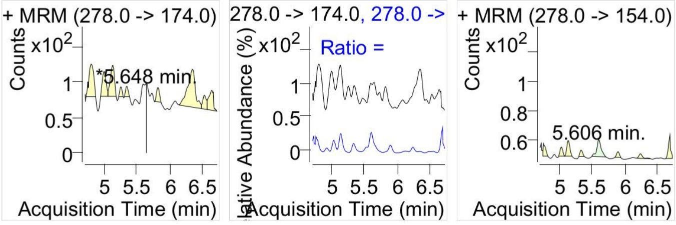

Amicarbazone

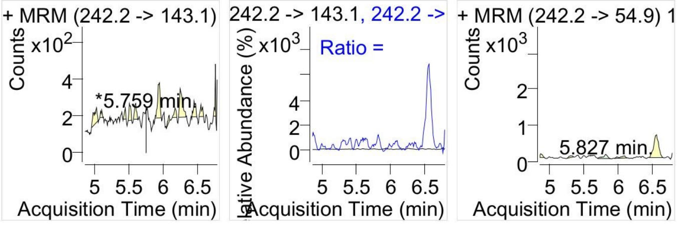

By Sample Quant Report

Ametryn

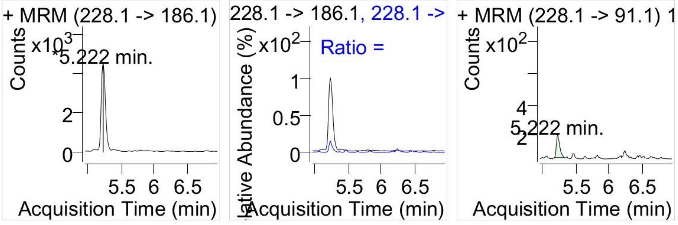

Bensulfuron-methyl

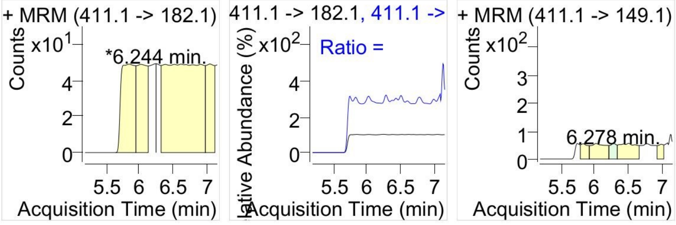

Nicosulfuron

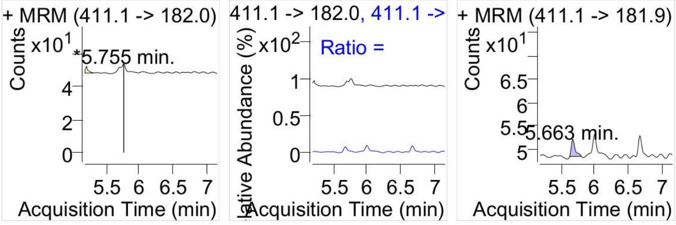

Pyrimethanil

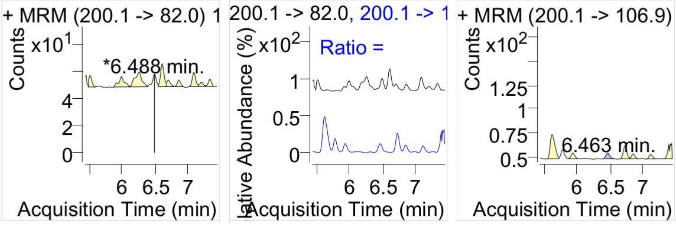

Terbutryn

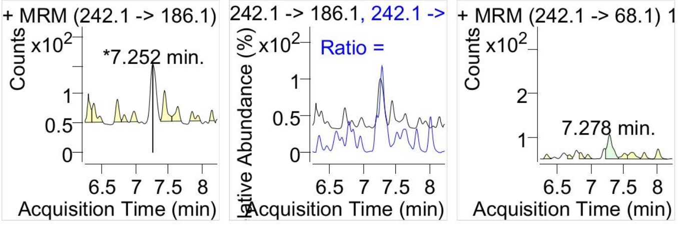

Atrazine

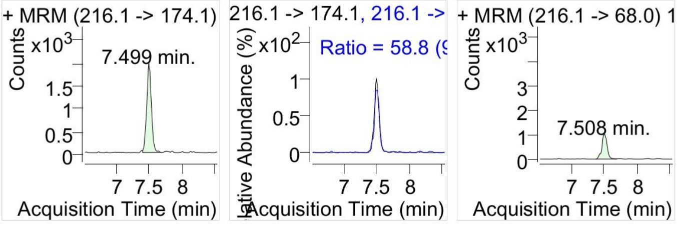

Spiroxamine

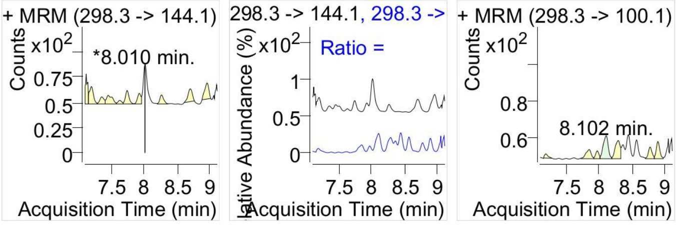

Metalaxyl

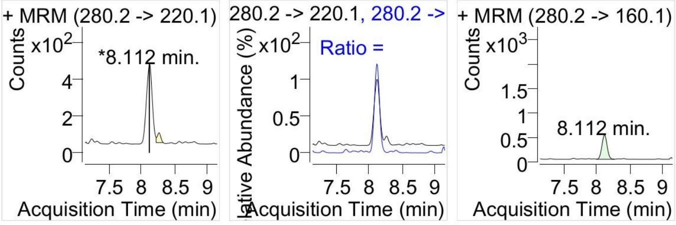

Triadimenol

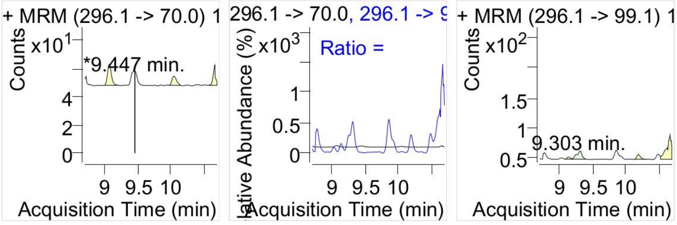

Tebuconazole

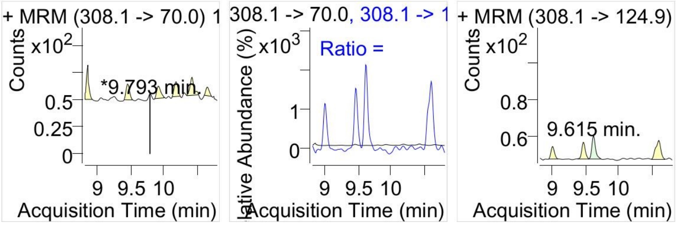

Prochloraz

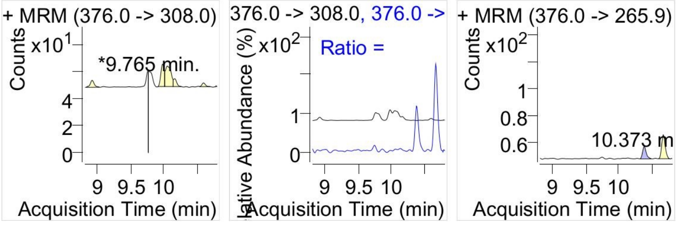

Dimethomorph

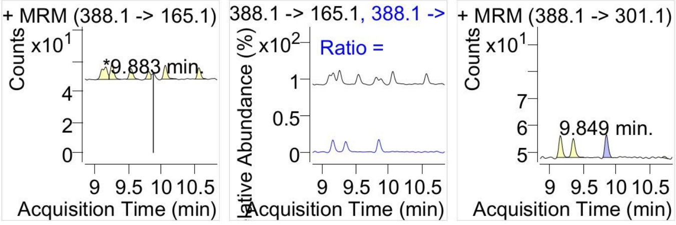

 Generated at 10:50 AM on 6/10/2025

By Sample Quant Report

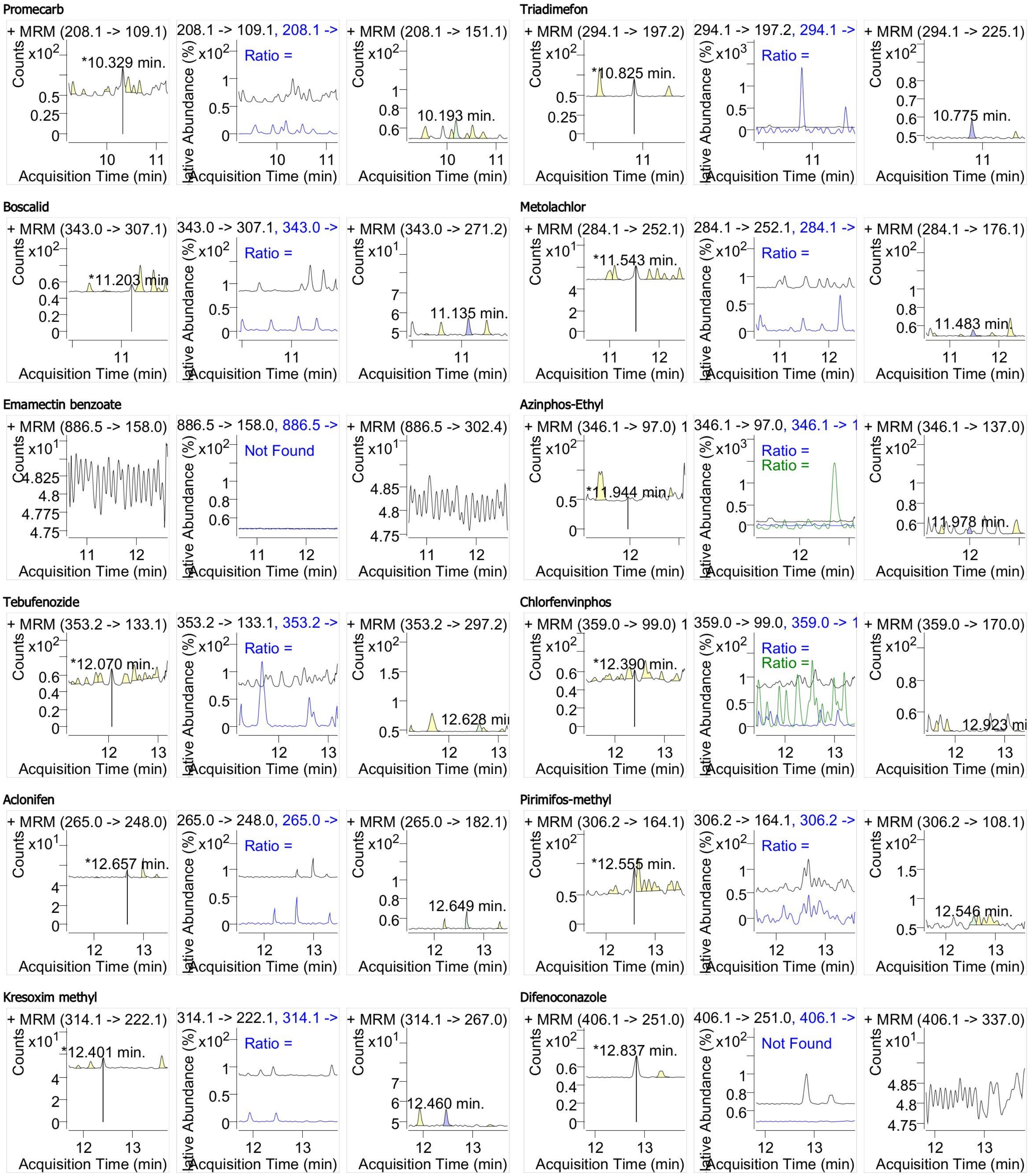

Fluopyram

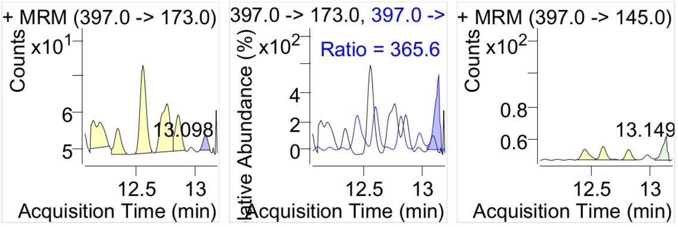

Trifloxystrobin

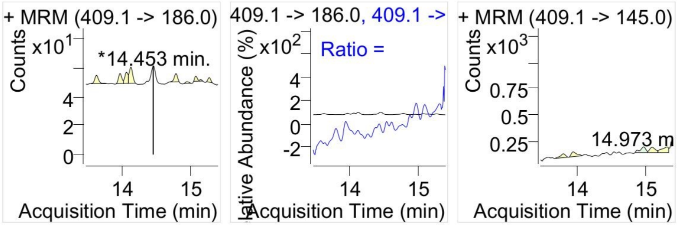

Indoxacarb

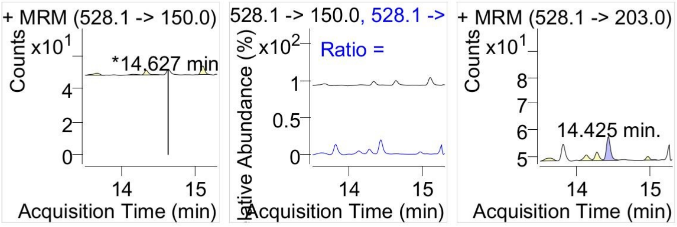

Propaquizafop

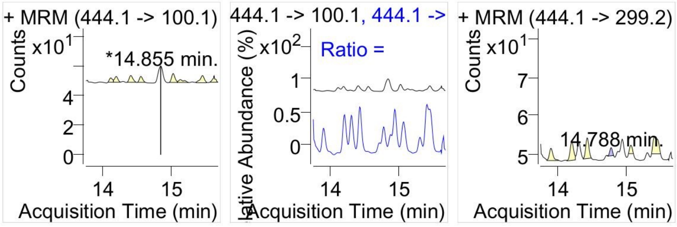

Allethrin

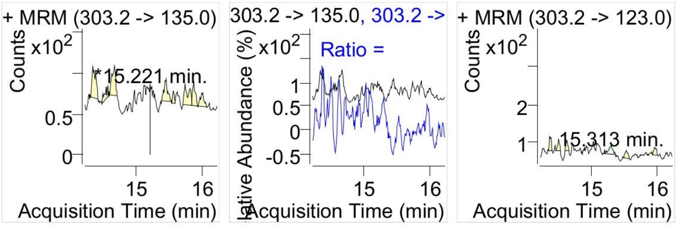

Pyriproxyfen

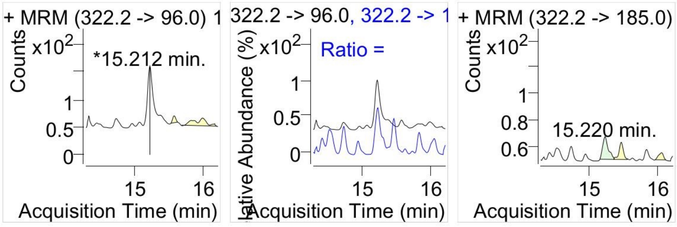

Fluazinam

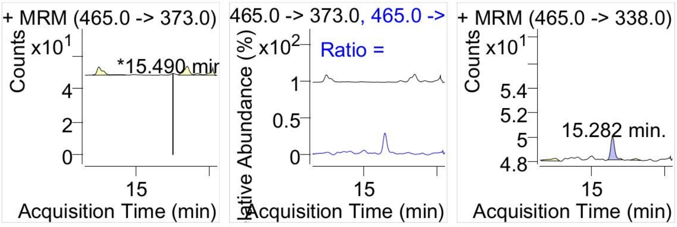

Chlorpyrifos

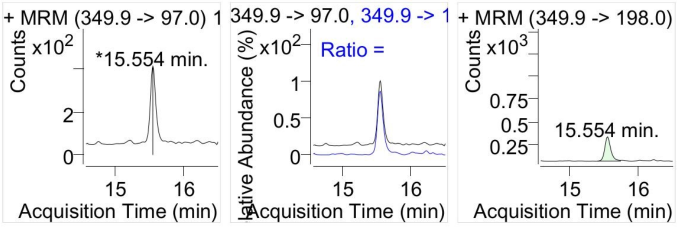

By Sample Quant Report

Analysis Info

**Instrument**	LCMS	**Operator**	
**Data File**	1366-PES2-24.d	**Sample Name**	1366-PES2-24
**Sample Type**	Sample	**Dilution**	0.003
**Acq. Method**	Pesticides_MRM_EN-15662_2024-06-27.m	**Acq. Date**	9/13/2024 11:30:23 AM
**Position**	P1-C5		-1

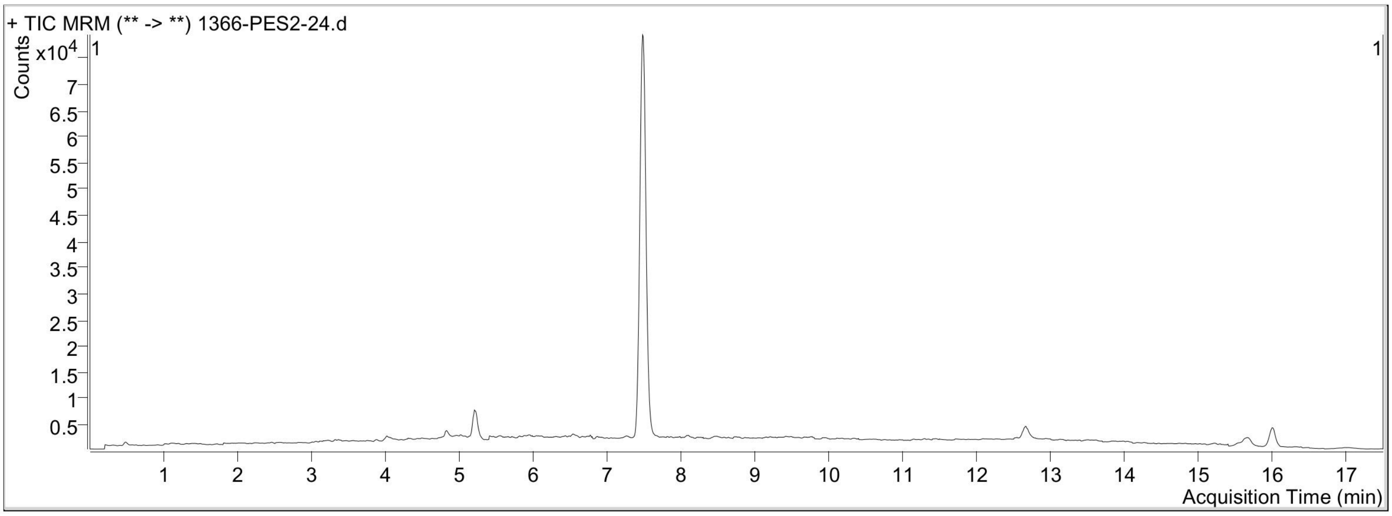

Quantitation Results

Compound	RT	Ref RT	Transition(T)	Transition(Q)	T-Resp	Q-Resp	QRatio	Ref QRatio	Final Conc.	Units
Methamidophos	0.62	0.76	142.0 -> 93.9	142.0 -> 124.9	0	69		31.6	0.00	ng/ml
Acephate	0.98	0.99	184.0 -> 94.6	184.0 -> 95.0	0	46		69.3	0.00	ng/ml
Carbendazim	2.14	2.31	192.1 -> 160.1	192.1 -> 132.1	0	115		16.8	0.00	ng/ml
Methomyl	3.36	3.34	163.1 -> 88.0	163.1 -> 106.0	0	27		64.3	0.00	ng/ml
Monocrotophos	3.75	3.85	224.1 -> 127.0	224.1 -> 58.0	0			51.6	0.00	ng/ml
Thiamethoxam	4.06	4.19	292.0 -> 211.1	292.0 -> 181.1	0	122		47.7	0.00	ng/ml
Clothianidin	4.58	4.59	250.0 -> 169.0	250.0 -> 131.9	0			77.4	0.00	ng/ml
Imidacloprid	4.77	4.75	256.0 -> 175.0	256.0 -> 208.9	0	146		86.8	0.00	ng/ml
Dimethoate	4.76	4.85	230.0 -> 198.8	230.0 -> 125.0	0	107		99.7	0.00	ng/ml
Acetamiprid	4.95	5.02	223.1 -> 126.0	223.1 -> 56.0	0	513		45.9	0.00	ng/ml
Sulfoxaflor	5.82	5.72	278.0 -> 174.0	278.0 -> 154.0	0	7		44.5	0.00	ng/ml
Amicarbazone	5.91	5.86	242.2 -> 143.1	242.2 -> 54.9	0	168		4.0	0.00	ng/ml
Ametryn	5.21	5.95	228.1 -> 186.1	228.1 -> 91.1	0	689		22.9	0.00	ng/ml
Bensulfuron-methyl	6.18	6.17	411.1 -> 182.1	411.1 -> 149.1	0	1838		36.8	0.00	ng/ml
Nicosulfuron	6.10	6.17	411.1 -> 182.0	411.1 -> 181.9	0	19		100.0	0.00	ng/ml
Pyrimethanil	6.44	6.44	200.1 -> 82.0	200.1 -> 106.9	0	38		90.0	0.00	ng/ml
Terbutryn	7.29	7.23	242.1 -> 186.1	242.1 -> 68.1	0	330		31.4	0.00	ng/ml
Atrazine	7.48	7.53	216.1 -> 174.1	216.1 -> 68.0	279506	171079	61.2	61.0	0.31	ng/ml
Spiroxamine	7.87	8.10	298.3 -> 144.1	298.3 -> 100.1	0	22		56.0	0.00	ng/ml
Metalaxyl	8.10	8.14	280.2 -> 220.1	280.2 -> 160.1	0	809		85.4	0.00	ng/ml
Triadimenol	9.66	9.69	296.1 -> 70.0	296.1 -> 99.1	0	19		4.6	0.00	ng/ml
Tebuconazole	9.62	9.81	308.1 -> 70.0	308.1 -> 124.9	0	27		1.1	0.00	ng/ml
Prochloraz	9.90	9.82	376.0 -> 308.0	376.0 -> 265.9	0	20		12.8	0.00	ng/ml
Dimethomorph	9.71	9.85	388.1 -> 165.1	388.1 -> 301.1	0	3		96.7	0.00	ng/ml
Promecarb	10.27	10.24	208.1 -> 109.1	208.1 -> 151.1	0	23		95.6	0.00	ng/ml
Triadimefon	10.87	10.85	294.1 -> 197.2	294.1 -> 225.1	0			1.0	0.00	ng/ml
Boscalid	11.08	10.93	343.0 -> 307.1	343.0 -> 271.2	0	44		50.8	0.00	ng/ml
Metolachlor	11.52	11.53	284.1 -> 252.1	284.1 -> 176.1	0	119		49.4	0.00	ng/ml
Emamectin benzoate	12.23	11.62	886.5 -> 158.0	886.5 -> 302.4	0	2		1.3	0.00	ng/ml
Azinphos-Ethyl	12.16	12.10	346.1 -> 97.0	346.1 -> 137.0	0	76		69.7	0.00	ng/ml
Tebufenozide	12.31	12.20	353.2 -> 133.1	353.2 -> 297.2	0	32		38.8	0.00	ng/ml
Chlorfenvinphos	12.49	12.41	359.0 -> 99.0	359.0 -> 170.0	0			56.0	0.00	ng/ml
Aclonifen	13.36	12.47	265.0 -> 248.0	265.0 -> 182.1	0	5		69.4	0.00	ng/ml
Pirimifos-methyl	12.71	12.58	306.2 -> 164.1	306.2 -> 108.1	0	115		56.3	0.00	ng/ml
Kresoxim methyl	13.52	12.71	314.1 -> 222.1	314.1 -> 267.0	0	8		92.4	0.00	ng/ml
Difenoconazole	12.85	12.82	406.1 -> 251.0	406.1 -> 337.0	0	39		12.9	0.00	ng/ml

By Sample Quant Report

Quantitation Results

Fluopyram	13.02	13.06	397.0 -> 173.0	397.0 -> 145.0	0			68.3	0.00	ng/ml
Trifloxystrobin	14.39	14.46	409.1 -> 186.0	409.1 -> 145.0	0			49.9	0.00	ng/ml
Indoxacarb	14.27	14.48	528.1 -> 150.0	528.1 -> 203.0	0	3		90.3	0.00	ng/ml
Propaquizafop	14.91	14.72	444.1 -> 100.1	444.1 -> 299.2	0	62		14.0	0.00	ng/ml
Allethrin	15.30	15.24	303.2 -> 135.0	303.2 -> 123.0	0	53		39.0	0.00	ng/ml
Pyriproxyfen	15.21	15.25	322.2 -> 96.0	322.2 -> 185.0	0	108		17.7	0.00	ng/ml
Fluazinam	15.25	15.29	465.0 -> 373.0	465.0 -> 338.0	0	26		13.5	0.00	ng/ml
Chlorpyrifos	15.54	15.58	349.9 -> 97.0	349.9 -> 198.0	0	1380		76.8	0.00	ng/ml

By Sample Quant Report

Compound Graphics

Methamidophos

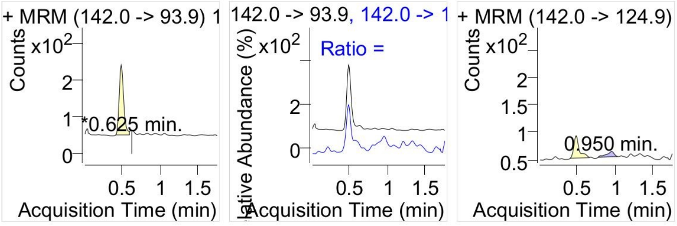

Acephate

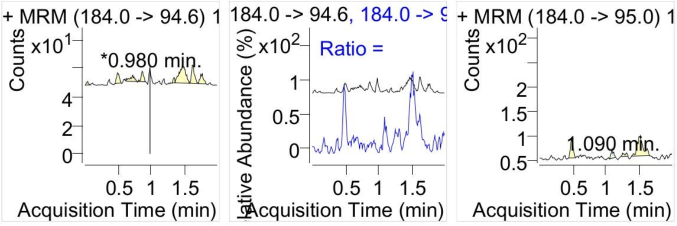

Carbendazim

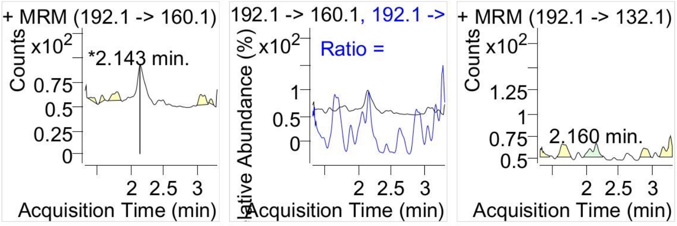

Methomyl

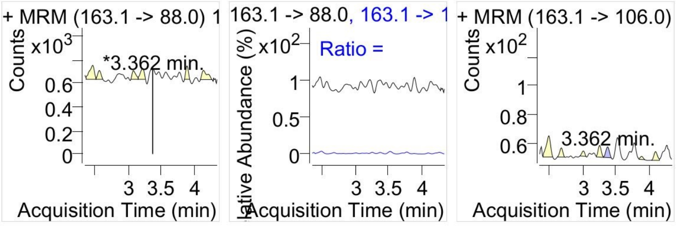

Monocrotophos

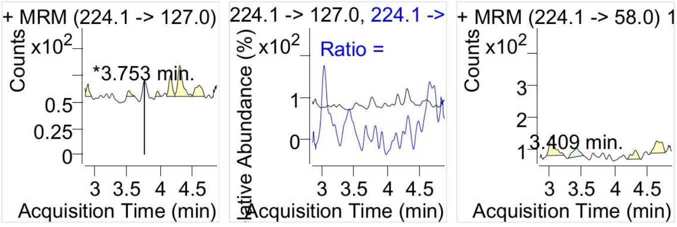

Thiamethoxam

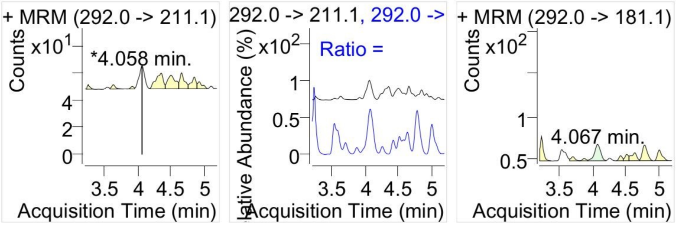

Clothianidin

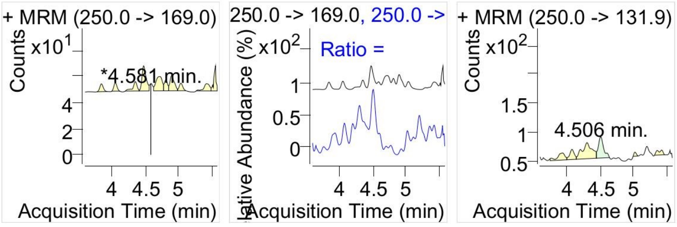

Imidacloprid

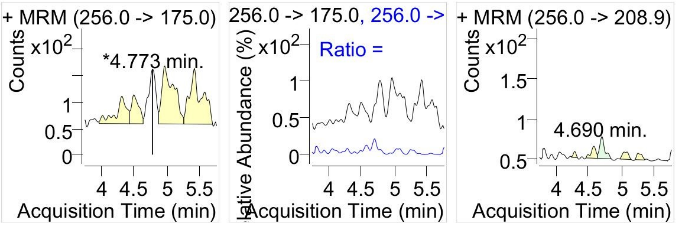

Dimethoate

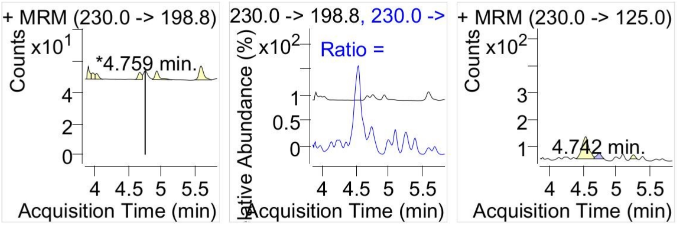

Acetamiprid

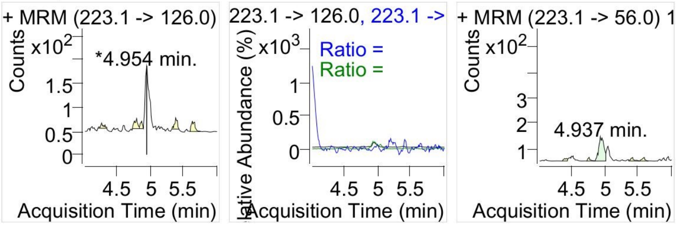

Sulfoxaflor

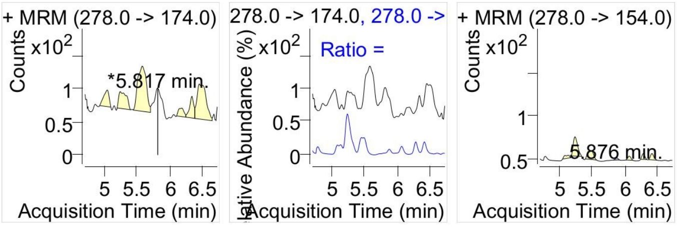

Amicarbazone

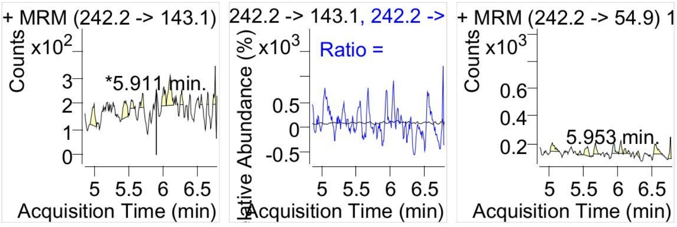

By Sample Quant Report

Ametryn

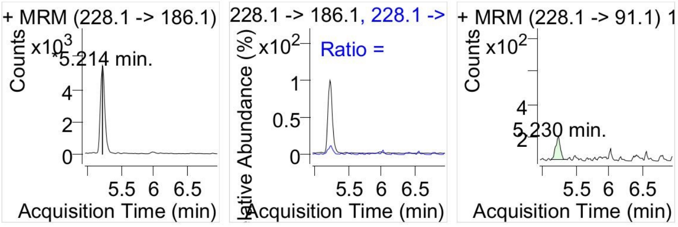

Bensulfuron-methyl

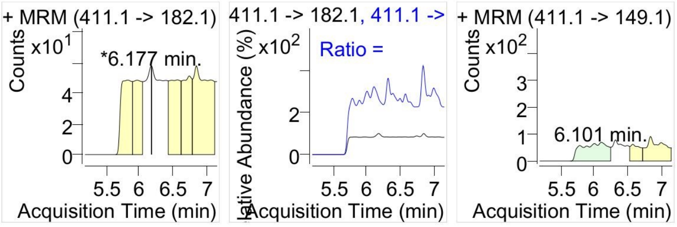

Nicosulfuron

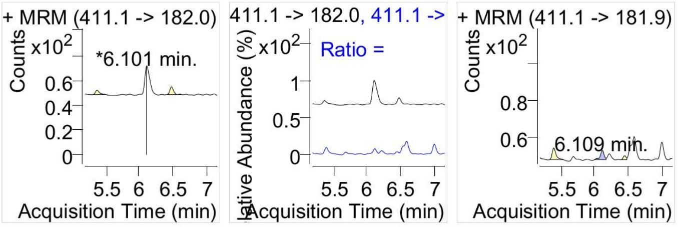

Pyrimethanil

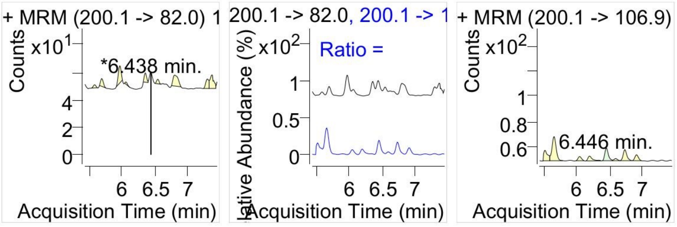

Terbutryn

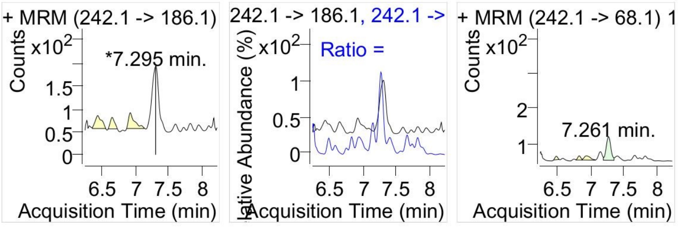

Atrazine

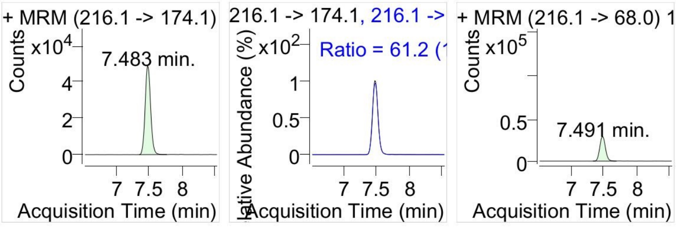

Spiroxamine

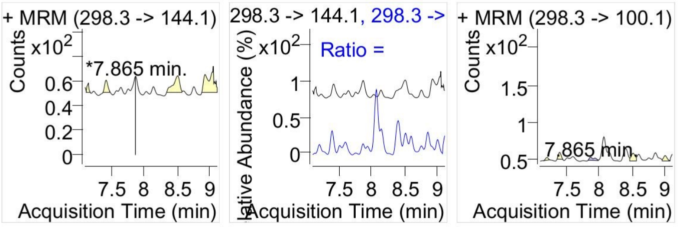

Metalaxyl

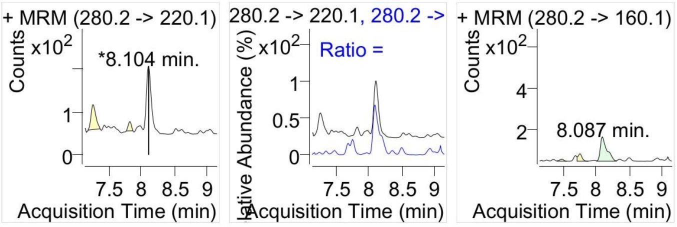

Triadimenol

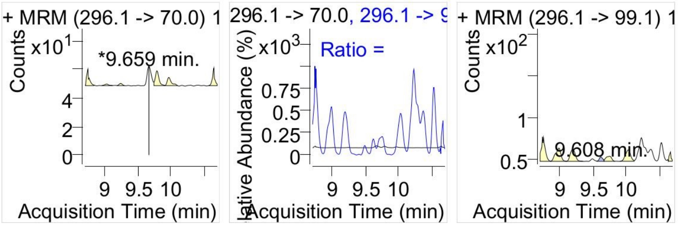

Tebuconazole

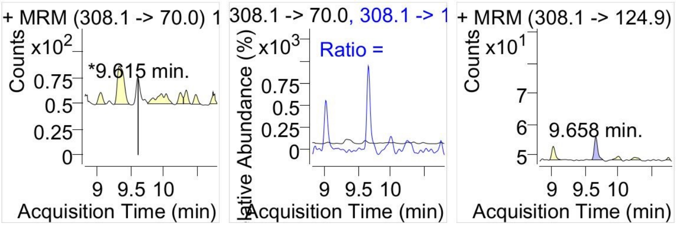

Prochloraz

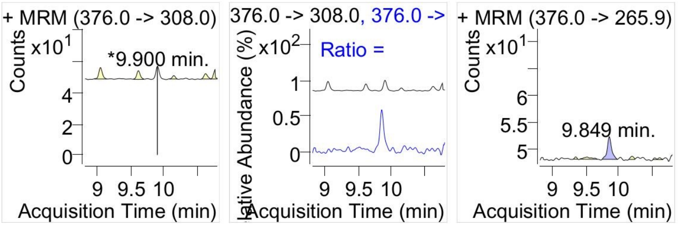

Dimethomorph

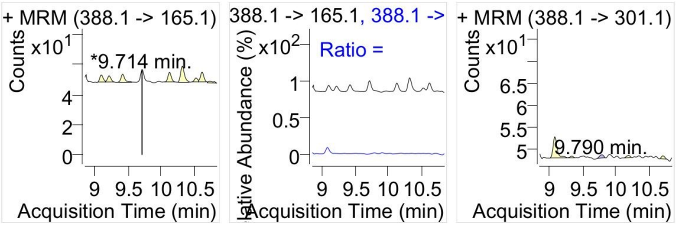

Promecarb

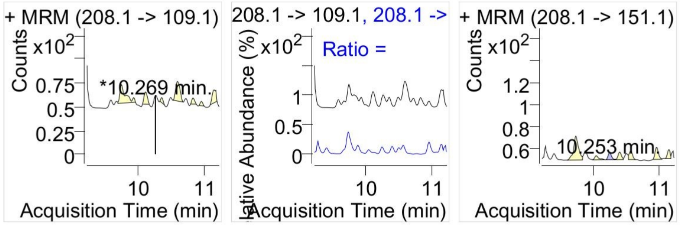

Triadimefon

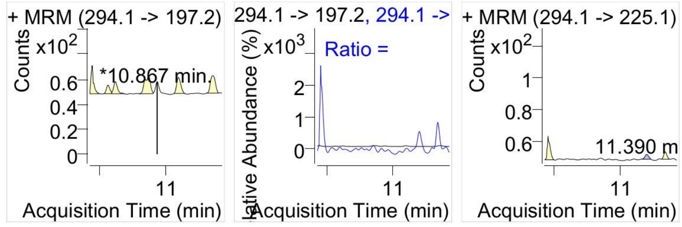

Boscalid

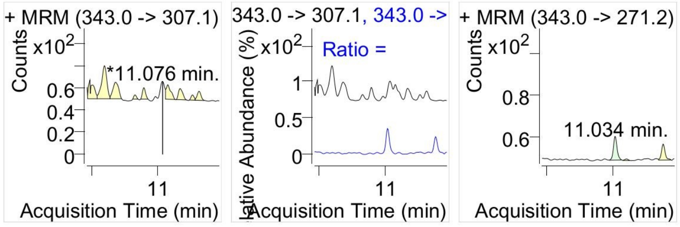

Metolachlor

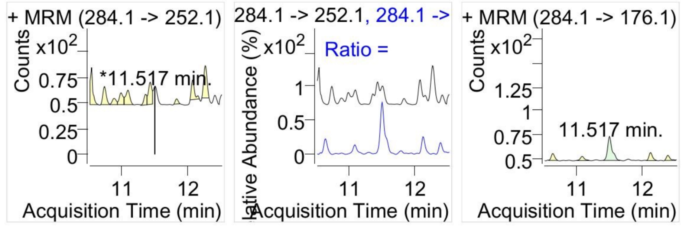

Emamectin benzoate

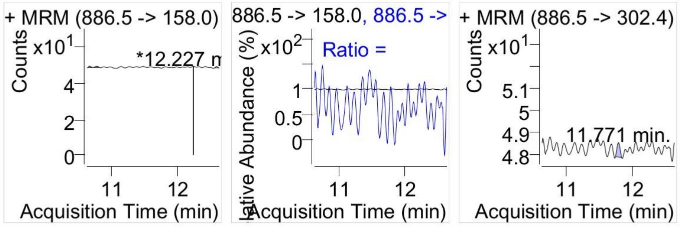

Azinphos-Ethyl

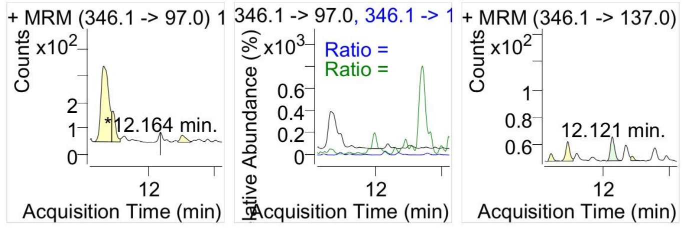

Tebufenozide

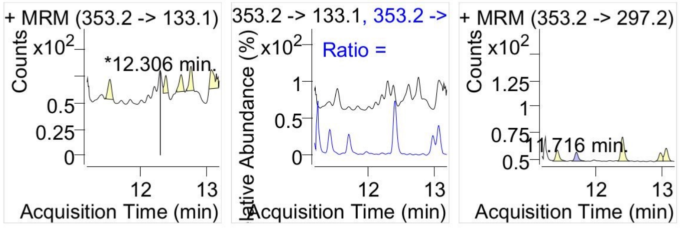

Chlorfenvinphos

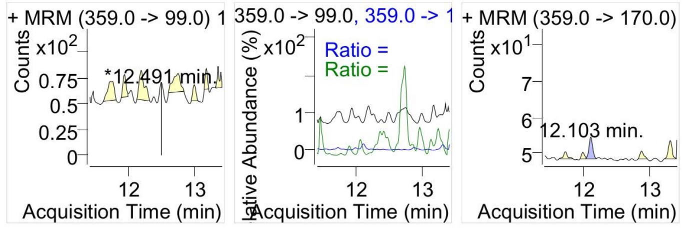

Aclonifen

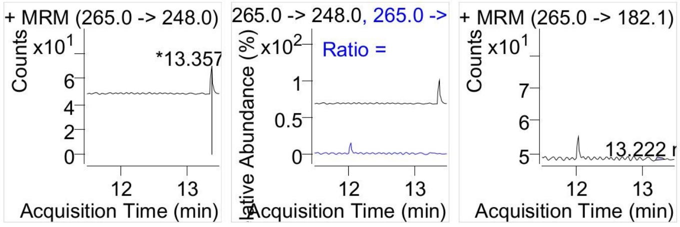

Pirimifos-methyl

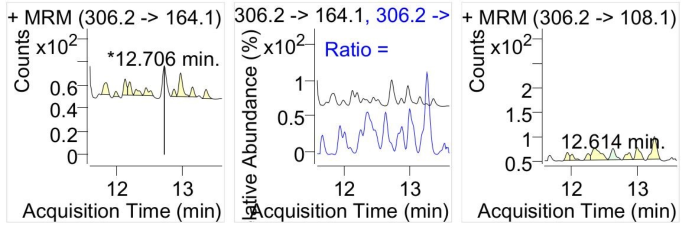

Kresoxim methyl

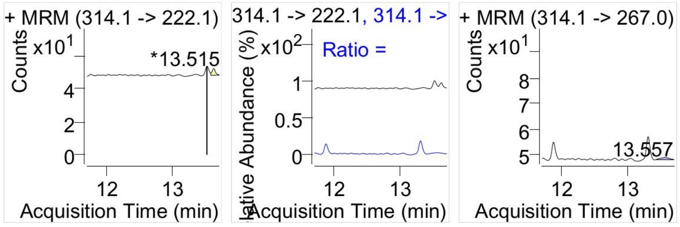

Difenoconazole

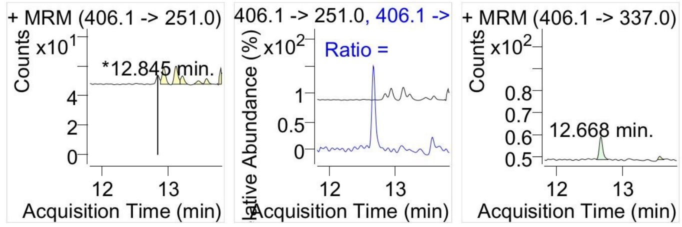

By Sample Quant Report

Fluopyram

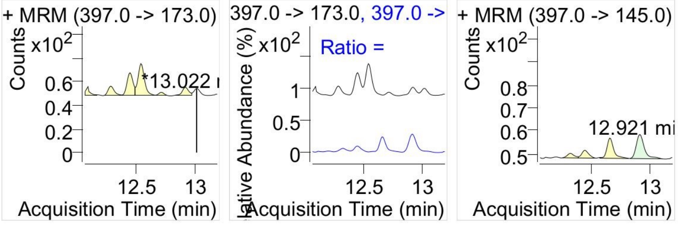

Trifloxystrobin

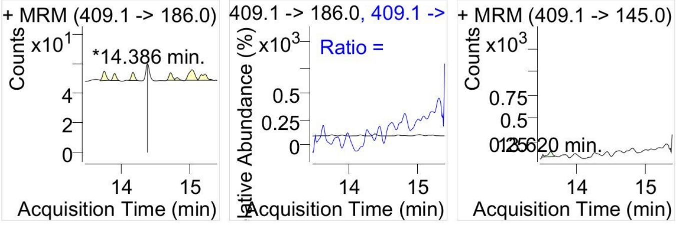

Indoxacarb

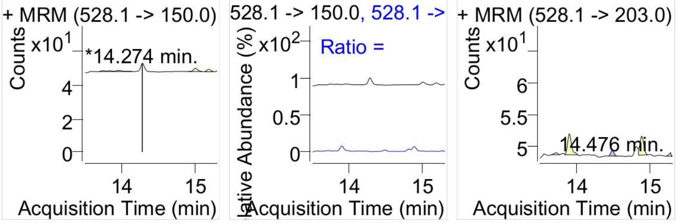

Propaquizafop

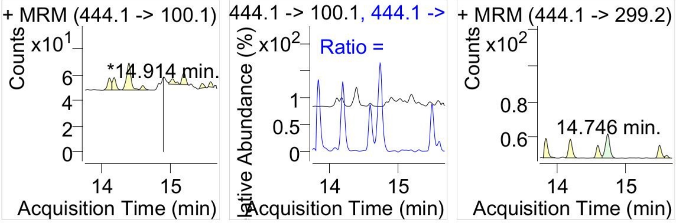

Allethrin

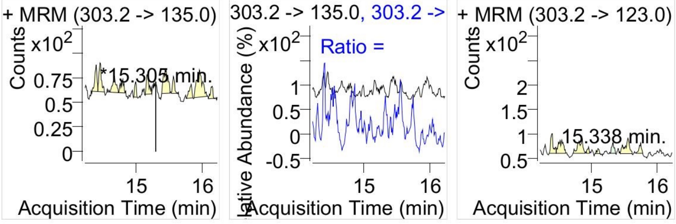

Pyriproxyfen

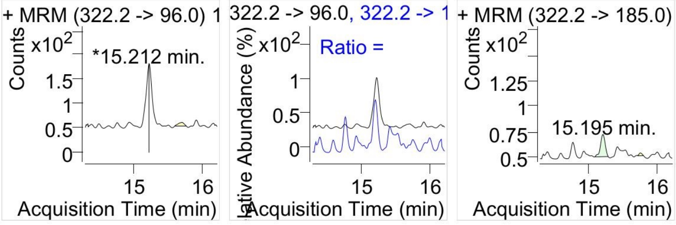

Fluazinam

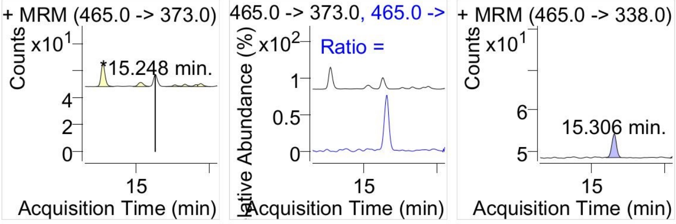

Chlorpyrifos

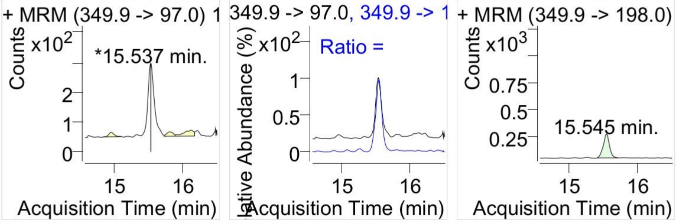

Supplement: Supplementary file 1 — Supplementary Material 1 [file 41598_2025_14239_MOESM1_ESM.pdf]
